# Efficacy and Safety of Advanced Therapies for Moderately to Severely Active Ulcerative Colitis at Induction and Maintenance: An Indirect Treatment Comparison Using Bayesian Network Meta-analysis

**DOI:** 10.1093/crocol/otad009

**Published:** 2023-03-01

**Authors:** Remo Panaccione, Eric B Collins, Gil Y Melmed, Severine Vermeire, Silvio Danese, Peter D R Higgins, Christina S Kwon, Wen Zhou, Dapo Ilo, Dolly Sharma, Yuri Sanchez Gonzalez, Si-Tien Wang

**Affiliations:** Inflammatory Bowel Disease Unit, Division of Gastroenterology and Hepatology, University of Calgary, Calgary, Alberta, Canada; Medicus Economics LLC, Milton, Massachusetts, USA; Cedars-Sinai Medical Center, Los Angeles, California, USA; Department of Gastroenterology & Hepatology, University Hospital Leuven, KU Leuven, Leuven, Belgium; Gastroenterology and Endoscopy, IRCCS Ospedale San Raffaele, Department of Gastroenterology, University Vita-Salute San Raffaele, Milan, Italy; Department of Medicine, Division of Gastroenterology, University of Michigan, Ann Arbor, Michigan, USA; Cytel, Inc., Waltham, Massachusetts, USA; AbbVie Inc., North Chicago, Illinois, USA; AbbVie Inc., North Chicago, Illinois, USA; AbbVie Inc., North Chicago, Illinois, USA; AbbVie Inc., North Chicago, Illinois, USA; Medicus Economics LLC, Milton, Massachusetts, USA

**Keywords:** ulcerative colitis, clinical trials, advanced therapies, network meta-analysis

## Abstract

**Background:**

Given rapid innovation in advanced therapies for moderately to severely active ulcerative colitis (UC), we investigated their comparative efficacy and safety during induction and maintenance through network meta-analysis.

**Methods:**

Using Bayesian methods, endpoints of clinical remission and clinical response per Full Mayo score, and endoscopic improvement were assessed in bio-naive and -exposed populations. Safety was assessed in overall populations by all adverse events (AEs), serious AEs, discontinuation due to AEs, and serious infections. Phase 3 randomized controlled trials were identified via systematic literature review, including the following advanced therapies: infliximab, adalimumab, vedolizumab, golimumab, tofacitinib, ustekinumab, filgotinib, ozanimod, and upadacitinib. Random effects models were used to address between-study heterogeneity. Intent-to-treat (ITT) efficacy rates were calculated by adjusting maintenance outcomes by likelihood of induction response.

**Results:**

Out of 48 trials identified, 23 were included. Across all outcomes and regardless of prior biologic exposure, ITT efficacy rates were highest for upadacitinib, owing to its highest ranking for all efficacy outcomes in induction and for all but clinical remission during maintenance among bio-naive induction responders. For all advanced therapies versus placebo, there were no significant differences in serious AEs or serious infections across therapies. For all AEs, golimumab had higher odds versus placebo during maintenance; for discontinuation due to AEs, upadacitinib had lower odds versus placebo during induction, while ustekinumab and vedolizumab had lower odds versus placebo during maintenance.

**Conclusions:**

Upadacitinib may be the most efficacious therapy for moderately to severely active UC based on ITT analyses, with similar safety across advanced therapies.

## Graphical Abstract

**Graphical Abstract:**
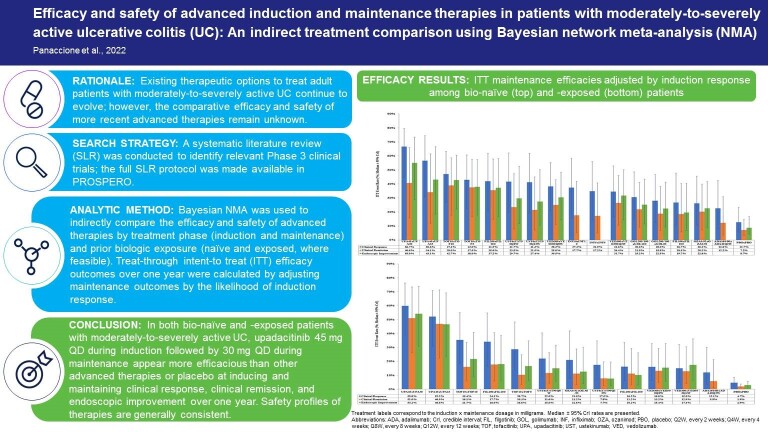

## Introduction

Ulcerative colitis (UC) is a chronic inflammatory bowel disease that affects the colorectum and is clinically characterized by bloody diarrhea, urgency, tenesmus, abdominal pain, malaise, weight loss, and fever. Disease onset commonly occurs between the ages of 15 and 30 years and the annual global incidence ranges from 9 to 20 cases per 100 000 per year with higher incidence in North America and Northern Europe.^1^

The advanced therapeutic armamentarium for adults with moderately to severely active UC is rapidly evolving. For over 20 years, biologics targeting specific inflammatory pathways have been the mainstay, beginning with those targeting tumor necrosis alpha (TNFi; eg, infliximab [INF], adalimumab [ADA], and golimumab [GOL]) followed by biologics with other modes of action (eg, vedolizumab [VED] and ustekinumab [UST]). However, treatment limitations for moderately to severely active UC remain, including primary nonresponse, secondary loss of response, immunogenicity, and parenteral administration.^2^ To overcome these limitations, there has been increasing interest in small molecule drugs (SMDs), which can be orally administered and lack immunogenicity.^3^ Of particular interest are SMDs that inhibit the Janus kinase (JAK)-mediated inflammatory pathway, of which tofacitinib (TOF), filgotinib (FIL), and upadacitinib (UPA) are currently approved for use in adults with moderately to severely active UC who have had an inadequate response or intolerance to conventional therapy and/or TNFi’s.^4–7^ All orally administered, TOF, FIL, and UPA are distinguished by their JAK selectivity: TOF is a pan-JAK inhibitor while FIL and UPA are approximately 30- and 60-fold selective for JAK1 over JAK2, respectively.^8,9^ In human cellular assays, UPA preferentially inhibits signaling by JAK1 or JAK1/3 with functional selectivity over cytokine receptors that signal via pairs of JAK2.5 In addition, ozanimod (OZA), a SMD that selectively modulates the sphingosine-1-phosphase receptor (S1P), is also approved for use in UC.^10^

With this rapid innovation, much attention has been paid toward establishing the comparative efficacy and safety of biologics and SMDs that are approved or in late stages of development for moderately to severely active UC. Most recently, Lasa et al^11^ and Burr et al^12^ each conducted and published a systematic literature review (SLR) and frequentist network meta-analysis (NMA) on the efficacy and safety of biologics and SMDs for patients with moderately to severely UC. Both studies assessed all outcomes after induction (6–14 weeks); Lasa et al^11^ additionally assessed efficacy after maintenance (26–66 weeks) separately for treat-through (TT) and re-randomized responders (RR) randomized clinical trials (RCTs). Both studies also assessed all outcomes in overall populations, as well as induction efficacy outcomes by prior biologic exposure, though UPA was excluded from subgroup analyses in Lasa et al^11^ due to lack of published data.

Despite these recent publications, there remain key gaps in our understanding of the comparative efficacy and safety of advanced therapies for moderately to severely active UC. First, an indirect comparison of maintenance treatment safety remains unpublished. Second, so far only separate NMAs have been conducted for induction and maintenance treatments, when in practice clinicians would consider the overall comparative efficacy of treatments across induction and maintenance in their decision-making. Finally, Lasa et al^11^ and Burr et al^12^ conducted their literature searches in July 2021 and October 2021, respectively, and more data have become available to address the research question, including more comprehensive UPA data from its phase 3 RCT. We therefore performed Bayesian NMAs to determine the latest comparative efficacy and safety of all currently approved biologics and SMDs for moderately to severely active UC. First, consistent with prior published NMAs, we conducted separate NMAs for induction and maintenance treatments.^11,12^ Then, rather than burdening medical decision makers with the task of reconciling results from disparate analyses of induction and maintenance, we took the novel additional step to combine induction and maintenance NMA results to simulate absolute treatment efficacy in an intent-to-treat (ITT) population of induction responders in an RR maintenance RCT.

## Materials and Methods

### Search Strategy

A clinical SLR was conducted per guidance from the Cochrane Handbook for Systematic Reviews of Interventions,^13^ Centre for Reviews and Dissemination’s Guidance for Undertaking Reviews in Healthcare,^14^ and Methods for the Development of National Institute for Health and Care Excellence (NICE) Public Health Guidance.^15^ Using the Ovid platform,^16^ searches for English-language publications of RCTs reporting the clinical efficacy and/or safety of relevant treatments for adults with moderately to severely active UC were conducted on January 6, 2022 (from inception of the databases) in MEDLINE, Embase, and other relevant databases. Keyword searches of the annual proceedings of relevant scientific meetings (from last 4 years) and clinical trial registers (no date limit) were also conducted for additional available data. Finally, the bibliographies of SLRs and meta-analyses identified through database searches and selected key RCTs were reviewed to ensure literature saturation. Details of the search strategy are presented in Appendix 1; the full SLR protocol has been registered with PROSPERO.^17^

### Study Selection

Title/abstract and full-text screenings were conducted sequentially and in parallel by 2 independent researchers to identify studies that met the SLR eligibility criteria, which are described in detail in Appendix 2. From the SLR, to meet the study objectives and minimize between-study heterogeneity, a narrower set of inclusion criteria was imposed for the NMA which is described in Table 1.

**Table 1.** Inclusion criteria from SLR to NMA.

Element	Inclusion
Patient population	• Randomized adults (≥16 years) with moderately to severely active UC
Interventions	Evaluated the following FDA- and/or EMA-approved biologic or SMD doses: • ADA (subcutaneous [SC] 160 mg at week 0, 80 mg at week 2, and 40 mg at week 4 [ADA160/80] for induction, and SC 40 mg every other week [ADA40Q2W] or every week [ADA40QW] for maintenance) • FIL (oral 100 mg [FIL100] or 200 mg [FIL200] QD for induction and maintenance) • GOL (SC 200 mg at week 0 and 100 mg at week 2 [GOL200/100] for induction, and SC 100 mg [GOL100] or 50 mg [GOL50] every 4 weeks for maintenance) • INF (IV 5 mg/kg [INF5] or 10 mg/kg [INF10^a^] at weeks 0, 2, and 6 then every 8 weeks for induction and maintenance) • OZA (oral 0.23 mg QD for 4 days, 0.46 mg QD for 3 days, then 0.92 mg QD [OZA0.92] for induction and maintenance) • TOF (oral 10 mg BID [TOF10] for 8 weeks for induction, and oral 5 mg BID [TOF5] or TOF10 for maintenance) • UPA (oral 45 mg QD for 8 weeks [UPA45] for induction, and oral 15 mg [UPA15] or 30 mg [UPA30] QD for maintenance) • UST (IV 6 mg/kg at week 0 [UST6] for induction, and SC 90 mg at week 8 then every 12 weeks [UST90Q12W] or 8 weeks [UST90Q8W] for maintenance) • VED (IV 300 mg at weeks 0, 2, and 6 [VED300] for induction, and IV 300 mg every 8 weeks [VED300Q8W] or every 4 weeks [VED300Q4W] for maintenance)
Comparators	• Included an active comparator that enabled network link or PBO
Outcomes measures	• Reported an outcome of interest after 6–10 weeks of induction treatment and/or after at least 40 weeks of maintenance treatment
Study design	• Double-blinded, phase 3+ RCT

Abbreviations: ADA, adalimumab; BID, twice daily; EMA, European Medicines Agency; FDA, US Food and Drug Administration; FIL, filgotinib; GOL, golimumab; INF, infliximab; IV, intravenous; NMA, network meta-analysis; OZA, ozanimod; PBO, placebo; QD, once daily; RCT, randomized clinical trial; SC, subcutaneous; SLR, systematic literature review; SMD, small molecule drug; TOF, tofacitinib; UC, ulcerative colitis; UPA, upadacitinib; UST, ustekinumab; VED, vedolizumab.

^a^Dose unapproved but used in clinical practice.

### Outcomes

Efficacy outcomes based on the Full Mayo score (FMS) were assessed as possible. The FMS consists of 4 items, each with a subscore that ranges from 0 to 3 whole points: stool frequency subscore (SFS), rectal bleeding subscore (RBS), endoscopic Mayo subscore (EMS), and physician’s global assessment (PGA).^18^ The efficacy outcomes analyzed by prior biologic exposure were clinical response (decrease from baseline in FMS ≥3 points and ≥30%, accompanied by a decrease in RBS of ≥1 or an absolute RBS ≤1), clinical remission (FMS ≤2 with no subscore >1), and endoscopic improvement (EMS ≤1). Deviations from these definitions are noted in Appendix 5. The safety outcomes analyzed in the overall populations were all adverse events (AEs), discontinuation due to AEs, serious AEs, and serious infections, as reported in the RCT publications. Only outcomes evaluated during randomized, double-blinded phases were considered. Each binary outcome was assessed after induction and maintenance, for a total of 20 NMAs conducted (ie, induction and maintenance of clinical response, clinical remission, and endoscopic improvement in bio-naive and -exposed populations; 4 induction and maintenance safety outcomes in overall populations).

### Data Extraction and Imputation

For each NMA-eligible RCT, relevant data for overall and bio-naive/exposed subgroups were extracted into an Excel database: study characteristics (eg, name, design, total randomized, duration), exposure definition (eg, drug, dose, and duration), baseline patient characteristics (eg, age, gender, weight, disease duration, extensive colitis or pancolitis, FMS, C-reactive protein, concurrent immunomodulators, and corticosteroids), and reported outcomes (eg, number assessed [*N*] and number with event [*n*]). Two researchers independently rated the quality of the included RCTs using the Cochrane Risk of Bias tool, version 2.0.^19^ For UPA, FMS outcomes were obtained from ad hoc analyses of its patient-level trial data.

While Lasa et al^11^ conducted separate NMAs on the efficacy of maintenance treatments assessed in TT versus RR trials, we instead combined them by adjusting the observed data from TT trials to mimic those of RR trials, based on the assumption that the number of responders at the end of induction is a proxy for the total number of patients entering maintenance. Thus, to the extent that they are reported, clinical outcomes for induction responder subset of TT trials were used. If induction responder data were not reported, then values were assumed per the Evidence Review Group (ERG) maintenance-only NMA in the NICE submission for UST in UC (TA633).^20^ Accepted and used by the NICE-commissioned ERG, this imputation had one noted drawback—it ignored nonresponders at the end of the induction phase who could have potentially become responders by the end of the maintenance phase.^20^ Details of the TT-to-RR adjustment are described in Appendix 6.

### Data Analysis

Per NICE guidelines, NMAs were conducted in a Generalized Linear Model (GLM) framework using a binomial likelihood, logit link, and Bayesian Markov chain Monte Carlo (MCMC) simulation with 3 chains of 100 000 posterior iterations each.^21^ Models were considered to converge when their Potential Scale Reduction Factor (PSRF) fell below 1.05.^22^ By default, independent baseline (placebo [PBO]) risks were assumed and noninformative prior distributions were used (normal [0, 100^2^] for continuous parameters and uniform [0, 5] for the between-study heterogeneity SD of a random effects [RE] model). Deviations from these defaults were made to account for data sparsity (eg, exchangeable baseline assumption when ≥1 PBO arms have zero events, slightly informative half-normal [0, 0.32^2^] prior for SD when ≥50% of treatments in a network are informed by a single RCT). RE models were utilized to account for expected heterogeneity in trial endpoints and study design. RE models adjusted for baseline risk were selected if the model successfully converged and if the associated meta-regression term was significant (95% credible interval [CrI] excluded zero). In networks with evidence loops, network consistency was assessed by comparing the fit of an RE unrelated mean effects (UME) model to the associated RE model NMA.^23^

From each logit NMA, pairwise odds ratios (ORs; median and 95% CrI), surface under the cumulative ranking curve (SUCRA) values, and absolute rates were estimated to inform relative treatment effect size, statistical significance, and treatment rankings. SUCRA serves as a numerical summary for the probability of each treatment being ranked first, second, third, and so on; SUCRA would be 100% for the most favorable treatment (ie, has the highest efficacy rate or lowest safety event rate) and 0% for the least favorable treatment in the network (ie, has the lowest efficacy rate or highest safety event rate).^24^ Absolute rates were estimated relative to PBO rates modeled using a baseline natural history RE model.^23^ Finally, to simulate treatment efficacies in an ITT population of an RR maintenance trial, MCMC chains of induction response rates were multiplied with those of maintenance efficacy rates to obtain ITT efficacy rates. From these ITT efficacy rates, and absolute event rates for safety outcomes, the numbers-needed-to-treat (NNTs) or -harm (NNHs) were estimated for UPA relative to each comparator as the inverse of rate difference between UPA and the comparator. All logit NMAs were run using Just Another Gibbs Sampler (JAGS; version 4.3.0) via the bnma package (version 1.4.0) in the R statistical software (R Foundation for Statistical Computing, Vienna, Austria; version 4.0.2).^25–27^

For networks with significant baseline risk heterogeneity, but baseline risk adjustment for the selected logit model did not converge or run due to data sparsity, a fixed effects (FE) model using a risk difference (RD) link was alternatively tested. The RD link is a valid though noncanonical method to potentially minimize the impact of PBO heterogeneity. Additional RD NMAs were conducted as needed to generate corresponding ITT efficacy rates in sensitivity analysis. These RD NMAs were run in WinBUGS.^23,28–30^

## Ethical Considerations

The Independent Ethics Committee or Institutional Review Board at each study site approved the study protocol, informed consent forms, and recruitment materials before patient enrollment for the UPA phase 3 clinical trials. These studies were conducted in accordance with the International Conference for Harmonisation guidelines, applicable regulations, and the Declaration of Helsinki. Patients provided written informed consent before screening. Data for all other advanced therapies were based on a SLR of published phase 3 studies.

## Results

### Study Selection and Characteristics

**Figure 1.**
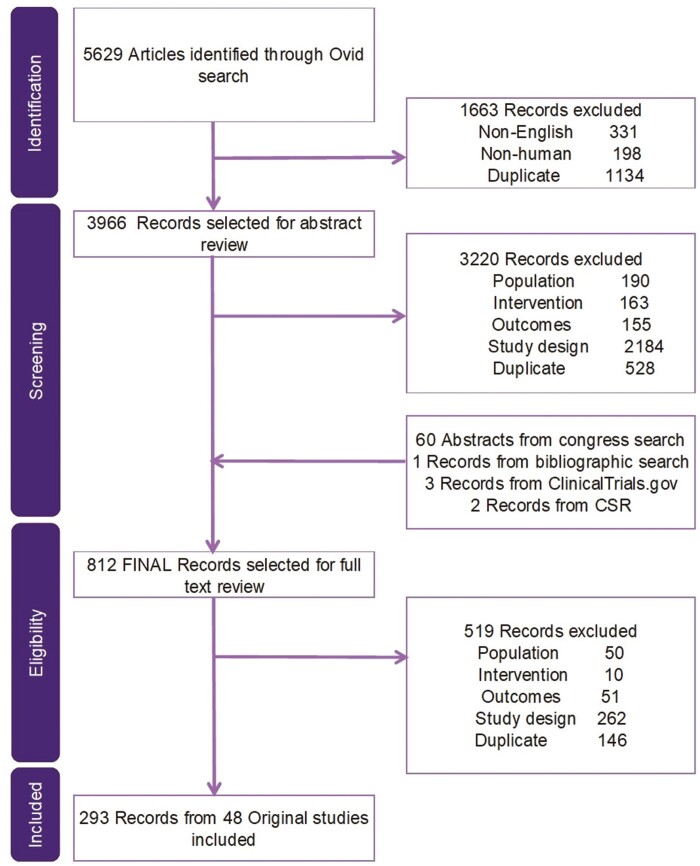
PRISMA diagram for clinical evidence.

**Figure 2.**
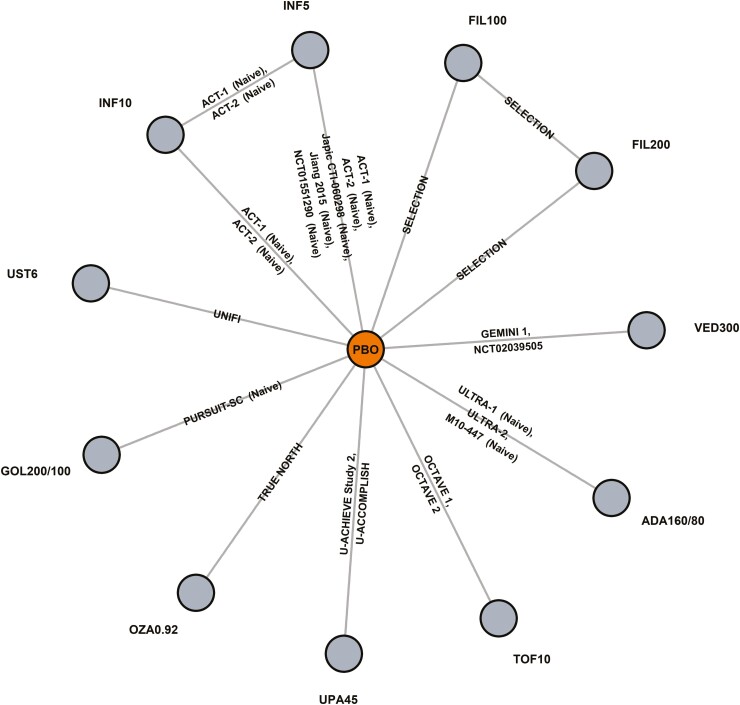
Induction network plot.

**Figure 3.**
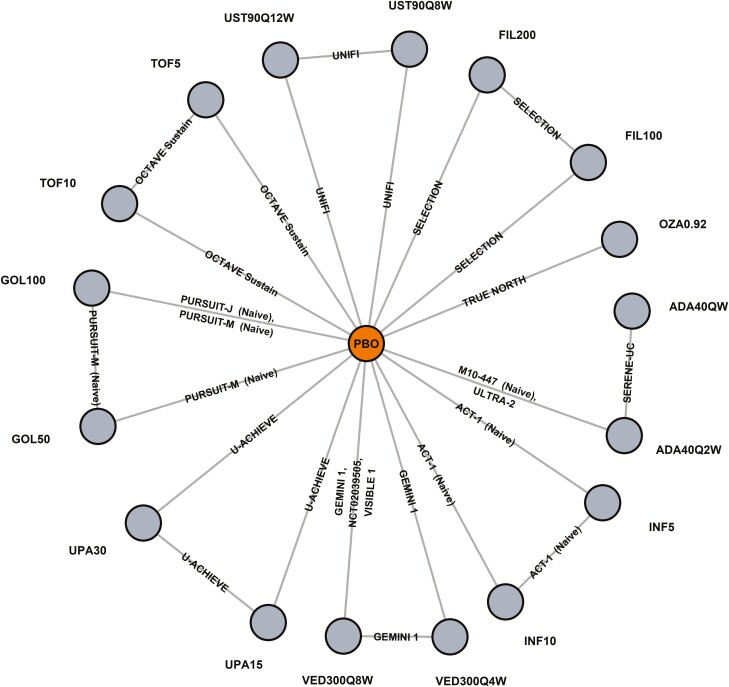
Maintenance network plot.

The SLR search strategy identified 5629 records, of which 3966 proceeded to title/abstract review and 812 to full-text review. Ultimately, 293 records covering 48 original RCTs were included in the SLR (Figure 1). From SLR to NMA, 23 RCTs reported by 62 records were found eligible, of which 18 (39 records; 8823 patients) contributed data to the induction network (Figure 2) and 14 (47 records; 5321 patients) to the maintenance network (Figure 3). A total of 25 RCTs were excluded due to study characteristics (eg, phase 2, open-label, single-arm), outcomes (eg, outside of eligible timeframes, not Mayo score based), and/or interventions (eg, not a biologic/SMD of interest). An overview of the 23 included RCTs and their baseline characteristics by induction and maintenance populations are presented in Appendix 3. The included RCT populations were largely comparable in their baseline characteristics, though some heterogeneity was noted in weight, disease duration, extent of disease, and concomitant medications. A risk of bias assessment showed a low risk of bias for all included RCTs, which were all industry sponsored (Appendix 4).

The outcome contributions and definitions of included RCTs are described in Appendix 5. For induction, 18 and 10 RCTs contributed efficacy data for bio-naive and -exposed populations, respectively, and 14 RCTs contributed safety data for overall populations. For maintenance, 12 and 9 RCTs contributed efficacy data for bio-naive and -exposed populations, respectively, and 14 RCTs contributed safety data for overall populations. The following differences in outcome definitions across the RCTs were observed: location of endoscopic reading (generally local in RCTs for biologics vs central in RCTs for SMDs), Mayo score type (all RCTs reported FMS except for TRUE NORTH which only reported an adapted Mayo score [AMS] that excluded the PGA subscore), treatment duration (6–10 weeks induction with most RCTs at 8 weeks, and 42–54 weeks maintenance), and prior biologic experience (most studies defined experience using exposure with some defining it using failure). Of note, induction safety outcomes were assessed after 10 weeks in 2 RCTs (Japic CTI-060298 at 14 weeks and SELECTION at 11 weeks) but were included to be able to assess INF and FIL (Appendix 5).

Of the 14 included maintenance RCTs, 11 (GEMINI 1, NCT02039505, OCTAVE Sustain, PURSUIT-J, PURSUIT-M, SELECTION, SERENE-UC, TRUE NORTH, U-ACHIEVE, UNIFI, and VISIBLE 1) had RR designs and 3 (ACT-1, M10-447, and ULTRA-2) had TT designs. RR clinical response and remission were imputed for ACT-1 and ULTRA-2; no other imputation was performed due to lack of reported data. For M10-447 and ULTRA-2, maintenance-only safety events were obtained by subtracting induction from overall events; for ACT-1, only overall events were reported and thus were included as is. No adjustment for induction responders was made for maintenance safety, based on the assumption that treatment safety and efficacy are independent, unrelated outcomes. The final data inputs for each NMA are provided in Appendix 6, including a table detailing the TT-to-RR maintenance efficacy imputation.

Noted in Appendix 7, an assessment of heterogeneity in baseline (PBO) risks for each outcome revealed significance (Wald test *P*-value <.05) in the following networks, justifying testing the RD link in sensitivity analysis where baseline risk adjustment failed: induction of clinical response (bio-naive), endoscopic improvement (bio-naive and -exposed), all AEs, and discontinuation due to AEs; maintenance of clinical response (bio-naive and -exposed), clinical remission (bio-naive), endoscopic improvement (bio-naive), all AEs, discontinuation due to AEs, and serious AEs. No evidence of inconsistency was detected by UME models in the relevant networks.

Model specifications and selections are detailed in Appendix 7. Findings of selected models in terms of induction and ITT efficacy in bio-naive and -exposed populations, as well as induction and maintenance safety in overall populations, are subsequently described with statistically significant comparisons as determined by pairwise comparisons on the OR scale explicitly noted. Details of maintenance-only efficacy findings among induction responders are presented in tables and figures.

### Efficacy in Bio-naive Populations

The bio-naive induction network includes 12 treatments, 5080 patients, and 66 possible pairwise comparisons. The maintenance network includes 17 treatments (14 for endoscopic improvement), 2648 patients (2579 for clinical response and 2230 for endoscopic improvement), and 136 possible pairwise comparisons (91 for endoscopic improvement).

#### Clinical response

**Figure 4.**
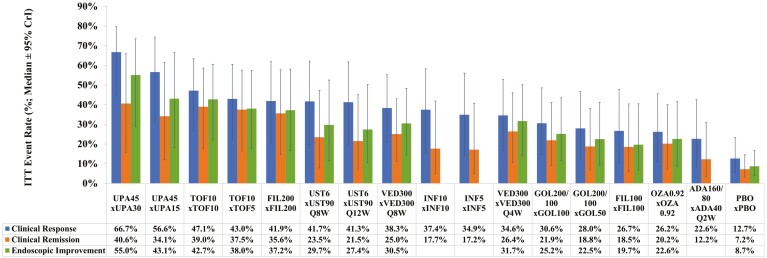
Bio-naive intent-to-treat (ITT) maintenance efficacy adjusted by induction response (absolute rate samples for induction response [per REA] were multiplied by absolute rates samples for each maintenance efficacy [per RE] to obtain ITT rates; median ± 95% CrI rates are presented; treatments are ordered by descending ITT rates for clinical response). Abbreviations: CrI, credible interval; PBO, placebo; RE, random effects model; REA, RE model adjusted for baseline/PBO risk.

All treatments are significantly more efficacious than PBO at inducing clinical response, with UPA45 ranking highest (SUCRA 99%; OR 6.9; absolute rate 79% [95% CrI: 68%–87%]) followed by UST6, INF5, and INF10 (Table 2; Appendix 11, Figure S27). Between active induction treatments, significantly higher efficacies are found for UPA45 versus all other treatments except FIL200 and UST6, and for INF5 versus ADA160/80 (Appendix 8, Figure S6). The median ITT rate of clinical response at the end of maintenance is highest for UPA45 × UPA30 (66.7% [95% CrI: 44.9%–79.7%]), followed by UPA45 × UPA15 (56.6% [95% CrI: 30.5%–74.4%]) and TOF10 × TOF10 (47.1% [95% CrI: 26.9%–63.3%]) (Figure 4; Appendix 11, Figure S26).

**Table 2.** NMA of efficacy outcomes (clinical response, clinical remission, endoscopic improvement) in bio-naive populations^j^.

Phase	Treatment	Clinical response			Clinical remission			Endoscopic improvement		
		OR (vs PBO)	Absolute rate	SUCRA	OR (vs PBO)	Absolute rate	SUCRA	OR (vs PBO)	Absolute rate	SUCRA
Induction (6–10 weeks post-baseline)	Upadacitinib 45 mg QD	6.9^i^	79% (68%–87%)	99%	9.6^i^	50% (25%–77%)	97%	6.9^i^	69% (54%–81%)	99%
	Ustekinumab 6 mg/kg^a^	3.6^i^	67% (50%–80%)	74%	2.0	18% (6%–41%)	37%	1.9^i^	38% (24%–54%)	39%
	Infliximab 5 mg^b^	3.4^i^	65% (53%–76%)	71%	3.9^i^	29% (15%–48%)	74%	3.0^i^	50% (39%–60%)	74%
	Infliximab 10 mg^b^	3.4^i^	65% (51%–77%)	70%	3.2^i^	25% (12%–45%)	61%	3.1^i^	50% (38%–61%)	74%
	Filgotinib 200 mg QD	3.4^i^	65% (47%–79%)	69%	2.3	20% (8%–42%)	44%	2.0^i^	39% (26%–56%)	45%
	Tofacitinib 10 mg BID^c^	3.1^i^	63% (49%–76%)	63%	2.3^i^	19% (8%–41%)	42%	2.1^i^	40% (27%–56%)	46%
	Filgotinib 100 mg QD	2.5^i^	58% (40%–74%)	41%	1.5	13% (5%–32%)	19%	1.4	31% (19%–47%)	19%
	Adalimumab 160/80 mg^d^	2.2^i^	55% (41%–67%)	30%	1.8	16% (7%–31%)	27%	1.6^i^	34% (25%–44%)	26%
	Vedolizumab 300 mg^b^	2.1^i^	54% (39%–69%)	30%	3.3^i^	26% (11%–51%)	62%	2.5^i^	45% (31%–60%)	60%
	Ozanimod 0.92 mg^e^	2.1^i^	54% (37%–69%)	29%	4.1^i^	30% (12%–59%)	73%	3.6^i^	54% (37%–70%)	81%
	Golimumab 200/100 mg^f^	1.9^i^	52% (36%–68%)	24%	3.2^i^	25% (10%–51%)	61%	1.8^i^	37% (25%–51%)	36%
	PBO	1.0	36% (27%–46%)	0%	1.0	9% (5%–17%)	3%	1.0	24% (20%–29%)	2%
Maintenance^k^ (42–54 weeks post-induction response)	Upadacitinib 30 mg QD	10.4^i^	85% (58%–96%)	96%	4.2^i^	52% (20%–82%)	72%	7.2^i^	70% (37%–90%)	86%
	Tofacitinib 10 mg BID^c^	5.6^i^	76% (45%–92%)	82%	6.6^i^	63% (29%–88%)	87%	6.7^i^	69% (36%–89%)	84%
	Vedolizumab 300 mg Q8W	4.7^i^	73% (42%–91%)	76%	3.5^i^	47% (22%–74%)	65%	4.1^i^	58% (28%–82%)	64%
	Upadacitinib 15 mg QD	4.6^i^	72% (39%–91%)	73%	3.0	43% (15%–77%)	57%	3.7^i^	55% (23%–83%)	57%
	Tofacitinib 5 mg BID^c^	4.0^i^	69% (37%–89%)	67%	5.9^i^	60% (27%–86%)	84%	4.8^i^	61% (29%–86%)	69%
	Filgotinib 200 mg QD	3.5^i^	66% (34%–88%)	60%	4.9^i^	56% (24%–84%)	79%	4.3^i^	58% (27%–84%)	65%
	Vedolizumab 300 mg Q4W	3.4^i^	65% (33%–88%)	59%	3.9^i^	50% (20%–79%)	69%	4.6^i^	60% (28%–85%)	68%
	Ustekinumab 90 mg Q8W	3.1^i^	64% (31%–88%)	55%	2.2	36% (12%–69%)	42%	2.5	45% (18%–76%)	39%
	Ustekinumab 90 mg Q12W	3.0^i^	63% (30%–87%)	54%	1.9	33% (11%–66%)	36%	2.2	42% (16%–73%)	31%
	Golimumab 100 mg Q4W^g^	2.7^i^	60% (31%–84%)	47%	2.9^i^	43% (19%–74%)	57%	2.9^i^	49% (24%–77%)	47%
	Infliximab 10 mg/kg Q8W^h^	2.5	58% (25%–85%)	44%	1.5	27% (8%–63%)	26%	NA	NA	NA
	Golimumab 50 mg Q4W^g^	2.2^i^	55% (26%–82%)	36%	2.3	37% (14%–69%)	44%	2.4^i^	44% (19%–74%)	35%
	Infliximab 5 mg/kg Q8W^h^	2.1	54% (22%–83%)	35%	1.4	26% (8%–62%)	24%	NA	NA	NA
	Ozanimod 0.92 mg QD^e^	1.8	50% (22%–78%)	26%	2.5	38% (14%–70%)	47%	2.3	43% (18%–73%)	33%
	Filgotinib 100 mg QD	1.6	47% (19%–77%)	22%	1.9	33% (11%–67%)	36%	1.6	35% (12%–67%)	19%
	Adalimumab 40 mg Q2W^h^	1.3	42% (15%–74%)	15%	1.2	23% (7%–55%)	17%	NA	NA	NA
	PBO	1.0	36% (16%–61%)	4%	1.0	20% (9%–39%)	8%	1.0	25% (12%–44%)	2%

Coloring in SUCRA columns is based on SUCRA value; values of 100% are green in color, values of 0% are red in color, intermediates values are colored along the green-to-red gradient. Abbreviations: AM, adapted Mayo score; BID, twice daily; CrI, credible interval; IV, intravenous; NA, not available; NMA, network meta-analysis; OR, odds ratio; PBO, placebo; Q#W, every # week; QD, once daily; RBS, rectal bleeding score; RE, random effects model; REA, RE model adjusted for baseline/PBO risk; SC, subcutaneous; SFS, stool frequency score; SUCRA, surface under the cumulative ranking curve.

^a^IV dose based on body weight (~6 mg/kg) at week 0.

^b^IV doses at week 0, 2, and 6 for induction.

^c^Tofacitinib studies (OCTAVE 1, OCTAVE 2, and OCTAVE Sustain) additionally required RBS = 0 for clinical remission and for maintenance, bio-naive was defined as non-bio-failure.

^d^SC 160 mg at week 0 and 80 mg at week 2, then 40 mg Q2W.

^e^Oral 0.23 mg QD for 4 days, 0.46 mg QD for 3 days, then 0.92 mg QD starting on day 8. Ozanimod study (TRUE NORTH) used AM to define clinical response (decrease in AM ≥2% and ≥35% from baseline, and a decrease in RBS ≥1 or an absolute RBS ≤1) and remission (SFS ≤1- and ≥1-point decrease from baseline, RBS = 0, and endoscopic subscore ≤1).

^f^SC 200 mg at week 0 and 100 mg at week 2.

^g^Golimumab study PURSUIT-J reported maintenance clinical remission and response as sustained over 2 post-induction timepoints, while study PURSUIT-M reported maintenance clinical response and endoscopic improvement as sustained over 2 post-induction timepoints.

^h^Maintenance outcomes for infliximab and adalimumab from the treat-through studies ACT-1 and ULTRA-2, respectively, were imputed to mimic re-randomized responder design outcomes.

^i^Denotes statistical significance (OR 95% CrI excludes 1). 95% CrIs can be found in Appendices 8 and 9.

^j^Results (medians with 95% CrI as applicable) displayed for ‘best-fitting’ model per fit statistics (REA for induction clinical response, RE for all other outcomes) and ordered in descending (best to worst rank) SUCRA values for clinical response.

^k^Outcomes of maintenance treatment among induction responders.

#### Clinical remission

All treatments except UST6, FIL200, FIL100, and ADA160/80 are significantly more efficacious than PBO at inducing clinical remission, with UPA45 ranking highest (SUCRA 97%; OR 9.6; absolute rate 50% [95% CrI: 25%–77%]) followed by INF5, OZA0.92, and VED300 (Table 2; Appendix 11, Figure S28). Between active induction treatments, significantly higher efficacies are found for UPA45 versus all other treatments except GOL200/100, VED300, INF5, and OZA0.92, and for INF5 versus ADA160/80 (Appendix 8, Figure S8). The median ITT rate of clinical remission at the end of maintenance is highest for UPA45 × UPA30 (40.6% [95% CrI: 15.8%–65.9%]), followed by TOF10 × TOF10 (39.0% [95% CrI: 17.6%–58.6%]) and TOF10 × TOF5 (37.5% [95% CrI: 16.5%–57.6%]) (Figure 4; Appendix 11, Figure S26).

#### Endoscopic improvement

All treatments except FIL100 are significantly more efficacious than PBO at inducing endoscopic improvement, with UPA45 ranking highest (SUCRA 99%; OR 6.9; absolute rate 69% [95% CrI: 54%–81%]) followed by OZA0.92, INF5, and INF10 (Table 2). Between active induction treatments, significantly higher efficacies are found for UPA45 versus all other treatments except OZA0.92, and for OZA0.92, INF10, and INF5 each versus FIL100 and ADA160/80 (Appendix 8, Figure S10). The median ITT rate of endoscopic improvement at the end of maintenance is highest for UPA45 × UPA30 (55.0% [95% CrI: 28.8%–73.5%]), followed by UPA45 × UPA15 (43.1% [95% CrI: 18.3%–66.6%]) and TOF10 × TOF10 (42.7% [95% CrI: 22.1%–60.6%]) (Figure 4; Appendix 11, Figure S26).

The high ITT efficacy rates of UPA45 × UPA30 translated to positive, low NNTs relative to all comparators, many with 95% CrI that exclude zero (Appendix 13, Table S27). For maintenance-only among bio-naive induction responders, UPA30 also exhibits high comparative efficacy, ranking first for clinical response and endoscopic improvement, while TOF10 ranks first for clinical remission (Table 2). The bio-naive clinical response induction and all efficacy ITT logit model findings are numerically consistent with their corresponding RD model findings (Appendix 12).

### Efficacy in Bio-exposed Populations

The bio-exposed induction network includes 9 treatments, 2839 patients (2823 for clinical response and endoscopic improvement), and 36 possible pairwise comparisons. The maintenance network includes 13 treatments (12 for endoscopic improvement), 1405 patients (1348 for clinical response and 1283 for endoscopic improvement), and 78 possible pairwise comparisons (66 for endoscopic improvement).

#### Clinical response

**Figure 5.**
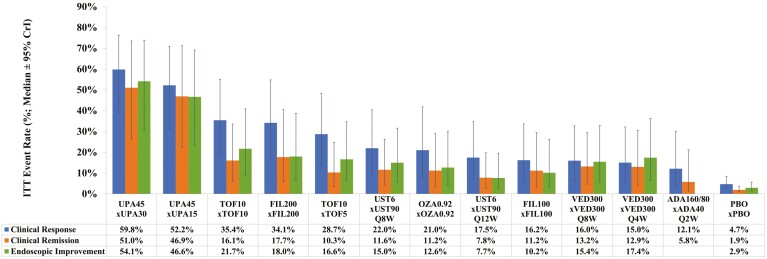
Bio-exposed intent-to-treat (ITT) maintenance efficacy adjusted by induction response (absolute rate samples for induction response [per RE] were multiplied by absolute rates samples for each maintenance efficacy [per RE] to obtain ITT rates; median ± 95% CrI rates are presented; treatments are ordered by descending ITT rates for clinical response). Abbreviations: CrI, credible interval; PBO, placebo; RE, random effects model.

All treatments except VED300 and ADA160/80 are significantly more efficacious than PBO at inducing clinical response, with UPA45 ranking highest (SUCRA 99%; OR 13.6; absolute rate 79% [95% CrI: 60%–90%]) followed by FIL200, TOF10, and UST6 (Table 3; Appendix 11, Figure S27). Between active induction treatments, significantly higher efficacies are found for UPA45 versus all other treatments except FIL200, and for FIL200 versus ADA160/80 and VED300 (Appendix 9, Figure S12). The median ITT rate of clinical response at the end of maintenance is highest for UPA45 × UPA30 (59.8% [95% CrI: 39.0%–76.3%]), followed by UP45 × UPA15 (52.2% [95% CrI: 31.0%–70.9%]) and TOF10 × TOF10 (35.4% [95% CrI: 18.4%–55.1%]) (Figure 5; Appendix 11, Figure S26).

**Table 3.** NMA of efficacy outcomes (clinical response, clinical remission, endoscopic improvement) in bio-exposed populations^h^.

Phase	Treatment	Clinical response			Clinical remission			Endoscopic improvement		
		OR (vs PBO)	Absolute rate	SUCRA	OR (vs PBO)	Absolute rate	SUCRA	OR (vs PBO)	Absolute rate	SUCRA
Induction (6–10 weeks post-baseline)	Upadacitinib 45 mg QD	13.6^g^	79% (60%–90%)	99%	9.8^g^	18% (6%–45%)	97%	15.1^g^	61% (33%–85%)	99%
	Filgotinib 200 mg QD	5.4^g^	59% (35%–80%)	80%	3.3^g^	7% (2%–21%)	47%	2.6^g^	21% (8%–47%)	61%
	Tofacitinib 10 mg BID^a^	3.8^g^	51% (30%–72%)	66%	5.2^g^	10% (3%–29%)	72%	4.8^g^	33% (14%–62%)	80%
	Ustekinumab 6 mg/kg^b^	3.6^g^	49% (26%–72%)	62%	5.9^g^	12% (3%–35%)	77%	3.7^g^	28% (11%–56%)	72%
	Ozanimod 0.92 mg^c^	2.6^g^	42% (19%–69%)	47%	3.5^g^	7% (2%–23%)	50%	1.5	14% (4%–39%)	37%
	Filgotinib 100 mg QD	2.6^g^	42% (21%–66%)	46%	1.9	4% (1%–14%)	21%	1.8	16% (6%–39%)	43%
	Vedolizumab 300 mg^d^	1.6	30% (14%–53%)	24%	3.2^g^	7% (2%–20%)	45%	1.2	11% (4%–26%)	25%
	Adalimumab 160/80 mg^e^	1.4	28% (12%–53%)	21%	2.7	6% (1%–19%)	38%	1.1	10% (4%–26%)	20%
	PBO	1.0	21% (12%–34%)	4%	1.0	2% (1%–6%)	2%	1.0	9% (5%–18%)	12%
Maintenance^i^ (42–54 weeks post–induction response)	Upadacitinib 30 mg QD	12.1^g^	78% (54%–91%)	93%	19.4^g^	66% (35%–90%)	93%	14.6^g^	70% (42%–90%)	93%
	Tofacitinib 10 mg BID^a^	8.7^g^	71% (47%–88%)	84%	4.7^g^	32% (14%–59%)	55%	4.8^g^	44% (21%–71%)	62%
	Upadacitinib 15 mg QD	7.4^g^	68% (42%–86%)	76%	15.4^g^	61% (30%–87%)	87%	9.5^g^	61% (32%–85%)	81%
	Filgotinib 200 mg QD	5.1^g^	59% (34%–80%)	63%	4.4^g^	31% (11%–64%)	52%	2.8^g^	31% (13%–60%)	41%
	Tofacitinib 5 mg BID	4.8^g^	58% (33%–80%)	59%	2.6	21% (8%–45%)	30%	3.1^g^	33% (14%–61%)	43%
	Vedolizumab 300 mg Q8W^d^	4.3^g^	55% (30%–79%)	55%	8.2^g^	45% (21%–75%)	74%	7.1^g^	53% (26%–80%)	73%
	Ozanimod 0.92 mg QD^c^	3.8^g^	52% (28%–76%)	49%	3.8^g^	28% (11%–58%)	46%	2.8^g^	31% (13%–59%)	41%
	Vedolizumab 300 mg Q4W	3.8^g^	52% (24%–79%)	48%	8.0^g^	45% (17%–78%)	72%	9.6^g^	61% (29%–87%)	82%
	Ustekinumab 90 mg Q8W	3.0^g^	46% (24%–69%)	37%	3.2^g^	24% (10%–48%)	39%	2.8^g^	31% (14%–57%)	41%
	Adalimumab 40 mg Q2W^f^	2.9	45% (18%–77%)	37%	2.7	21% (5%–62%)	34%	NA	NA	NA
	Filgotinib 100 mg QD	2.3	40% (19%–66%)	27%	3.8^g^	28% (9%–61%)	45%	2.1	25% (9%–54%)	29%
	Ustekinumab 90 mg Q12W	2.0	37% (18%–61%)	21%	1.9	16% (6%–36%)	20%	1.2	16% (6%–36%)	11%
	PBO	1.0	22% (15%–31%)	1%	1.0	9% (6%–14%)	2%	1.0	14% (9%–22%)	5%

Coloring in SUCRA columns is based on SUCRA value; values of 100% are green in color, values of 0% are red in color, intermediates values are colored along the green-to-red gradient. Abbreviations: AM, adapted Mayo score; BID, twice daily; CrI, credible interval; IV, intravenous; NA, not available; NMA, network meta-analysis; OR, odds ratio; PBO, placebo; Q#W, every # week; QD, once daily; RBS, rectal bleeding score; RE, random effects model; SC, subcutaneous; SFS, stool frequency score; SUCRA, surface under the cumulative ranking curve.

^a^Tofacitinib studies (OCTAVE 1, OCTAVE 2, and OCTAVE Sustain) additionally required RBS=0 for clinical remission and for maintenance, bio-exposed was defined as bio-failed.

^b^IV dose based on body weight (~6 mg/kg) at week 0. Ustekinumab study (UNIFI) defined bio-exposed as bio-failed for clinical remission and endoscopic improvement.

^c^Oral 0.23 mg QD for 4 days, 0.46 mg QD for 3 days, then 0.92 mg QD starting on day 8. Ozanimod study (TRUE NORTH) used AM to define clinical response (decrease in AM ≥2% and ≥35% from baseline, and a decrease in RBS ≥1 or an absolute RBS ≤1) and remission (SFS ≤1- and ≥1-point decrease from baseline, RBS = 0, and endoscopic subscore ≤1).

^d^IV doses at weeks 0, 2, and 6 for induction. Vedolizumab study (GEMINI) defined bio-exposed as bio-failed.

^e^SC 160 mg at week 0 and 80 mg at week 2, then 40 mg Q2W.

^f^Maintenance outcomes for adalimumab from the treat-through study ULTRA-2 were imputed to mimic re-randomized responder design outcomes.

^g^Denotes statistical significance (OR 95% CrI excludes 1). 95% CrIs can be found in Appendices 8 and 9.

^h^Results (medians with 95% CrI as applicable) displayed for ‘best-fitting’ model per fit statistics (RE for all outcomes) and ordered in descending (best to worst rank) SUCRA values for clinical response.

^i^Outcomes of maintenance treatment among induction responders.

#### Clinical remission

All treatments except FIL100 and ADA160/80 are significantly more efficacious than PBO at inducing clinical remission, with UPA45 ranking highest (SUCRA 97%; OR 9.8; absolute rate 18% [95% CrI: 6%–45%]) followed by UST6, TOF10, and OZA0.92 (Table 3; Appendix 11, Figure S28). Between active induction treatments, significantly higher efficacies are found for UPA45 versus all other treatments except TOF10 and UST6, and for TOF10 and UST6 each versus FIL100 (Appendix 9, Figure S14). The median ITT rate of clinical remission at the end of maintenance is highest for UPA45 × UPA30 (51.0% [95% CrI: 26.0%–73.5%], followed by UPA45 × UPA15 (46.9% [95% CrI: 22.3%–71.2%]) and FIL200 × FIL200 (17.7% [95% CrI: 5.8%–40.5%]) (Figure 5; Appendix 11, Figure S26).

#### Endoscopic improvement

UPA45, FIL200, TOF10, and UST6 are significantly more efficacious than PBO at inducing endoscopic improvement, with UPA45 ranking highest (SUCRA 99%; OR 15.1; absolute rate 61% [95% CrI: 33%–85%]) followed by TOF10, UST6, and FIL200 (Table 3). Between active induction treatments, significantly higher efficacies are found for UPA45 versus all other treatments except TOF10, and for TOF10 versus ADA160/80 and VED300 (Appendix 9, Figure S16). The median ITT rate of endoscopic improvement at the end of maintenance is highest for UPA45 × UPA30 (54.1% [95% CrI: 30.8%–73.9%], followed by UPA45 × UPA15 (46.6% [95% CrI: 23.5%–69.1%]) and TOF10 × TOF10 (21.7% [95% CrI: 8.9%–40.9%]) (Figure 5; Appendix 11, Figure S26).

The high ITT efficacy rates of UPA45 × UPA30 translated to positive, low NNTs relative to all comparators, with the associated 95% CrIs excluding 0 (Appendix 13, Table S27). For maintenance-only among bio-exposed induction responders, UPA30 also exhibits high comparative efficacy, ranking first for all outcomes (Table 3). The bio-exposed clinical response and endoscopic improvement induction and efficacy ITT logit model findings are numerically consistent with their corresponding RD model findings (Appendix 12).

### Safety in Overall Populations

The induction network for safety events (eg, all AEs, discontinuation due to AEs, serious AEs, and serious infections) includes 11 treatments (10 treatments for discontinuation due to AEs), 14 studies (13 for discontinuation due to AEs), 7380 patients (6741 for discontinuation due to AEs), and 55 possible pairwise comparisons (45 for discontinuation due to AEs). The maintenance network includes 17 treatments (18 for serious infections), 13 studies (11 serious infections), 4841 patients (4778 for discontinuation due to AEs and 5001 for serious infections), and 136 possible pairwise comparisons (120 for discontinuation due to AEs and 153 for serious infections).

Between induction treatments including PBO, a handful of significant differences in the safety events assessed are observed. For all AEs, GOL200/100 is ranked highest while INF5 is ranked lowest and has significantly higher odds than VED300 and FIL100 (Table 4; Appendix 10, Figure S18). For discontinuation due to AEs, UPA45 is ranked highest and has significantly lower odds than TOF10, ADA160/80, and PBO, while PBO is ranked lowest (Table 4; Appendix 10, Figure S20; Appendix 11, Figure S27), a finding that the RD analysis confirms (Appendix 12). For serious AEs, GOL200/100 and OZA0.92 are ranked highest and lowest, respectively, with no significant difference observed (Table 4; Appendix 10, Figure S22; Appendix 11, Figure S27). Likewise, for serious infections, GOL200/100 and PBO are ranked highest and lowest, respectively, with no significant difference observed (Table 4; Appendix 10, Figure S24).

**Table 4.** NMA of safety outcomes (all AEs, discontinuation due to AEs, serious AEs, serious infections) in overall populations^h^.

Phase	Treatment	All AEs			Discontinuation due to AEs			Serious AEs			Serious Infections		
		OR (vs PBO)	Absolute rate	SUCRA	OR (vs PBO)	Absolute rate	SUCRA	OR (vs PBO)	Absolute rate	SUCRA	OR vs PBO)	Absolute rate	SUCRA
Induction (6–10 weeks post-baseline)	Golimumab 200/100 mg^a^	0.7	43% (30%–57%)	87.8%	0.3	1.1% (0.0%–11.1%)	77.2%	0.4	2.7% (0.9%–7.5%)	79.1%	0.2	0.2% (0.0%–1.5%	82.6%
	Vedolizumab 300 mg^d^	0.7	45% (35%–57%)	80.2%	0.7	2.9% (0.7%–10.2%)	52.0%	0.6	3.9% (1.5%–9.8%)	59.8%	0.3	0.4% (0.0%–2.0%	68.9%
	Ozanimod 0.92 mg^b^	0.7	45% (33%–60%)	77.4%	1.0	4.4% (1.2%–14.8%)	31.0%	1.3	7.8% (2.7%-22.2%)	18.0%	0.8	0.9% (0.2%–4.1%	37.8%
	Filgotinib 100 mg QD	0.9	49% (37%–60%)	65.3%	0.7	3.0% (1.0%–8.9%)	52.0%	1.1	6.7% (2.6%-16.5%)	22.9%	0.9	1.0% (0.2%–4.1%	32.7%
	Adalimumab 160/80 mg^c^	0.9	51% (41%–61%)	51.6%	0.9	3.6% (1.7%–8.0%)	40.9%	0.6	3.8% (1.8%–7.9%)	61.7%	0.9	1.0% (0.2%–3.5%	32.4%
	Filgotinib 200 mg QD	1.0	52% (40%–64%)	44.5%	0.9	3.8% (1.3%–11.1%)	36.6%	0.9	5.8% (2.2%–14.7%)	33.0%	0.5	0.5% (0.1%–2.5%	59.6%
	Ustekinumab 6 mg/kg^e^	1.0	52% (40%–64%)	44.1%	NA	NA	NA	0.5	3.0% (1.1%–8.1%)	74.1%	0.2	0.2% (0.0%–1.6%	80.9%
	PBO	1.0	53% (45%–60%)	39.5%	1.0	4.2% (2.8%–6.2%)	28.8%	1.0	6.2% (3.8%–10.0%)	23.2%	1.0	1.1% (0.5%–2.5%	22.9%
	Tofacitinib 10 mg BID	1.0	54% (43%–64%)	33.4%	1.0	4.1% (1.6%–11.1%)	33.6%	0.6	4.0% (1.7%–9.5%)	57.2%	0.6	0.7% (0.2%–2.6%	47.9%
	Upadacitinib 45 mg QD	1.1	56% (45%–66%)	22.3%	0.2^g^	1.1% (0.4%–2.8%)	90.1%	0.5	3.5% (1.4%–8.4%)	66.9%	0.9	0.9% (0.2%–3.4%	33.2%
	Infliximab 5 mg^d^	2.3	72% (48%–87%)	4.0%	0.6	2.5% (0.6%–10.1%)	58.0%	0.7	4.2% (1.3%–12.1%)	54.1%	0.5	0.6% (0.0%–5.0%	51.2%
Maintenance^i^ (40–54 weeks post–induction response)	Ustekinumab 90 mg Q12W	0.6	53% (30%–76%)	90.4%	0.4	3.2% (1.0%–9.8%)	67.4%	0.7	6.7% (2.3%–6.7%)	57.3%	1.5	2.9% (0.7%–13.4%)	29.9%
	Tofacitinib 5 mg BID	0.9	62% (38%–81%)	73.0%	0.4	3.3% (1.2%–8.9%)	67.6%	0.8	6.8% (2.2%–6.8%)	56.1%	1.0	1.9% (0.2%–17.5%)	45.7%
	Vedolizumab 300 mg Q4W	0.9	63% (37%–83%)	69.6%	0.3	2.6% (0.7%–8.5%)	75.1%	0.6	5.6% (1.9%–5.6%)	67.7%	0.5	0.9% (0.1%–5.7%)	67.2%
	Filgotinib 100 mg QD	0.9	63% (39%–82%)	69.1%	1.9	13.1% (3.7%–37.7%)	11.8%	1.2	10.5% (3.0%–10.5%)	32.1%	1.8	3.4% (0.4%–27.4%)	29.3%
	Ustekinumab 90 mg Q8W	0.9	64% (39%–83%)	66.9%	0.2^g^	1.7% (0.4%–5.8%)	88.5%	0.9	7.6% (2.7%–7.6%)	48.9%	0.7	1.3% (0.2%–7.5%)	57.3%
	PBO	1.0	66% (50%–79%)	63.8%	1.0	7.4% (4.5%–11.7%)	29.1%	1.0	8.7% (6.0%–8.7%)	37.7%	1.0	1.9% (1.2%–3.1%)	44.3%
	Vedolizumab 300 mg Q8W	1.0	67% (45%–83%)	58.0%	0.4^g^	3.0% (1.1%–7.6%)	71.7%	0.7	6.7% (3.0%–6.7%)	57.4%	0.8	1.5% (0.3%–7.0%)	52.2%
	Upadacitinib 15 mg QD	1.1	68% (43%–86%)	53.3%	0.3	2.4% (0.6%–8.2%)	77.8%	0.5	4.4% (1.5%–4.4%)	78.1%	0.8	1.6% (0.3%–6.5%)	51.6%
	Upadacitinib 30 mg QD	1.2	69% (44%–86%)	49.5%	0.5	4.1% (1.2%–12.3%)	57.5%	0.4	3.8% (1.2%–3.8%)	84.1%	0.6	1.2% (0.2%–5.3%)	61.1%
	Filgotinib 200 mg QD	1.2	70% (47%–86%)	46.5%	1.1	8.2% (2.1%–27.3%)	29.1%	1.2	10.5% (3.1%–10.5%)	31.6%	1.0	1.9% (0.2%–18.2%)	46.6%
	Infliximab 5 mg/kg Q8W	1.2	70% (43%–88%)	45.4%	0.9	6.6% (2.0%–20.1%)	36.4%	0.8	7.0% (2.7%–7.0%)	54.9%	0.6	1.1% (0.2%–5.5%)	63.0%
	Tofacitinib 10 mg BID	1.3	71% (47%–87%)	41.5%	0.5	3.5% (1.3%–9.4%)	64.4%	0.8	7.5% (2.5%–7.5%)	49.8%	0.4	0.8% (0.0%–10.8%)	67.0%
	Adalimumab 40 mg Q2W^f^	1.4	73% (54%–86%)	33.5%	1.3	9.5% (3.9%–23.5%)	19.5%	1.7	14.3% (6.5%–14.3%)	12.6%	0.4	0.8% (0.0%–10.5%)	66.5%
	Golimumab 50 mg Q4W	1.6	75% (54%–90%)	27.6%	0.8	5.9% (1.6%–18.6%)	42.2%	0.9	7.9% (2.7%–7.9%)	47.2%	1.8	3.4% (0.7%–17.0%)	27.5%
	Infliximab 10 mg/kg Q8W	1.8	77% (51%–92%)	23.5%	1.0	7.3% (2.2%–21.8%)	32.0%	0.9	7.9% (3.1%–7.9%)	46.3%	1.7	3.3% (0.9%–12.5%)	26.1%
	Ozanimod 0.92 mg QD	1.7	76% (55%–89%)	23.0%	0.5	3.5% (0.6%–15.8%)	62.5%	0.6	5.7% (2.0%–5.7%)	65.7%	0.4	0.9% (0.1%–5.4%)	68.8%
	Golimumab 100 mg Q4W	1.9^g^	79% (60%–91%)	15.5%	1.5	10.4% (3.4%–28.5%)	17.6%	1.4	11.8% (4.4%–11.8%)	22.4%	1.8	3.4% (0.6%–17.1%)	27.1%
	Adalimumab 40 mg QW	NA	NA	NA	NA	NA	NA	NA	NA	NA	0.4	0.7% (0.0%–11.4%)	68.9%

Coloring in SUCRA columns is based on SUCRA value; values of 100% are green in color, values of 0% are red in color, intermediates values are colored along the green-to-red gradient. Abbreviations: AE, adverse event; BID, twice daily; CrI, credible interval; IV, intravenous; NA, not available; NMA, network meta-analysis; OR, odds ratio; PBO, placebo; Q#W, every # week; QD, once daily; RE, random effects model; REA, RE model adjusted for baseline/PBO risk; SC, subcutaneous; SUCRA, surface under the cumulative ranking curve.

^a^SC 200 mg at week 0 and 100 mg at week 2.

^b^IV dose based on body weight (~6 mg/kg) at week 0.

^c^SC 160 mg at week 0 and 80 mg at week 2, then 40 mg Q2W.

^d^IV doses at weeks 0, 2, and 6.

^e^Oral 0.23 mg QD for 4 days, 0.46 mg QD for 3 days, then 0.92 mg QD starting on day 8.

^f^For treat-through trials M10-447 and ULTRA-2 for adalimumab, induction numbers were subtracted from the overall numbers to obtain maintenance numbers.

^g^Denotes statistical significance (OR 95% CrI excludes 1). 95% CrIs can be found in Appendices 8 and 9.

^h^Results (medians with 95% CrI as applicable) displayed for ‘best-fitting’ model per fit statistics (REA for all AEs; RE for all others) and ordered in descending absolute rates for all AEs. Safety endpoints as defined in trials.

^i^Outcomes of maintenance treatment among induction responders.

Between maintenance treatments including PBO, some significant differences in the safety events assessed are likewise observed. For all AEs, UST90Q12W is ranked highest, while GOL100 is ranked lowest and has significantly higher odds than UST90Q12W and PBO (Table 4; Appendix 10, Figure S19). In the RD analysis, OZA0.92 is ranked lowest instead (Appendix 12). For discontinuation due to AEs, UST90Q8W, UPA15, VED300Q4W, and VED300Q8W are ranked first to fourth, respectively, and have significantly lower odds than FIL100; UST90Q8W, UPA15, and VED300Q8W have significantly lower odds than ADA40Q2W; UST90Q8W has significantly lower odds than GOL100; and UST90Q8W and VED400Q8W have significantly lower odds than PBO (Table 4; Appendix 10, Figure S21). For serious AEs, UPA30 is ranked highest and has significantly lower odds than ADA40Q2W, which is ranked lowest (Table 4; Appendix 10, Figure S23). For serious infections, ADA40QW and INF10 are ranked highest and lowest, respectively, with no significant difference observed (Table 4; Appendix 10, Figure S25).

Finally, NNHs of UPA versus comparators for safety events in overall populations are consistent with the above findings, with all estimates being negative (indicating lower risk vs comparators) or high positive values (indicating positive but small RD vs comparators). Furthermore, all NNH estimates that achieved statistical significance were negative (Appendix 13, Table S28).

## Discussion

With a growing number of advanced therapies available for moderately to severely active UC, it is important for clinicians to better understand the relative efficacy and safety of available options. In the absence of direct comparisons gained in head-to-head studies, indirect comparisons conducted through NMAs provide clinicians valuable insights that may aid in their decision-making. In the present study, we confirmed the findings of recent NMAs by Lasa et al^11^ and Burr et al^12^ that UPA 45 mg was the most efficacious induction therapy, significantly so versus most comparators and independent of prior biologic exposure, at inducing clinical response, clinical remission, and endoscopic improvement. For maintenance results based on induction responders only (not per ITT), we confirmed the finding by Lasa et al^11^ that UPA 30 mg had high comparative efficacy, ranking first for all outcomes except for clinical remission in bio-naive populations, where it ranked fourth after TOF10, TOF5, and FIL200.

Going beyond the scope of currently published NMAs, we simulated the absolute ITT efficacies of treatments in an RR maintenance trial by multiplying the MCMC chains of treatments’ induction clinical response rates with each of their maintenance clinical response, clinical remission, and endoscopic improvement rates. The resulting ITT rates show UPA45 induction followed by UPA30 maintenance for responders (UPA45 × UPA30) as most efficacious for all 3 efficacy outcomes in both bio-naive and -exposed populations. Furthermore, UPA45 induction followed by UPA15 maintenance for responders (UPA45 × UPA15) came second for all efficacy outcomes except for clinical remission in bio-naive populations, for which TOF 10 mg induction and maintenance for responders (TOF10 × TOF10) came second. These ITT estimates of efficacy provide a more holistic approach for clinicians to assess and decide the appropriate UC treatment for their patients.

The presentation of ITT efficacy after induction and maintenance is a novel component of this study. We believe this to be a straightforward, interpretable approach to address an inherent limitation of maintenance NMA efficacy results which must be interpreted as contingent on induction response. As a validation of this approach, ITT results can be compared against a TT trial not included in the NMA, namely VARSITY.^31^ The VARSITY study is a TT, head-to-head study of VED versus ADA that was excluded from the NMA as it lacked sufficient data to conduct TT-to-RR efficacy imputation. VARSITY reported the endpoint durable clinical remission, defined as clinical remission at both weeks 14 and 52. This endpoint is akin to ITT clinical remission, albeit in an ITT population defined by a later timepoint induction remission rather than induction response. Despite this difference, similar absolute efficacy can likely be expected for durable clinical remission and ITT clinical remission. Indeed, VARSITY reported the durable clinical remission absolute efficacy of VED and ADA in the overall population to be 18.3% and 11.9%, respectively. These rates observed in the TT trial are between the respective ITT clinical remission rates estimated in the NMA in bio-naive (25.0% and 12.2%, respectively) and bio-exposed (13.2% and 5.8%, respectively) populations.^31^ In all, this provides external validity for the ITT methodology, and at a minimum for the ITT clinical remission of VED and ADA.

Regarding safety, the present NMA observed only a handful of significant differences between treatments and PBO for the 4 safety events assessed (ie, all AEs, discontinuation due to AEs, serious AEs, serious infections) during induction and maintenance. Discontinuation rates are important to consider because they may signal a balance between drug efficacy and drug safety. Specifically, relative to PBO, UPA45 was significantly better at avoiding discontinuation due to AEs during induction, GOL100 was significantly worse at avoiding all AEs during maintenance, and both UST90Q8W and VED300Q8W were significantly better at avoiding discontinuation due to AEs during maintenance. No significant difference between all treatments versus PBO was observed for serious infections and serious AEs. Lasa et al^11^ likewise observed insignificant and minimal differences between treatments for all and serious AEs during induction, respectively. No published NMA to date has assessed treatment safety during maintenance. Together, these observations suggest that the safety profiles of currently approved biologics and SMDs for moderately to severely active UC are generally comparable. However, the NMA methodology may be limited at identifying significant differences in safety outcomes given that rates of safety events, particularly serious ones, observed in phase 3 RCTs are considerably lower relative to efficacy outcomes.^23^

Our study included RCTs evaluating INF, ADA, GOL, VED, UST, TOF, FIL, OZA, and UPA. Deviating from Lasa et al^11^ and Burr et al,^12^ we did not consider etrolizumab nor the subcutaneous (SC) formulation of VED and INF maintenance to be relevant interventions but included all approved maintenance doses of TOF (5 or 10 mg BID), intravenous (IV) VED (every 4 or 8 weeks), UST (every 8 or 12 weeks), ADA (every 1 or 2 weeks), GOL (50 or 100 mg), and IV INF (5 or 10 mg/kg) as separate nodes in the networks. After imposing duration limits for induction (6–10 weeks) and maintenance (at least 40 weeks) to minimize outcome heterogeneity, which Lasa et al^11^ and Burr et al^12^ did not account for, we synthesized efficacy and safety evidence from 23 phase 3 RCTs. Even with these differences in the evidence base, recent NMAs—those conducted in 2021 or later—generally show similar distributions of treatment rankings.^11,12^ An earlier published NMA that excluded the more recent therapies (ie, FIL, OZA, and UPA) found INF to be most efficacious in bio-naive populations, and UST and TOF to be most efficacious in bio-exposed populations.^32^

In contrast to recently published NMAs of maintenance treatments in moderately to severely active UC,^11,32^ instead of conducting separate NMAs for RR and TT maintenance trials, we combined them in the same NMAs using the TT-to-RR efficacy imputation approved and used by the ERG for TA633.^20^ In every network, we addressed concerns of heterogenous treatment carryover effects and/or baseline risks by (1) assessing the significance of baseline (PBO) risk heterogeneity; (2) testing the selected model with baseline risk adjustment; and (3) if baseline risk heterogeneity was significant but adjustment did not converge or run, testing the RD model assuming FE. Ultimately, baseline risk adjustment was successful and selected in 2 networks (ie, induction clinical response in bio-naive populations and all AEs) and of the 7 RD models tested, none substantially changed the original conclusions.

The present study has several limitations besides the usual limitations of NMAs (ie, dependence on assumptions of transitivity and consistency, susceptibility to the methodological quality of included RCTs).^33,34^ First, there are important sources of heterogeneity across the included RCTs. For example, endoscopic readings were generally performed locally in older RCTs of biologic agents and centrally in newer RCTs of SMDs, potentially resulting in higher PBO rates for biologics.^35^ However, baseline risk adjustments in the present study would have mitigated much of this heterogeneity. Another source of heterogeneity is the use of AMS rather than FMS in the RCTs for UPA and OZA to re-randomize patients and/or define treatment efficacy. In the present study, FMS outcomes were obtained for UPA from ad hoc analyses of its patient-level data, which were not available for Lasa et al^11^ and Burr et al.^12^ Given that results were directionally similar across the present study, Lasa et al,^11^ and Burr et al,^12^ a large difference in AMS outcomes for OZA would not be expected. An additional limitation of the present study is related to the imputation of TT-to-RR maintenance efficacy, which introduced assumptions (eg, number of responders at end of induction is a proxy for the total number of patients entering maintenance) into the NMA dataset. However, similar assumptions were likewise used in the NICE assessment of UST for UC.^20^ Additionally, evidence produced by this NMA, which sourced treatment efficacy and safety data from industry-sponsored RCTs, should be weighed in the context of real-world data when available. Incorporation of any future academic studies, especially studies utilizing head-to-head trial design, would likely improve the generalizability of the NMA. Finally, as noted by Lasa et al,^11^ several newer biologics currently in phase 2 of clinical development, especially anti-IL-23 drugs (ie, risankizumab, mirikizumab, guselkumab, and brazikumab), are also expected to yield phase 3 RCT results, so another update of this NMA may soon be necessary.

## Conclusion

The present study suggests that UPA 45 mg induction and 30 mg maintenance may be overall highest performing advanced treatment at inducing and maintaining clinical response, clinical remission, and endoscopic improvement in patients with moderately to severely active UC, regardless of prior biologic exposure. It also suggests that the safety profiles of currently approved advanced treatments, to the extent that they can be indirectly assessed with NMA, are generally consistent. As with any indirect comparison, the results presented in the present study should be interpreted with caution and in the context of a patient’s individual needs. However, given the limited evidence from head-to-head trials, these results can help clinicians better understand the growing repertoire of advanced treatments for moderately to severely active UC.

## Supplementary Material

otad009_suppl_Supplementary_Material

## Data Availability

The authors confirm that the data supporting the findings of this study are available within the article and its supplementary materials.

## Conference Presentation

The 17th Congress of European Crohn’s and Colitis Organisation, Vienna, Austria, February 16–19, 2022.
