# Supplementary material for: Efficacy and Safety of Advanced Therapies for Moderately to Severely Active Ulcerative Colitis at Induction and Maintenance: An Indirect Treatment Comparison Using Bayesian Network Meta-analysis
Source: Crohns Colitis 360. 2023 Mar 1;5(2):otad009. doi: 10.1093/crocol/otad009 (PMC10045885; doi:10.1093/crocol/otad009)
Supplement: otad009_suppl_Supplementary_Material [file otad009_suppl_supplementary_material.docx]

# Supplementary Data

## Appendix 1: Search strategy

An SLR was performed on January 6, 2022 to identify all relevant studies related to the clinical efficacy and safety of UPA and other treatments for adult patients with moderate to severe active UC. The SLR was conducted as per guidance from the Cochrane Handbook for Systematic Reviews of Interventions, Centre for Reviews and Dissemination (CRD)’s Guidance for Undertaking Reviews in Healthcare, and Methods for the Development of NICE Public Health Guidance. Using the Ovid platform, searches for English-language publications of RCTs reporting the clinical efficacy and/or safety of UPA and other approved treatments were conducted on January 6, 2022 (from inception of the databases) in the following databases:

- Medical Literature Analysis and Retrieval System Online [MEDLINE®] through Ovid MEDLINE(R)
- MEDLINE® Epub Ahead of Print, In-Process & Other Non-Indexed Citations
- Excerpta Medica Database [Embase®]
- Cochrane Database of Systematic Reviews (CDSR)
- The Database of Abstracts of Reviews of Effects (DARE) database (via Cochrane Library)
- The Cochrane Central Register of Controlled Trials (CENTRAL) database
- The Health Technology Assessment (HTA) database (via Cochrane Library)

Search strategies for the above databases are presented in **Table 5**. Disease search terms were comprised of subject headings and synonyms for ulcerative colitis. Intervention search terms were developed using subject headings, drug names, and their brand or code names. Lastly, search terms from previously published SLRs and NICE guidance were used to comprehensively capture RCT search terms.^36^

In addition to the database searches, keyword searches of the annual proceedings of scientific meetings and clinical trial registers were also conducted for additional available data. The ClinicalTrials.gov website and the International Clinical Trials Registry Platform (ICTRP) were searched to identify any studies not captured in the initial search and to obtain information on RCTs, in the absence of published evidence. “Ulcerative colitis” and the name of interventions were used as search terms. The ClinicalTrials.gov website was also searched to retrieve a list of relevant ongoing and completed trials. Abstracts from relevant conference proceedings were searched for information available from last 4 years (searched in January 2022) while there was no date limit for clinical trial registers. The following organizations were searched for conferences:

- Digestive Disease Week (DDW)
- Crohn's & Colitis Congress (CCC)
- International Society for Pharmacoeconomics and Outcomes (ISPOR) - The Professional Society for Health Economics and Outcomes Research
- United European Gastroenterology Week congress (UEGW)
- American College of Gastroenterology congress (ACG)
- European Crohn’s and Colitis Organization congress (ECCO)

The following websites of the regulatory and HTA authorities in the countries that represent larger reimbursement markets in Europe and North America and provide the most robust resources for the identification of relevant documents of interest were consulted. The countries include the UK (England and Scotland), Canada, France, Australia, and Germany:

- NICE - National Institute for Health and Care Excellence
- SMC - Scottish Medicines Consortium
- PBAC - Pharmaceutical Benefits Advisory Committee
- CADTH - Canadian Agency for Drugs and Technologies in Health
- HAS - Haute Autorité de Santé
- G-BA – Gemeinsamer Bundesausschuss

The search engines of HTA websites have limited options and customisability. Therefore, only the terms “ulcerative colitis” and the name of interventions were entered in the corresponding language. When these terms did not yield results, the HTA websites were searched for different drug names and brands specific in the inclusion criteria of this SLR.

Finally, the bibliographies of SLRs and meta-analyses identified through database searches were used to identify key studies. Bibliographies from selected key studies were also reviewed to ensure literature saturation.

Table 5. Search strategy for clinical evidence

| Search conducted | 06/Jan/2022 | |
| --- | --- | --- |
| Databases searched | EBM Reviews - Cochrane Database of Systematic Reviews <2005 to January 5, 2022> EBM Reviews - ACP Journal Club <1991 to December 2021> EBM Reviews - Database of Abstracts of Reviews of Effects <1st Quarter 2016> EBM Reviews - Cochrane Clinical Answers <December 2021> EBM Reviews - Cochrane Central Register of Controlled Trials <December 2021> EBM Reviews - Cochrane Methodology Register <3rd Quarter 2012> EBM Reviews - Health Technology Assessment <4th Quarter 2016> EBM Reviews - NHS Economic Evaluation Database <1st Quarter 2016> Econlit <1886 to December 30, 2021> Embase <1974 to 2022 January 05> Ovid MEDLINE(R) ALL <1946 to January 05, 2022> | |
| **#** | **Term** | **Hits** |
| 1 | Colitis, Ulcerative/ | 74099 |
| 2 | ulcerative colitis/ | 119784 |
| 3 | (ulcerative colitis or Idiopathic Proctocolitis or Colitis Gravis or colitis ulcerosa or colitis ulcerativa or mucosal colitis or ulcerative colorectitis or ulcerative proctocolitis or ulcerous colitis).ti,ab. | 115932 |
| 4 | or/1-3 | 146006 |
| 5 | Ustekinumab/ or Adalimumab/ or Infliximab/ | 91520 |
| 6 | upadacitinib/ or vedolizumab/ or ustekinumab/ or adalimumab/ or infliximab/ or tofacitinib/ or ozanimod/ or filgotinib/ or mirikizumab/ or golimumab/ or etrasimod/ | 100427 |
| 7 | (upadacitinib or vedolizumab or ustekinumab or adalimumab$ or infliximab$ or tofacitinib or tasocitinib or ozanimod or filgotinib or mirikizumab or golimumab or etrasimod).ti,ab. | 80200 |
| 8 | (RINVOQ$ or ENTYVIO$ or STELARA$ or HUMIRA$ or REMICADE$ or XELJANZ$ or ZEPOSIA$ or RPC1063 or Jyseleca$ or GLPG0634 or GS-6034 or LY3074828 or SIMPONI$ or APD334).ti,ab. | 3688 |
| 9 | or/5-8 | 118961 |
| 10 | 4 and 9 | 19337 |
| 11 | exp Randomized Controlled Trial/ or exp Random Allocation/ or exp randomization/ | 1427806 |
| 12 | exp Placebos/ | 438822 |
| 13 | exp Double-Blind Method/ or exp Single-Blind Method/ | 601359 |
| 14 | exp clinical trial/ or exp clinical trial, phase ii/ or exp clinical trial, phase iii/ or exp controlled clinical trial/ | 2583020 |
| 15 | exp controlled clinical trials as topic/ or exp Randomized Controlled Trials as Topic/ or exp clinical trials as topic/ | 788942 |
| 16 | exp Multicenter Study/ | 622776 |
| 17 | exp Randomized Controlled Trial/ or exp Random Allocation/ or exp randomization/ | 1427806 |
| 18 | exp placebo/ | 375446 |
| 19 | exp double blind procedure/ or exp single blind procedure/ or exp crossover procedure/ | 277382 |
| 20 | exp clinical trial/ or exp phase 2 clinical trial/ or exp phase 3 clinical trial/ or exp controlled clinical trial/ | 2583020 |
| 21 | exp "controlled clinical trial (topic)"/ or exp "clinical trial (topic)"/ or exp "randomized controlled trial (topic)"/ | 377499 |
| 22 | exp multicenter Study/ | 622776 |
| 23 | randomized controlled trial.pt. | 1098261 |
| 24 | controlled clinical trial.pt. | 187512 |
| 25 | random$.ti,ab,kw. | 4257026 |
| 26 | blind$.ti,ab,kw. | 1170808 |
| 27 | (placebo$ or assign* or allocat* or volunteer*).ti,ab,kw. | 2687654 |
| 28 | (parallel$ or factorial$ or crossover* or cross over*).ti,ab,kw. | 1186871 |
| 29 | trial.ti. | 976766 |
| 30 | ('phase 3' or 'phase 2' or 'phase III' or 'phase II').af. | 604207 |
| 31 | ((single or double or triple) adj3 (blind* or mask* or dummy)).af. | 1021953 |
| 32 | ('double-blind' or 'double-blinded').af. | 855768 |
| 33 | (open label or open-label).af. | 223912 |
| 34 | or/11-33 | 9032144 |
| 35 | 10 and 34 | 7586 |
| 36 | (addresses or bibliography or biography or case report or comment or congresses or consensus development conference or duplicate publication or editorial or guideline or in vitro or interview or lectures or letter or monograph or news or "newspaper article" or practice guideline or "review" or "review literature" or "review of reported cases" or review, academic or review, multicase or review, tutorial or twin study).pt. | 10137418 |
| 37 | (animals/ not (humans/ and animals/)) or (animal/ not (human/ and animal/)) | 6083948 |
| 38 | case report/ or case reports/ | 4929319 |
| 39 | or/36-38 | 20101412 |
| 40 | 35 not 39 | 5629 |
| 41 | limit 40 to English | 5298 |
| 42 | limit 41 to human | 5100 |
| 43 | Deduplicate | 3966 |

## Appendix 2: Study selection

The title/abstract screening was conducted by two blinded, independent researchers in parallel using the pre-defined Patient, Intervention, Comparator, Outcomes, and Study Design (PICOS) criteria presented in **Table 6**. Discrepancies between researchers were resolved by a third independent researcher. The same process was followed at full text screening. Data from included studies were extracted into a pre-defined Excel-based template by a single analyst and all results were 100% quality checked (QC’d) by a research associate.

Table 6. Eligibility criteria for clinical evidence

| Element | Inclusion | Exclusion |
| --- | --- | --- |
| Patient population | - Adults (≥16 years old) with moderate-to-severe active UC, regardless of prior biologics exposure or failure | - Non-human - Non-active UC - Paediatric (<16 years old) |
| Intervention and Comparators | - Upadacitinib, vedolizumab, ustekinumab, adalimumab, infliximab, ozanimod, filgotinib, mirikizumab, golilumab, tofacitinib, etrasimod | - Studies not including any interventions of interest |
| Outcomes measures | - Clinical remission per total Mayo score, partial Mayo score, adapted Mayo score, adapted partial Mayo score - Clinical response per total Mayo score, partial Mayo score, adapted Mayo score, adapted partial Mayo score - Deep remission - Steroid-free remission, steroid-free response - Time to response - Endoscopic remission, endoscopic response - Mucosal healing defined by both endoscopy and histology - Histologic response - Sustained clinical remission or clinical response (maintenance phase only) - Changes in Mayo score - Percent of patients with rectal bleeding score, stool frequency score, or physician global assessments of 0-1 - Changes in CRP or FCP - Antidrug antibodies level or serum trough level - Symptoms (bowel urgency or abdominal pain - Safety data (AEs, SAEs, discontinuation due to AEs, death) - Rates of infections or serious infections - Rates of individual AEs - Changes in IBDQ score - IBDQ responders - Any HRQoL including but not limited to: EQ-5D, SF-36, WPAI - Hospitalization or surgery rates - Other relevant efficacy and safety outcomes may be considered | - Non-clinical outcomes - Pharmacokinetic/pharmacodynamic outcomes |
| Study design | - Randomised-controlled clinical trials (Phase 2, Phase 3, or Phase 4), including crossover studies, long term/extension open-label studies, and head-to-head comparison studies - RCT sub-studies if they report an additional outcome of interest or long-term follow-up data - Systematic reviews and meta-analyses (to check the bibliography for relevant RCTs) - Data from CSRs from UPA Phase 3 studies | - Non-human/pre-clinical studies - Reviews/Editorials - Non-randomised and single arm studies - Notes/Comments/Letters - Retrospective studies - Observational studies - Uncontrolled studies - Phase I trials - Studies with fewer than 10 patients per arm - Case series - Case reports |
| Restrictions | - English language | - Non-English studies |

**Abbreviations**: AE=adverse event; CRP=c-reactive protein; CSR=clinical study report; EQ-5D=euroqol-5 dimension; FCP=fecal calprotectin; HRQoL=health related quality of life; IBDQ=inflammatory bowel disease questionnaire; RCT=randomised controlled trial; SAE=serious adverse event; SF-36=36 item short form survey; UC=ulcerative colitis; UPA=upadacitinib; WPAI=work productivity and activity impairment.

## Appendix 3: Included RCTs and characteristics

Table 7. Overview of the twenty-three (23) RCTs included from the clinical SLR

| **Study** | **Phase** | **UC severity** | **Bio-experience** | **Asian study population** | **Induction Phase** | | | **Maintenance Phase** | | | | | | **Primary (bolded) and secondary publication(s)** |
| --- | --- | --- | --- | --- | --- | --- | --- | --- | --- | --- | --- | --- | --- | --- |
|  |  |  |  |  | **Duration (weeks)** | **Total N** | **Included regimen(s) (+PBO)** | **RCT design** | **Induction treatment** | **Induction status** | **Duration (weeks)** | **Total N** | **Included regimen(s) (+PBO)** |  |
| ACT-1 (NCT00036439) | 3 | FM6/12; EMS2 | Naïve |  | 8 | 364 | INF10  INF5 | TT | INF10  INF5  PBO | All | 46 | 364 | INF10  INF5 | **Rutgeerts 2005**^37^ |
| ACT-2 (NCT00096655) | 3 | FM6/12; EMS2 | Naïve |  | 8 | 364 | INF10  INF5 | *Excluded: Duration <40 weeks* | | | | | |  |
| GEMINI 1 (NCT00783718) | 3 | FM6/12; EMS2 | Mixed |  | 6 | 374 | VED300 | RR | VED300 | FM response | 46 | 373 | VED300Q8W  VED300Q4W | **Feagan 2013**^38^**;** Sandborn 2019^39^; Feagan 2017^40^ |
| Japic CTI-060298 | 3 | FM6/12; EMS2 | Naïve | X | 8 | 208 | INF5 | *Excluded: Duration <40 weeks* | | | | | | **Kobayashi 2016**^41^ |
| Jiang 2015 | NR | FM6/12; EMS2 | Naïve | X | 8 | 123 | INF5 (INF3.5 excluded) | *Excluded: Duration <40 weeks* | | | | | | **Jiang 2015**^42^ |
| M10-447 (NCT00853099) | 2/3 | FM6/12; EMS2 | Naïve | X | 8 | 274 | ADA160/80 (ADA80/40 excluded) | TT | ADA160/80  ADA80/40 PBO | All | 44 | 274 | ADA40Q2W (ADA160/80 and ADA80/40 combined) | **Suzuki 2014**^43^ |
| NCT01551290 | 3 | FM6/12; EMS2 | Naïve | X | 8 | 99 | INF5 | *Excluded: Duration <40 weeks* | | | | | | **REMICADEUCO3001 CSR**^44^ |
| NCT02039505 | 3 | FM6/12; EMS2 | Mixed | X | 10 | 246 | VED300 | RR | VED300 | FM response | 50 | 83 | VED300Q8W | **Motoya 2019**^45^**;** Nagahori 2021^46^ |
| OCTAVE 1 (NCT01465763) | 3 | FM6/12; EMS2; RBS1 | Mixed |  | 8 | 598 | TOF10 | *Maintenance in OCTAVE Sustain* | | | | | | **Sandborn 2017**^47^**;** Lichtenstein 2019^48^; Dubinsky 2017^49^; Sandborn 2020^50^; Sandborn 2021^51^; D’Haens 2016^52^; Hanauer 2019^53^; Chiorean 2018^54^; Danese 2017^55^; Sands 2021^56^; Vavricka 2021^57^; Reinisch 2019^58^; Feagan 2017^59^; Lichtenstein 2019^60^; Hudesman 2021^61^ |
| OCTAVE 2 (NCT01458951) | 3 | FM6/12; EMS2; RBS1 | Mixed |  | 8 | 541 | TOF10 | *Maintenance in OCTAVE Sustain* | | | | | |  |
| OCTAVE Sustain (NCT01458574) | 3 | FM6/12; EMS2; RBS1 | Mixed |  | *Induction in OCTAVE 1 and OCTAVE 2* | | | RR | TOF10  TOF15  PBO | FM response | 52 | 593 | TOF10  TOF5 |  |
| PURSUIT-J (NCT01863771) | 3 | FM6/12; EMS2 | Naïve | X | *Excluded: Open-label* | | | RR | GOL200/100 | FM response | 54 | 63 | GOL100 | **Hibi 2017**^62^ |
| PURSUIT-M (NCT00488631) | 3 | FM6/12; EMS2 | Naïve |  | *Induction in PURSUIT-SC* | | | RR | GOL400/200 GOL200/100 | FM response | 54 | 464 | GOL100  GOL50 | **Sandborn 2014**^64^ |
| PURSUIT-SC (NCT00487539) | 3 | FM6/12; EMS2 | Naïve |  | 6 | 774 | GOL200/100 (GOL400/200 excluded) | *Maintenance in PURSUIT-M* | | | | | | **Sandborn 2014**^65^ |
| SELECTION (NCT02914522) | 2b/3 | FM6/12; EMS2; RBS1; SFS1; PGA2 | Mixed |  | 10 | 1348 | FIL200  FIL100 | RR | FIL200 FIL100 | FM response/ AM2 remission | 47 | 571 | FIL200  FIL100 | **Feagan 2021**^66^**;** Loftus 2021^67^; Peyrin-Biroulet 2021^68^; Schreiber 2021^69^; Vermeire 2021^70^; Feagan 2021^71^; Peyrin-Biroulet 2021^72^; Peyrin-Biroulet 2021^73^ |
| SERENE-UC (NCT02065622) | 3 | FM6/12; EMS2 | Mixed |  | *Excluded: Intervention ADA HIR* | | | RR | ADA HIR ADA160/80 | All (efficacy evaluated in FM responders) | 44 | 371 | ADA40Q2W  ADA40QW (ADA TDM excluded; no PBO) | **Panes 2019**^74^; Colombel 2020^75^ |
| TRUE NORTH (NCT02435992) | 3 | FM6/12 | Mixed |  | 10 | 645 | OZA0.92 | RR | OZA0.92 | FM response | 42 | 457 | OZA0.92 | **Sandborn 2021**^76^; Sandborn 2020^77^; Silvio 2020^78^; Subrata 2020^79^; Schreiber 2021^63^; Sands 2021^80^; Osterman 2021^81^ |
| U-ACCOMPLISH  (Study M14-675; NCT03653026) | 3 | AFM5/9; EMS2 | Mixed |  | 8 | 522 | UPA45 | *Induction in U-ACHIEVE Study 3* | | | | | | **CSR Tables**^82^ |
| U-ACHIEVE Study 2 & 3  (Study M14-234; NCT02819635) | 3 | AFM5/9; EMS2 | Mixed |  | 8 | 474 | UPA45 | RR | UPA45 | AM response | 52 | 451 | UPA30  UPA15 | **CSR Tables**^83^ |
| ULTRA-1 (NCT00385736) | 3 | FM6/12; EMS2 | Naïve |  | 8 | 390 | ADA160/80 (ADA80/40 excluded) | *No maintenance* | | | | | | **Reinisch 2011**^84^ |
| ULTRA-2 (NCT00408629) | 3 | FM6/12; EMS2 | Mixed |  | 8 | 518 | ADA160/80 | TT | ADA160/80  PBO | All | 44 | 518 | ADA40Q2W | **Sandborn 2012**^85^**;** Ghosh 2013^86^; Colombel 2013^87^; Sandborn 2011^88^; D’Haens 2012^89^; Sandborn 2013^90^; Panaccione 2015^91^ |
| UNIFI (NCT02407236) | 3 | FM6/12; EMS2 | Mixed |  | 8 | 961 | UST6 (UST130 excluded) | RR | UST130 UST6 | FM response | 44 | 523 | UST90Q12W UST90Q8W | **Sands 2019**^92^**;** Van Assche 2019^93^; Sands 2019^94^; Alcala 2020^95^; Danese 2019^96^; Panaccione 2019^97^ |
| VISIBLE 1 (NCT02611830) | 3 | FM6/12; EMS2 | Mixed |  | *Excluded: Open-label* | | | RR | VED300 | FM response | 46 | 216 | VED300Q8W (VED108Q2W SC excluded) | **Sandborn 2020**^98^ |

**Abbreviations**: AMS=Adapted Mayo score; AM5/9=AMS 5 to 9; AM response=decrease in AMS ≥2 points and ≥30% from baseline, and a decrease in RBS ≥1 or an absolute RBS ≤1; AM2 remission=SFS≤1 and ≥1-point decrease from baseline, RBS=0, and EMS≤1; EMS=endoscopic Mayo subscore; EMS2=EMS≥2; FMS=Full Mayo score; FM6/12=FMS 6 to 12; FM response=decrease in FMS ≥3 points and ≥30% from baseline, and a decrease in RBS ≥1 or an absolute RBS ≤1; HIR=higher induction dosing regimen; N=number of patients randomised; NR=not reported; PBO=placebo; PGA=Physician’s global assessment subscore; PGA2=PGA≥2; RBS=Rectal bleeding subscore; RBS1=RBS≥1; RCT=randomised clinical trial; RR=re-randomised responder; SFS=Stool frequency subscore; SFS1=SFS≥1; TDM=therapeutic drug monitoring; TT=treat-through; UC=ulcerative colitis; X=applicable.

Table 8. Baseline patient characteristics data of induction populations, by biologic exposure subgroup if available

| **Study (biologic exposure subgroup)** | **Treatment arm** | **N** | **Age (years; mean)** | **Age (years; SE)** | **Male (%)** | **Weight (kg; mean)** | **Weight (kg; SE)** | **Disease duration (years; mean)** | **Disease duration (years; SE)** | **Extensive colitis or pancolitis (%)** | **Total Mayo score (mean)** | **Total Mayo score (SE)** | **CRP (mean)** | **CRP (SE)** | **Concurrent immuno-modulators (%)** | **Concurrent steroids (%)** |
| --- | --- | --- | --- | --- | --- | --- | --- | --- | --- | --- | --- | --- | --- | --- | --- | --- |
| ACT-1 (Naive) | INF10 | 122 | 41.8 | 1.3 | 59.0% | 76.9 | 1.5 | 8.4 | 0.7 | 44.3% | 8.4 | 0.1 | 16.0 | 2.1 | 48.4% | 59.8% |
| ACT-1 (Naive) | INF5 | 121 | 42.4 | 1.3 | 64.5% | 80.0 | 1.6 | 5.9 | 0.5 | 46.3% | 8.5 | 0.2 | 14.0 | 1.7 | 54.5% | 57.9% |
| ACT-1 (Naive) | PBO | 121 | 41.4 | 1.2 | 59.5% | 76.8 | 1.5 | 6.2 | 0.5 | 44.6% | 8.4 | 0.2 | 17.0 | 2.5 | 43.8% | 65.3% |
| ACT-2 (Naive) | INF10 | 120 | 40.3 | 1.2 | 56.7% | 79.6 | 1.9 | 6.5 | 0.5 | 37.5% | 8.3 | 0.1 | 14.0 | 2.0 | 41.7% | 55.0% |
| ACT-2 (Naive) | INF5 | 121 | 40.5 | 1.2 | 62.8% | 78.4 | 1.6 | 6.7 | 0.5 | 39.7% | 8.3 | 0.1 | 13.0 | 2.1 | 43.0% | 49.6% |
| ACT-2 (Naive) | PBO | 123 | 39.3 | 1.2 | 57.7% | 76.1 | 1.6 | 6.5 | 0.6 | 40.7% | 8.5 | 0.1 | 16.0 | 2.6 | 43.9% | 48.8% |
| GEMINI 1 (Naive) | VED300 | 130 | 39.7 | 1.1 | 53.1% | 69.2 | 1.5 | 5.8 | 0.5 | 38.5% | 8.4 | 0.2 | NA | NA | 42.3% | 56.2% |
| GEMINI 1 (Naive) | PBO | 76 | 40.5 | 1.3 | 61.8% | 70.0 | 2.2 | 6.1 | 0.7 | 40.8% | 8.5 | 0.2 | NA | NA | 34.2% | 57.9% |
| GEMINI 1 (Exposed/IR) | VED300 | 82 | 39.7 | 1.4 | 61.0% | 74.9 | 1.9 | 6.4 | 0.6 | 64.6% | 8.7 | 0.2 | NA | NA | 22.0% | 52.4% |
| GEMINI 1 (Exposed/IR) | PBO | 63 | 41.8 | 1.7 | 55.6% | 74.2 | 2.1 | 8.0 | 1.0 | 55.6% | 8.6 | 0.2 | NA | NA | 22.2% | 55.6% |
| Japic CTI-060298 (Naive) | INF5 | 104 | 40.0 | 1.2 | 63.5% | 57.6 | 1.2 | 8.1 | 0.7 | 79.8% | 8.6 | 0.1 | 10.0 | 1.5 | 48.1% | 65.4% |
| Japic CTI-060298 (Naive) | PBO | 104 | 37.8 | 1.3 | 64.4% | 60.3 | 1.1 | 7.1 | 0.6 | 80.8% | 8.5 | 0.1 | 7.0 | 1.1 | 47.1% | 66.3% |
| Jiang 2015 (Naive) | INF5 | 41 | 34.3 | 2.2 | 63.4% | 62.8 | 2.3 | 4.4 | 0.4 | 61.0% | NA | NA | 35.8 | 3.5 | 29.3% | 53.7% |
| Jiang 2015 (Naive) | PBO | 41 | 34.5 | 2.3 | 61.0% | 61.2 | 2.5 | 4.4 | 0.4 | 58.5% | NA | NA | 35.1 | 2.8 | 31.7% | 51.2% |
| M10-447 (Naive) | ADA160/80 | 91 | 42.5 | 1.5 | 67.0% | 60.1 | 1.3 | 7.8 | 0.7 | 69.2% | 8.6 | 0.1 | 2.2 | 3.5 | 45.1% | 62.6% |
| M10-447 (Naive) | PBO | 96 | 41.3 | 1.4 | 72.9% | 60.8 | 1.4 | 7.8 | 0.7 | 61.5% | 8.5 | 0.2 | 3.4 | 3.5 | 54.2% | 60.4% |
| NCT01551290 (Naive) | INF5 | 50 | 37.0 | 2.3 | NA | NA | NA | 3.7 | 1.0 | NA | 8.0 | 0.2 | NA | NA | NA | 60.0% |
| NCT01551290 (Naive) | PBO | 49 | 37.0 | 2.3 | NA | NA | NA | 3.7 | 1.0 | NA | 8.0 | 0.2 | NA | NA | NA | 80.0% |
| NCT02039505 | VED300 | 164 | 42.3 | 1.1 | 60.4% | 58.6 | 0.9 | 7.2 | 0.5 | 61.6% | 8.3 | 0.1 | NA | NA | 48.8% | 31.7% |
| NCT02039505 | PBO | 82 | 44.0 | 1.8 | 67.1% | 60.4 | 1.4 | 8.6 | 0.9 | 62.2% | 8.1 | 0.2 | NA | NA | 52.4% | 30.5% |
| OCTAVE 1 | TOF10 | 476 | 41.3 | 0.6 | 58.2% | 72.9 | 0.8 | 6.5 | 1.0 | 52.9% | 9.0 | 0.1 | 4.4 | 3.5 | NA | 45.0% |
| OCTAVE 1 | PBO | 122 | 41.8 | 1.4 | 63.1% | 72.7 | 1.5 | 6.0 | 1.0 | 54.1% | 9.1 | 0.1 | 4.7 | 3.5 | NA | 47.5% |
| OCTAVE 2 | TOF10 | 429 | 41.1 | 0.7 | 60.4% | 74.4 | 0.8 | 6.0 | 1.0 | 49.2% | 9.0 | 0.1 | 4.6 | 3.5 | NA | 46.2% |
| OCTAVE 2 | PBO | 112 | 40.4 | 1.2 | 49.1% | 73.2 | 1.5 | 6.2 | 1.0 | 50.0% | 8.9 | 0.1 | 5.0 | 3.5 | NA | 49.1% |
| PURSUIT-SC (Naive) | GOL200/100 | 258 | 39.7 | 0.9 | 54.3% | NA | NA | 6.4 | 0.4 | 41.5% | 8.7 | 0.1 | 11.5 | 1.0 | 29.5% | 45.3% |
| PURSUIT-SC (Naive) | PBO | 258 | 39.7 | 0.8 | 50.4% | NA | NA | 6.4 | 0.5 | 42.6% | 8.3 | 0.1 | 9.6 | 0.9 | 29.5% | 41.1% |
| SELECTION (Naive) | FIL100 | 277 | 42.0 | 0.8 | 56.7% | NA | NA | 6.7 | 0.4 | NA | 8.6 | 0.1 | 7.8 | 1.0 | 29.6% | 31.0% |
| SELECTION (Naive) | FIL200 | 245 | 42.0 | 0.8 | 50.2% | NA | NA | 7.2 | 0.4 | NA | 8.6 | 0.1 | 8.6 | 1.0 | 29.8% | 30.2% |
| SELECTION (Naive) | PBO | 137 | 41.0 | 1.1 | 63.5% | NA | NA | 6.4 | 0.6 | NA | 8.7 | 0.1 | 5.8 | 0.6 | 29.9% | 30.7% |
| SELECTION (Exposed) | FIL100 | 285 | 43.0 | 0.8 | 65.3% | NA | NA | 9.7 | 0.4 | NA | 9.3 | 0.1 | 11.7 | 1.1 | 21.8% | 46.0% |
| SELECTION (Exposed) | FIL200 | 262 | 43.0 | 0.9 | 56.5% | NA | NA | 9.8 | 0.5 | NA | 9.2 | 0.1 | 12.2 | 0.9 | 23.7% | 46.6% |
| SELECTION (Exposed) | PBO | 142 | 44.0 | 1.3 | 60.6% | NA | NA | 10.2 | 0.7 | NA | 9.3 | 0.1 | 14.0 | 2.0 | 22.5% | 43.7% |
| TRUE NORTH | OZA0.92 | 429 | 41.4 | 0.7 | 57.1% | NA | NA | 6.9 | 0.3 | 37.5% | 8.9 | 0.1 | 4.0 | 0.3 | 0.0% | 27.7% |
| TRUE NORTH | PBO | 216 | 41.9 | 0.9 | 66.2% | NA | NA | 6.8 | 0.5 | 38.0% | 8.9 | 0.1 | 5.0 | 0.5 | 0.0% | 32.4% |
| U-ACCOMPLISH | UPA45 | 345 | 42.1 | 0.8 | 62.0% | 74.0 | 1.0 | 7.3 | 0.3 | 51.0% | 9.3 | 0.1 | 9.3 | 0.8 | 0.3% | 34.2% |
| U-ACCOMPLISH | PBO | 177 | 42.2 | 1.1 | 60.5% | 73.7 | 1.5 | 7.4 | 0.5 | 48.6% | 9.4 | 0.1 | 10.8 | 1.5 | 1.7% | 40.7% |
| U-ACHIEVE Study 2 | UPA45 | 319 | 43.6 | 0.8 | 62.1% | 71.3 | 1.0 | 8.6 | 0.4 | 50.5% | 9.3 | 0.1 | 9.4 | 0.9 | 0.9% | 38.9% |
| U-ACHIEVE Study 2 | PBO | 155 | 44.4 | 1.2 | 62.6% | 74.0 | 1.5 | 9.1 | 0.7 | 51.6% | 9.3 | 0.1 | 12.2 | 1.7 | 1.9% | 39.4% |
| ULTRA-1 (Naive) | ADA160/80 | 130 | 38.2 | 1.2 | 63.8% | 75.5 | 1.2 | 6.1 | 1.0 | 46.2% | 8.8 | 0.1 | 3.3 | 3.5 | 39.2% | 54.6% |
| ULTRA-1 (Naive) | PBO | 130 | 38.9 | 1.1 | 63.1% | 78.7 | 1.5 | 5.4 | 1.0 | 56.2% | 8.7 | 0.1 | 3.2 | 3.5 | 40.0% | 68.5% |
| ULTRA-2 | ADA160/80 | 258 | 39.6 | 0.8 | 55.0% | 75.3 | 1.1 | 8.1 | 0.4 | 46.5% | 8.9 | 0.1 | 14.5 | 2.0 | 36.0% | 58.1% |
| ULTRA-2 | PBO | 260 | 41.3 | 0.8 | 58.5% | 77.1 | 1.1 | 8.5 | 0.5 | 46.2% | 8.9 | 0.1 | 13.1 | 2.3 | 30.8% | 53.8% |
| UNIFI | UST6 | 322 | 41.7 | 0.8 | 60.6% | 73.0 | 1.1 | 8.2 | 0.4 | 47.5% | 8.9 | 0.1 | 4.8 | 0.5 | 27.6% | 52.2% |
| UNIFI | PBO | 319 | 41.2 | 0.8 | 61.8% | 72.9 | 0.9 | 8.0 | 0.4 | 47.2% | 8.9 | 0.1 | 4.7 | 0.4 | 27.9% | 49.2% |

**Abbreviations**: CRP=C-reactive protein; N=number randomised; NA=not available; PBO=placebo; SE=standard error.

Table 9. Baseline patient characteristics data of maintenance populations, by biologic exposure subgroup if available

| **Study (biologic exposure subgroup)** | **Treatment arm** | **N** | **Age (years; mean)** | **Age (years; SE)** | **Male (%)** | **Weight (kg; mean)** | **Weight (kg; SE)** | **Disease duration (years; mean)** | **Disease duration (years; SE)** | **Extensive colitis or pancolitis (%)** | **Total Mayo score (mean)** | **Total Mayo score (SE)** | **CRP (mean)** | **CRP (SE)** | **Concurrent immuno-modulators (%)** | **Concurrent steroids (%)** |
| --- | --- | --- | --- | --- | --- | --- | --- | --- | --- | --- | --- | --- | --- | --- | --- | --- |
| ACT-1 (Naive) | INF10 | 122 | 41.8 | 1.3 | 59.0% | 76.9 | 1.5 | 8.4 | 0.7 | 44.3% | 8.4 | 0.1 | 16.0 | 2.1 | 48.4% | 59.8% |
| ACT-1 (Naive) | INF5 | 121 | 42.4 | 1.3 | 64.5% | 80.0 | 1.6 | 5.9 | 0.5 | 46.3% | 8.5 | 0.2 | 14.0 | 1.7 | 54.5% | 57.9% |
| ACT-1 (Naive) | PBO | 121 | 41.4 | 1.2 | 59.5% | 76.8 | 1.5 | 6.2 | 0.5 | 44.6% | 8.4 | 0.2 | 17.0 | 2.5 | 43.8% | 65.3% |
| GEMINI 1 (Naive) | VED300Q8W | 72 | 41.0 | 1.6 | 54.2% | 76.1 | 2.2 | 5.8 | 0.6 | 33.3% | 8.3 | 0.2 | NA | NA | 41.7% | 54.2% |
| GEMINI 1 (Naive) | VED300Q4W | 73 | 38.3 | 1.5 | 53.4% | 70.3 | 2.0 | 7.0 | 0.7 | 49.3% | 8.2 | 0.2 | NA | NA | 39.7% | 60.3% |
| GEMINI 1 (Naive) | PBO | 79 | 39.5 | 1.6 | 57.0% | 71.3 | 2.1 | 6.4 | 0.6 | 44.3% | 8.4 | 0.2 | NA | NA | 43.0% | 54.4% |
| GEMINI 1 (Exposed/IR) | VED300Q8W | 43 | 41.3 | 1.7 | 55.8% | 79.1 | 2.8 | 6.8 | 0.7 | 65.1% | 8.5 | 0.3 | NA | NA | 16.3% | 60.5% |
| GEMINI 1 (Exposed/IR) | VED300Q4W | 40 | 39.9 | 2.9 | 52.5% | 72.7 | 2.8 | 8.1 | 1.2 | 57.5% | 8.4 | 0.3 | NA | NA | 32.5% | 47.5% |
| GEMINI 1 (Exposed/IR) | PBO | 38 | 41.6 | 2.2 | 55.3% | 81.2 | 3.9 | 9.8 | 1.4 | 57.9% | 8.2 | 0.3 | NA | NA | 34.2% | 60.5% |
| M10-447 (Naive) | ADA40Q2W | 178 | 43.4 | 1.1 | 62.4% | 59.4 | 0.9 | 8.0 | 0.6 | 65.7% | 8.6 | 0.1 | NA | NA | 44.4% | NA |
| M10-447 (Naive) | PBO | 96 | 41.3 | 1.4 | 72.9% | 60.8 | 1.4 | 7.8 | 0.7 | 61.5% | 8.5 | 0.2 | 3.4 | 2.6 | 54.2% | NA |
| NCT02039505 | VED300Q8W | 41 | 43.0 | 2.2 | 51.2% | NA | NA | 8.6 | 1.2 | 68.3% | 8.1 | 0.2 | NA | NA | 53.7% | 31.7% |
| NCT02039505 | PBO | 42 | 42.6 | 2.2 | 54.8% | NA | NA | 8.7 | 1.1 | 54.8% | 7.9 | 0.2 | NA | NA | 50.0% | 35.7% |
| OCTAVE Sustain (Non-IR) | TOF5 | 115 | 43.6 | 1.2 | 54.8% | 73.4 | 1.3 | 6.7 | 0.5 | 42.6% | 2.9 | 0.2 | 0.7 | 2.6 | NA | 47.8% |
| OCTAVE Sustain (Non-IR) | TOF10 | 104 | 42.2 | 1.4 | 59.6% | 74.6 | 1.1 | 7.3 | 0.6 | 49.0% | 3.0 | 0.2 | 0.9 | 2.6 | NA | 44.2% |
| OCTAVE Sustain (Non-IR) | PBO | 109 | 41.9 | 1.3 | 60.6% | 76.2 | 1.2 | 8.0 | 0.8 | 49.5% | 3.0 | 0.2 | 1.0 | 2.6 | NA | 48.6% |
| OCTAVE Sustain (Exposed/IR) | TOF5 | 83 | 39.6 | 1.6 | 48.2% | 73.4 | 1.3 | 9.9 | 0.9 | 63.9% | 3.8 | 0.2 | 0.7 | 2.6 | NA | 55.4% |
| OCTAVE Sustain (Exposed/IR) | TOF10 | 93 | 43.7 | 1.5 | 51.6% | 74.6 | 1.1 | 9.6 | 0.8 | 55.9% | 3.9 | 0.2 | 0.9 | 2.6 | NA | 49.5% |
| OCTAVE Sustain (Exposed/IR) | PBO | 89 | 45.2 | 1.6 | 56.2% | 76.2 | 1.2 | 9.3 | 0.7 | 60.7% | 3.6 | 0.2 | 1.0 | 2.6 | NA | 58.4% |
| PURSUIT-J (Naive) | GOL100 | 32 | 39.3 | 2.1 | 59.4% | 64.6 | 2.6 | 5.4 | 1.4 | 37.5% | 8.0 | 0.3 | 5.3 | 2.6 | 50.0% | 28.1% |
| PURSUIT-J (Naive) | PBO | 31 | 42.9 | 2.6 | 61.3% | 59.5 | 1.7 | 5.7 | 1.4 | 38.7% | 8.0 | 0.3 | 4.1 | 1.4 | 41.9% | 29.0% |
| PURSUIT-M (Naive) | GOL100 | 154 | 39.1 | 1.1 | 57.8% | NA | NA | 7.2 | 0.6 | NA | 8.5 | 0.1 | 8.9 | 1.2 | 31.2% | 53.9% |
| PURSUIT-M (Naive) | GOL50 | 154 | 41.4 | 1.1 | 50.0% | NA | NA | 6.8 | 0.6 | NA | 8.1 | 0.1 | 8.5 | 1.0 | 30.5% | 53.9% |
| PURSUIT-M (Naive) | PBO | 156 | 40.2 | 1.1 | 48.1% | NA | NA | 6.9 | 0.6 | NA | 8.3 | 0.1 | 9.6 | 1.2 | 33.3% | 56.4% |
| SELECTION | FIL100 | 179 | 42.0 | 0.9 | 56.4% | NA | NA | 8.9 | 0.6 | NA | NA | NA | 3.0 | 0.4 | 24.6% | 44.1% |
| SELECTION | FIL200 | 202 | 43.0 | 1.0 | 47.0% | NA | NA | 8.4 | 0.5 | NA | NA | NA | 3.7 | 0.7 | 26.7% | 39.6% |
| SELECTION | PBO | 190 | 42.5 | 1.0 | 51.1% | NA | NA | 8.2 | 0.5 | NA | NA | NA | 3.1 | 0.4 | 26.8% | 40.5% |
| TRUE NORTH | OZA0.92 | 230 | NA | NA | 49.1% | NA | NA | NA | NA | NA | NA | NA | NA | NA | 0.0% | 29.0% |
| TRUE NORTH | PBO | 227 | NA | NA | 46.3% | NA | NA | NA | NA | NA | NA | NA | NA | NA | 0.0% | 29.0% |
| U-ACHIEVE Study 3 | UPA30 | 154 | 42.6 | 1.2 | 55.8% | 73.1 | 1.7 | 8.2 | 0.6 | 55.8% | 9.4 | 0.1 | 8.6 | 1.2 | 0.6% | 37.0% |
| U-ACHIEVE Study 3 | UPA15 | 148 | 42.6 | 1.2 | 64.2% | 74.4 | 1.7 | 8.9 | 0.7 | 55.4% | 9.3 | 0.1 | 8.4 | 1.0 | 0.7% | 37.2% |
| U-ACHIEVE Study 3 | PBO | 149 | 43.3 | 1.2 | 57.0% | 72.2 | 1.5 | 8.7 | 0.7 | 47.0% | 9.3 | 0.1 | 9.8 | 1.3 | 0.0% | 40.3% |
| ULTRA-2 | ADA40Q2W | 258 | 39.6 | 0.8 | 55.0% | 75.3 | 1.1 | 8.1 | 0.4 | 46.5% | 8.9 | 0.1 | 14.5 | 2.0 | 36.0% | 58.1% |
| ULTRA-2 | PBO | 260 | 41.3 | 0.8 | 58.5% | 77.1 | 1.1 | 8.5 | 0.5 | 46.2% | 8.9 | 0.1 | 13.1 | 2.3 | 30.8% | 53.8% |
| UNIFI | UST90Q8W | 176 | 39.5 | 1.0 | 53.4% | 72.0 | 1.4 | 8.1 | 0.5 | 45.4% | 8.9 | 0.1 | 4.0 | 0.6 | 26.1% | 54.0% |
| UNIFI | UST90Q12W | 172 | 40.7 | 1.0 | 55.8% | 73.3 | 1.4 | 8.6 | 0.6 | 46.5% | 8.9 | 0.1 | 3.3 | 0.5 | 25.6% | 48.3% |
| UNIFI | PBO | 175 | 42.0 | 1.0 | 61.1% | 71.7 | 1.1 | 7.5 | 0.5 | 49.1% | 8.7 | 0.1 | 3.4 | 0.5 | 28.0% | 54.3% |
| VISIBLE 1 | VED300Q8W | 54 | 41.6 | 1.9 | 57.4% | 77.0 | 2.3 | 8.2 | 0.8 | 48.1% | 9.0 | 0.3 | NA | NA | NA | 38.9% |
| VISIBLE 1 | PBO | 56 | 39.4 | 1.6 | 60.7% | 74.0 | 2.8 | 7.4 | 0.9 | 44.6% | 9.0 | 0.3 | NA | NA | NA | 42.9% |

**Abbreviations**: CRP=C-reactive protein; IR=inadequate response/intolerance/failed; N=number randomised; NA=not available; PBO=placebo; SE=standard error.

## Appendix 4: Risk of bias assessment

Table 10. Quality assessment of included RCTs

| **Study** | **1. Randomization** | **2. Deviations from intended interventions** | **3. Missing outcome data** | **4. Measurement of the outcome** | **5. Selection of the reported result** | **Overall** |
| --- | --- | --- | --- | --- | --- | --- |
| U-ACHIEVE (NCT02819635) | LOW | LOW | LOW | LOW | LOW | LOW |
| U-ACCOMPLISH (NCT03653026) | LOW | LOW | LOW | LOW | LOW | LOW |
| GEMINI 1 (NCT00783718) | LOW | LOW | LOW | LOW | LOW | LOW |
| VISIBLE 1 (NCT02611830) | LOW | LOW | LOW | LOW | LOW | LOW |
| NCT02039505 | LOW | LOW | LOW | LOW | LOW | LOW |
| UNIFI (NCT02407236) | LOW | LOW | LOW | LOW | LOW | LOW |
| ULTRA-1 (NCT00385736) | LOW | LOW | LOW | LOW | LOW | LOW |
| ULTRA-2 (NCT00408629) | LOW | LOW | LOW | LOW | LOW | LOW |
| M10-447 (NCT00853099) | LOW | LOW | LOW | SOME CONCERNS | LOW | LOW |
| SERENE-UC (NCT02065622) | LOW | LOW | SOME CONCERNS | LOW | LOW | LOW |
| ACT-1 (NCT00036439) | LOW | LOW | LOW | SOME CONCERNS | LOW | LOW |
| ACT-2 (NCT00096655) | LOW | LOW | LOW | SOME CONCERNS | LOW | LOW |
| Japic CTI-060298 | LOW | LOW | LOW | LOW | LOW | LOW |
| Jiang_JCG_2015 | LOW | LOW | LOW | LOW | LOW | LOW |
| PURSUIT-SC (NCT00487539) | LOW | LOW | LOW | SOME CONCERNS | LOW | LOW |
| PURSUIT-M (NCT00488631) | LOW | LOW | LOW | SOME CONCERNS | LOW | LOW |
| PURSUIT-J (NCT01863771) | LOW | LOW | SOME CONCERNS | SOME CONCERNS | LOW | LOW |
| OCTAVE 1 (NCT01465763) | LOW | LOW | LOW | LOW | LOW | LOW |
| OCTAVE 2 (NCT01458951) | LOW | LOW | LOW | LOW | LOW | LOW |
| OCTAVE Sustain (NCT01458574) | LOW | LOW | SOME CONCERNS | LOW | LOW | LOW |
| NCT01551290 | LOW | LOW | LOW | SOME CONCERNS | LOW | LOW |
| SELECTION (NCT02914522) | LOW | LOW | LOW | LOW | LOW | LOW |
| True North Study (NCT02435992) | LOW | LOW | LOW | LOW | LOW | LOW |

Note: Results produced from the Cochrane Risk of Bias tool, version 2.0.[19](#_ENREF_19) LOW indicates that a study is judged to be at a low risk of bias within a domain; SOME CONCERNS indicates that a study is judged to raise some concerns within a domain; HIGH indicates that a study is judged to be at a high risk of bias within a domain.

## Appendix 5: Outcome contributions and definitions

Table 11. Contribution of included induction RCTs to efficacy outcomes evaluated in the NMA (green=bio-naïve outcomes used; blue=bio-exposed outcomes used)

| **Study** | **Included regimen(s) (+PBO)** | **Induction duration (weeks)** | **Endoscopic reading location** | **Handling of missing binary data** | **Clinical remission**  **(****FMS≤2 with no subscore>1) by bio-experience** | | | | **Clinical response**  **(Decrease in FMS ≥3 points and ≥30% from baseline, and a decrease in RBS ≥1 or an absolute RBS ≤1) by bio-experience** | | | | **Endoscopic improvement (****EMS ≤1) by bio-experience** | | | |
| --- | --- | --- | --- | --- | --- | --- | --- | --- | --- | --- | --- | --- | --- | --- | --- | --- |
|  |  |  |  |  | **Naïve** | **Exposed** | **Non-IR** | **IR** | **Naïve** | **Exposed** | **Non-IR** | **IR** | **Naïve** | **Exposed** | **Non-IR** | **IR** |
| ACT-1 (NCT00036439) | INF10  INF5 | 8 | Local | NRI | X |  |  |  | X |  |  |  | X |  |  |  |
| ACT-2 (NCT00096655) | INF10  INF5 | 8 | Local | NRI | X |  |  |  | X |  |  |  | X |  |  |  |
| GEMINI 1 (NCT00783718) | VED300 | 6 | Local | LOCF | X |  |  | X | X |  |  | X | X |  |  | X |
| Japic CTI-060298 | INF5 | 8 | NR | NRI | X |  |  |  | X |  |  |  | X |  |  |  |
| Jiang 2015 | INF5 | 8 | Local | NRI | X |  |  |  | X |  |  |  | X |  |  |  |
| M10-447 (NCT00853099) | ADA160/80 | 8 | NR | NRI | X |  |  |  | X |  |  |  | X |  |  |  |
| NCT01551290 | INF5 | 8 | NR | NR | X |  |  |  | X |  |  |  | X |  |  |  |
| NCT02039505 | VED300 | 10 | Local | NRI | X | X |  |  | X | X |  |  | X | X |  |  |
| OCTAVE 1 (NCT01465763) | TOF10 | 8 | Central | NRI | FM2 | FM2 | FM2 | FM2 | X | X |  |  | X | X | X | X |
| OCTAVE 2 (NCT01458951) | TOF10 | 8 | Central | NRI | FM2 | FM2 | FM2 | FM2 | X | X |  |  | X | X | X | X |
| PURSUIT-SC (NCT0048753) | GOL200/100 | 6 | Local | NRI | X |  |  |  | X |  |  |  | X |  |  |  |
| SELECTION (NCT02914522) | FIL200  FIL100 | 10 | Central | NRI | X | X |  |  | X | X |  |  | X | X |  |  |
| TRUE NORTH (NCT02435992) | OZA0.92 | 10 | Central | NRI | AM2 | AM2 |  |  | AM2 | AM2 |  |  | X | X |  |  |
| U-ACCOMPLISH  (Study M14-675; NCT03653026) | UPA45 | 8 | Central | NRI-C | X | X | AM1 | AM1 | X | X | AM1 | AM1 | X | X | X | X |
| U-ACHIEVE Study 2 (Study M14-234; NCT02819635) | UPA45 | 8 | Central | NRI-C | X | X | AM1 | AM1 | X | X | AM1 | AM1 | X | X | X | X |
| ULTRA-1 (NCT00385736) | ADA160/80 | 8 | Local | NRI | X |  |  |  | X |  |  |  | X |  |  |  |
| ULTRA-2 (NCT00408629) | ADA160/80 | 8 | Local | NRI | X | X |  |  | X | X |  |  | X | X |  |  |
| UNIFI (NCT02407236) | UST6 | 8 | Local/Central | NRI | X | X | X | X | X |  | X | X | X |  | X | X |

**Abbreviations**: AMS=Adapted Mayo score; AM1 remission=AMS≤2, with SFS≤1 and not greater than baseline, RBS=0, and EMS≤1; AM1 response=decrease in AMS ≥2 points and ≥30% from baseline, and a decrease in RBS ≥1 or an absolute RBS ≤1; AM2 remission=SFS≤1 and ≥1-point decrease from baseline, RBS=0, and EMS≤1; AM2 response=decrease in AMS≥2 points and ≥35% from baseline, and a decrease in RBS ≥1 or an absolute RBS ≤1; EMS=endoscopy Mayo subscore; FMS=Full Mayo score; FM2 remission=FMS≤2 with no subscore>1 and RBS=0; LOCF=last observation carried forward; NR=not reported; NRI=non-responder imputation; NRI-C=NRI with multiple imputation to handle missing data due to COVID-19; IR=inadequate response and/or intolerance; PBO=placebo; RBS=rectal bleeding subscore; SFS=stool frequency subscore; X=applicable.

Table 12. Contribution of included induction RCTs to safety outcomes evaluated in the NMA

| **Study** | **Included regimen(s) (+PBO)** | **Safety assessed (weeks)** | **All AEs** | **Discontinuation**  **due to AEs** | **Serious AEs** | **Serious infections** |
| --- | --- | --- | --- | --- | --- | --- |
| ACT-1 (NCT00036439) | INF10  INF5 | NR |  |  |  |  |
| ACT-2 (NCT00096655) | INF10  INF5 | NR |  |  |  |  |
| GEMINI 1 (NCT00783718) | VED300 | 6 | X | X | X | X |
| Japic CTI-060298 | INF5 | 14† | X | X | X | X |
| Jiang 2015 | INF5 | NR |  |  |  |  |
| M10-447 (NCT00853099) | ADA160/80 | 8 | X | X | X | X |
| NCT01551290 | INF5 | NR |  |  |  |  |
| NCT02039505 | VED300 | 10 | X | X | X | X |
| OCTAVE 1 (NCT01465763) | TOF10 | 8 | X | X | X | X |
| OCTAVE 2 (NCT01458951) | TOF10 | 8 | X | X | X | X |
| PURSUIT-SC (NCT00487539) | GOL200/100 | 6 | X | X | X | X |
| SELECTION (NCT02914522) | FIL200  FIL100 | 11* | X | X | X | X |
| TRUE NORTH (NCT02435992) | OZA0.92 | 10 | X | X | X | X |
| U-ACCOMPLISH  (Study M14-675; NCT03653026) | UPA45 | 8 | X | X | X | X |
| U-ACHIEVE Study 2 (Study M14-234; NCT02819635) | UPA45 | 8 | X | X | X | X |
| ULTRA-1 (NCT00385736) | ADA160/80 | 8 | X | X | X | X |
| ULTRA-2 (NCT00408629) | ADA160/80 | 8 | X | X | X | X |
| UNIFI (NCT02407236) | UST6 | 8 | X |  | X | X |

† Outside the 6-to-10-week range set by the NMA PICOS criteria, but were included so that INF and FIL were included in the induction safety networks.

**Abbreviations**: AE=adverse event; NR=not reported; NMA=network meta-analysis; PBO=placebo; RCT=randomised clinical trial; X=applicable.

Table 13. Contribution of included maintenance RCTs to efficacy outcomes evaluated in the NMA (green=bio-naïve outcomes used; blue=bio-exposed outcomes used)

| **Study** | **Included regimen(s) (+PBO)** | **Maintenance duration (weeks)** | **Maintenance study design** | **Handling of missing binary data** | **Endoscopic reading location** | **Clinical remission**  **(FMS≤2 with no subscore>1) by bio-experience** | | | | **Clinical response**  **(Decrease in FMS ≥3 points and ≥30% from baseline, and a decrease in RBS ≥1 or an absolute RBS ≤1) by bio-experience** | | | | **Endoscopic improvement (EMS ≤1) by bio-experience** | | | |
| --- | --- | --- | --- | --- | --- | --- | --- | --- | --- | --- | --- | --- | --- | --- | --- | --- | --- |
|  |  |  |  |  |  | **Naïve** | **Exposed** | **Non-IR** | **IR** | **Naïve** | **Exposed** | **Non-IR** | **IR** | **Naïve** | **Exposed** | **Non-IR** | **IR** |
| ACT-1 (NCT00036439) | INF5  INF10 | 46 | TT | NRI | Local | X  (IP/IT) |  |  |  | X |  |  |  |  |  |  |  |
| GEMINI 1 (NCT00783718) | VED300Q8W  VED300Q4W | 46 | RR | LOCF | Local | X |  |  | X | X |  |  | X | X |  |  | X |
| M10-447 (NCT00853099) | ADA40Q2W | 44 | TT | NRI | NR | X  (ST) |  |  |  | X  (ST) |  |  |  | X  (ST) |  |  |  |
| NCT02039505 | VED300Q8W | 50 | RR | NRI | Local | X | X |  |  | X | X |  |  | X | X |  |  |
| OCTAVE Sustain (NCT01458574) | TOF5  TOF10 | 52 | RR | NRI | Central |  |  | FM2 | FM2 |  |  | X | X |  |  | X | X |
| PURSUIT-J  (NCT01863771) | GOL100 | 54 | RR | LOCF | Local | X(2) |  |  |  | X(2) |  |  |  | X |  |  |  |
| PURSUIT-M  (NCT00488631) | GOL50  GOL100 | 54 | RR | NRI | Local | X |  |  |  | X(2) |  |  |  | X(2) |  |  |  |
| SELECTION (NCT02914522) | FIL100  FIL200 | 47 | RR | NRI | Central | X | X |  |  | X | X |  |  | X | X |  |  |
| SERENE-UC (NCT02065622) | ADA40Q2W  ADA40QW  (no PBO) | 44 | RR | NRI | Central |  |  |  |  |  |  |  |  |  |  |  |  |
| TRUE NORTH (NCT02435992) | OZA0.92 | 42 | RR | NRI | Central | AM2 | AM2 |  |  | AM2 | AM2 |  |  | X | X |  |  |
| U-ACHIEVE Study 3 (Study M14-234; NCT02819635) | UPA15  UPA30 | 52 | RR | NRI-C | Central | X | X | AM1 | AM1 | X | X | AM1 | AM1 | X | X | X | X |
| ULTRA-2 (NCT00408629) | ADA40Q2W | 44 | TT | NRI | Local | X  (IP/ST) | X  (IP/ST) |  |  | X | X |  |  | X  (ST) | X  (ST) |  |  |
| UNIFI (NCT02407236) | UST90Q12W  UST90Q8W | 44 | RR | NRI | Both | X | X | X | X | X |  | X | X | X |  | X | X |
| VISIBLE 1  (NCT02611830) | VED300Q8W | 46 | RR | NRI | Central | X | X |  |  |  |  |  |  |  |  |  |  |

**Abbreviations**: AMS=Adapted Mayo score; AM1 remission=AMS ≤2, with SFS≤1 and not greater than baseline, RBS=0, and endoscopic subscore≤1; AM1 response=decrease in AMS ≥2 points and ≥30% from baseline, and a decrease in RBS ≥1 or an absolute RBS ≤1; AM2 remission=SFS≤1 and ≥1-point decrease from baseline, RBS=0, and EMS≤1; AM2 response=decrease in AMS≥2 points and ≥35% from baseline, and a decrease in RBS ≥1 or an absolute RBS ≤1; FMS=Full Mayo score; FM2 remission=FMS ≤2 with no subscore>1 and RBS=0; IP=imputed PBO outcome; IR=inadequate response/intolerance/failed; IT=imputed treatment outcome; LOCF=last observation carried forward; NRI=non-responder imputation; NRI-C=NRI with multiple imputation to handle missing data due to COVID-19; PBO=placebo; RBS=rectal bleeding score; RR=re-randomised; SFS=stool frequency score; ST=subgroup treatment outcome; TT=treat-through; X=applicable; X(2)=outcome sustained over two post-induction timepoints.

Table 14. Contribution of included maintenance RCTs to safety outcomes evaluated in the NMA

| **Study** | **Included regimen(s)**  **(+PBO)** | **Maintenance or total* duration (weeks)** | **Maintenance study design** | **All AEs** | **AEs leading to discontinuation** | **Serious AEs** | **Serious infections** |
| --- | --- | --- | --- | --- | --- | --- | --- |
| ACT-1 (NCT00036439) | INF5  INF10 | 54† | TT‡ | E-RN | E-RN | E-RN | E-RN |
| GEMINI 1 (NCT00783718) | VED300Q8W  VED300Q4W | 46 | RR | M-R | M-R | M-R | M-R |
| M10-447 (NCT00853099) | ADA40Q2W | 44 | TT‡ | M-RN | M-RN | M-RN |  |
| NCT02039505 | VED300Q8W | 50 | RR | M-R | M-R | M-R | M-R |
| OCTAVE Sustain (NCT01458574) | TOF5  TOF10 | 52 | RR | M-R | M-R | M-R | M-R |
| PURSUIT-J (NCT01863771) | GOL100 | 54 | RR | M-R |  | M-R |  |
| PURSUIT-M (NCT00488631) | GOL50  GOL100 | 54 | RR | M-R | M-R | M-R | M-R |
| SELECTION (NCT02914522) | FIL100  FIL200 | 47 | RR | M-R | M-R | M-R | M-R |
| SERENE-UC (NCT02065622) | ADA40Q2W  ADA40QW  (no PBO) | 44 | RR |  |  |  | M-RN |
| TRUE NORTH (NCT02435992) | OZA0.92 | 42 | RR | M-R | M-R | M-R | M-R |
| U-ACHIEVE Study 3 (Study M14-234; NCT02819635) | UPA15  UPA30 | 52 | RR | M-R | M-R | M-R | M-R |
| ULTRA-2 (NCT00408629) | ADA40Q2W | 44 | TT** | M-RN | M-RN | M-RN | M-RN |
| UNIFI (NCT02407236) | UST90Q12W  UST90Q8W | 44 | RR | M-R | M-R | M-R | M-R |
| VISIBLE 1(NCT02611830) | VED300Q8W | 46 | RR | M-R | M-R | M-R |  |

† Total duration, including induction.

‡ For TT RCTs M10-447 and ULTRA-2, induction numbers were subtracted from the overall numbers to obtain maintenance numbers. For TT RCT ACT-1, induction numbers were not reported so overall numbers were assessed in the maintenance NMA.

**Abbreviations**: AE=adverse event; E-RN=entire period, induction responders and non-responders; M-R=maintenance period, induction responders; M-RN=maintenance period, induction responders and non-responders; NMA=network meta-analysis; PBO=placebo; RCT=randomised clinical trial; RR=re-randomised; TT=treat-through.

Table 15. Mayo score-based outcome definitions used across included RCTs

| **Outcome code** | **RCT (Treatment)** | **FMS** | **AMS** | | **SFS** | **RBS** | **EMS** | **PGA** |
| --- | --- | --- | --- | --- | --- | --- | --- | --- |
| **Clinical remission** | | | | | | | | |
| Base case (FM1) | All except:  OCTAVE 1 (TOF)  OCTAVE 2 (TOF)  TRUE NORTH (OZA) | ≤2 | NA | | ≤1 | ≤1 | ≤1 | ≤1 |
| FM2 | OCTAVE 1 (TOF)  OCTAVE 2 (TOF) |  |  |  |  | 0 |  |  |
| AM1 | U-ACCOMPLISH (UPA)†  U-ACHIEVE (UPA)† | NA | ≤2 | | ≤1 & not greater than baseline |  |  | NA |
| AM2 | TRUE NORTH (OZA)  SELECTION (FIL)‡ |  | NA | | ≤1 & ≥1-point decrease from baseline |  |  |  |
| **Clinical response** | | | | | | | | |
| Base case (FM1) | All except:  TRUE NORTH (OZA) | Decrease ≥3 points and ≥30% from baseline | | NA | NA | Decrease ≥1 or an absolute ≤1 | NA | NA |
| AM1 | U-ACCOMPLISH (UPA)†  U-ACHIEVE (UPA)† | NA | | Decrease ≥2 points and ≥30% from baseline |  |  |  |  |
| AM2 | TRUE NORTH (OZA) |  |  | Decrease ≥2 points and ≥35% from baseline |  |  |  |  |

† Base case outcomes obtained from ad hoc analyses.

‡ Base case outcomes also reported in study publication.

**Abbreviations**: AMS=Adapted Mayo score; EMS=endoscopy Mayo subscore; FIL=filgotinib; FMS=Full Mayo score; NA=not applicable; OZA=ozanimod; PGA=Physician’s global assessment subscore; RCT=randomised clinical trial; RBS=rectal bleeding subscore; SFS=stool frequency subscore; TOF=tofacitinib; UPA=upadacitinib.

For endoscopic improvement, there was no deviation from the base case definition of ‘EMS ≤1’.

## Appendix 6: NMA datasets

Table 16. Efficacy outcomes data of induction bio-naïve populations

| **Study** | **Treatment arm** | **Clinical remission N** | **Clinical remission n** | **Clinical response N** | **Clinical response n** | **Endoscopic improvement N** | **Endoscopic improvement n** |
| --- | --- | --- | --- | --- | --- | --- | --- |
| ACT-1 | INF10 | 122 | 39 | 122 | 75 | 122 | 72 |
| ACT-1 | INF5 | 121 | 47 | 121 | 84 | 121 | 75 |
| ACT-1 | PBO | 121 | 18 | 121 | 45 | 121 | 41 |
| ACT-2 | INF10 | 120 | 33 | 120 | 83 | 120 | 74 |
| ACT-2 | INF5 | 121 | 41 | 121 | 78 | 121 | 73 |
| ACT-2 | PBO | 123 | 7 | 123 | 36 | 123 | 38 |
| GEMINI 1 | VED300 | 130 | 30 | 130 | 69 | 130 | 64 |
| GEMINI 1 | PBO | 76 | 5 | 76 | 20 | 76 | 19 |
| Japic CTI-060298 | INF5 | 104 | 21 | 104 | 57 | 104 | 48 |
| Japic CTI-060298 | PBO | 104 | 11 | 104 | 37 | 104 | 29 |
| Jiang 2015 | INF5 | 41 | 22 | 41 | 32 | 41 | 24 |
| Jiang 2015 | PBO | 41 | 9 | 41 | 15 | 41 | 10 |
| M10-447 | ADA160/80 | 90 | 9 | 90 | 45 | 90 | 40 |
| M10-447 | PBO | 96 | 11 | 96 | 34 | 96 | 29 |
| NCT01551290 | INF5 | 50 | 11 | 50 | 32 | 50 | 17 |
| NCT01551290 | PBO | 49 | 5 | 49 | 16 | 49 | 8 |
| NCT02039505 | VED300 | 79 | 22 | 79 | 42 | 79 | 38 |
| NCT02039505 | PBO | 41 | 6 | 41 | 15 | 41 | 13 |
| OCTAVE 1 | TOF10 | 222 | 56 | 222 | 147 | 222 | 88 |
| OCTAVE 1 | PBO | 57 | 9 | 57 | 28 | 57 | 15 |
| OCTAVE 2 | TOF10 | 195 | 43 | 195 | 120 | 195 | 71 |
| OCTAVE 2 | PBO | 47 | 4 | 47 | 15 | 47 | 9 |
| PURSUIT-SC | GOL200/100 | 253 | 45 | 253 | 129 | 253 | 107 |
| PURSUIT-SC | PBO | 251 | 16 | 251 | 76 | 251 | 72 |
| SELECTION | FIL100 | 277 | 47 | 277 | 164 | 277 | 73 |
| SELECTION | FIL200 | 245 | 60 | 245 | 163 | 245 | 83 |
| SELECTION | PBO | 137 | 17 | 137 | 64 | 137 | 28 |
| TRUE NORTH | OZA0.92 | 299 | 66 | 299 | 157 | 299 | 97 |
| TRUE NORTH | PBO | 151 | 10 | 151 | 44 | 151 | 18 |
| U-ACCOMPLISH | UPA45 | 166 | 54 | 166 | 129 | 166 | 85 |
| U-ACCOMPLISH | PBO | 81 | 3 | 81 | 28 | 81 | 9 |
| U-ACHIEVE Study 2 | UPA45 | 145 | 41 | 145 | 117 | 145 | 67 |
| U-ACHIEVE Study 2 | PBO | 72 | 4 | 72 | 26 | 72 | 10 |
| ULTRA-1 | ADA160/80 | 130 | 24 | 130 | 71 | 130 | 61 |
| ULTRA-1 | PBO | 130 | 12 | 130 | 58 | 130 | 54 |
| ULTRA-2 | ADA160/80 | 150 | 32 | 150 | 89 | 150 | 74 |
| ULTRA-2 | PBO | 145 | 16 | 145 | 56 | 145 | 51 |
| UNIFI | UST6 | 147 | 27 | 147 | 98 | 147 | 49 |
| UNIFI | PBO | 151 | 15 | 151 | 54 | 151 | 32 |

**Abbreviations**: N=number assessed; n=number with event; PBO=placebo.

Table 17. Efficacy outcomes data of induction bio-exposed populations

| **Study** | **Treatment arm** | **Clinical remission N** | **Clinical remission n** | **Clinical response N** | **Clinical response n** | **Endoscopic improvement N** | **Endoscopic improvement n** |
| --- | --- | --- | --- | --- | --- | --- | --- |
| GEMINI 1 | VED300 | 82 | 8 | 82 | 32 | 82 | 25 |
| GEMINI 1 | PBO | 63 | 2 | 63 | 13 | 63 | 13 |
| NCT02039505 | VED300 | 85 | 8 | 85 | 23 | 85 | 22 |
| NCT02039505 | PBO | 41 | 4 | 41 | 12 | 41 | 12 |
| OCTAVE 1 | TOF10 | 254 | 32 | 254 | 138 | 254 | 61 |
| OCTAVE 1 | PBO | 65 | 1 | 65 | 12 | 65 | 4 |
| OCTAVE 2 | TOF10 | 234 | 28 | 234 | 116 | 234 | 51 |
| OCTAVE 2 | PBO | 65 | 0 | 65 | 17 | 65 | 4 |
| SELECTION | FIL100 | 285 | 17 | 285 | 102 | 285 | 37 |
| SELECTION | FIL200 | 262 | 25 | 262 | 139 | 262 | 45 |
| SELECTION | PBO | 142 | 6 | 142 | 25 | 142 | 11 |
| TRUE NORTH | OZA0.92 | 130 | 13 | 130 | 48 | 130 | 20 |
| TRUE NORTH | PBO | 65 | 3 | 65 | 12 | 65 | 7 |
| U-ACCOMPLISH | UPA45 | 175 | 39 | 175 | 125 | 175 | 65 |
| U-ACCOMPLISH | PBO | 93 | 1 | 93 | 15 | 93 | 5 |
| U-ACHIEVE Study 2 | UPA45 | 174 | 32 | 174 | 118 | 174 | 49 |
| U-ACHIEVE Study 2 | PBO | 82 | 0 | 82 | 11 | 82 | 1 |
| ULTRA-2 | ADA160/80 | 98 | 9 | 98 | 36 | 98 | 28 |
| ULTRA-2 | PBO | 101 | 7 | 101 | 29 | 101 | 27 |
| UNIFI | UST6 | 175 | 23 | 166 | 95 | 166 | 35 |
| UNIFI | PBO | 168 | 2 | 161 | 44 | 161 | 11 |

**Abbreviations**: N=number assessed; n=number with event; PBO=placebo.

Table 18. Safety outcomes data of induction overall populations

| **Study** | **Treatment arm** | **Safety analysis N** | **All AEs n** | **Discontinuation due to AEs n** | **Serious AEs n** | **Serious infections n** |
| --- | --- | --- | --- | --- | --- | --- |
| ACT-1 | INF10 | NA | NA | NA | NA | NA |
| ACT-1 | INF5 | NA | NA | NA | NA | NA |
| ACT-1 | PBO | NA | NA | NA | NA | NA |
| ACT-2 | INF10 | NA | NA | NA | NA | NA |
| ACT-2 | INF5 | NA | NA | NA | NA | NA |
| ACT-2 | PBO | NA | NA | NA | NA | NA |
| GEMINI 1 | VED300 | 225 | 90 | 0 | 5 | 1 |
| GEMINI 1 | PBO | 149 | 63 | 4 | 10 | 3 |
| Japic CTI-060298 | INF5 | 104 | 85 | 5 | 9 | 1 |
| Japic CTI-060298 | PBO | 104 | 86 | 8 | 13 | 2 |
| Jiang 2015 | INF5 | NA | NA | NA | NA | NA |
| Jiang 2015 | PBO | NA | NA | NA | NA | NA |
| M10-447 | ADA160/80 | 90 | 40 | 6 | 4 | 3 |
| M10-447 | PBO | 96 | 45 | 4 | 7 | 0 |
| NCT01551290 | INF5 | NA | NA | NA | NA | NA |
| NCT01551290 | PBO | NA | NA | NA | NA | NA |
| NCT02039505 | VED300 | 164 | 82 | 8 | 10 | 1 |
| NCT02039505 | PBO | 82 | 43 | 2 | 4 | 2 |
| OCTAVE 1 | TOF10 | 476 | 269 | 18 | 16 | 6 |
| OCTAVE 1 | PBO | 122 | 73 | 2 | 5 | 0 |
| OCTAVE 2 | TOF10 | 429 | 232 | 17 | 18 | 1 |
| OCTAVE 2 | PBO | 112 | 59 | 8 | 9 | 0 |
| PURSUIT-SC | GOL200/100 | 331 | 124 | 1 | 9 | 1 |
| PURSUIT-SC | PBO | 330 | 126 | 3 | 20 | 6 |
| SELECTION | FIL100 | 562 | 283 | 20 | 28 | 6 |
| SELECTION | FIL200 | 507 | 272 | 23 | 22 | 3 |
| SELECTION | PBO | 279 | 157 | 14 | 13 | 3 |
| TRUE NORTH | OZA0.92 | 429 | 172 | 14 | 17 | 4 |
| TRUE NORTH | PBO | 216 | 82 | 7 | 7 | 1 |
| U-ACCOMPLISH | UPA45 | 344 | 182 | 6 | 11 | 2 |
| U-ACCOMPLISH | PBO | 177 | 70 | 9 | 8 | 1 |
| U-ACHIEVE Study 2 | UPA45 | 319 | 180 | 6 | 8 | 5 |
| U-ACHIEVE Study 2 | PBO | 155 | 93 | 14 | 9 | 2 |
| ULTRA-1 | ADA160/80 | 223 | 112 | 12 | 9 | 0 |
| ULTRA-1 | PBO | 223 | 108 | 12 | 17 | 3 |
| ULTRA-2 | ADA160/80 | 247 | 144 | 10 | 15 | 3 |
| ULTRA-2 | PBO | 246 | 163 | 18 | 21 | 3 |
| UNIFI | UST6 | 320 | 162 | NA | 11 | 1 |
| UNIFI | PBO | 319 | 153 | NA | 22 | 5 |

**Abbreviations**: N=number assessed; NA=not available; n=number with event; PBO=placebo.

Table 19. Efficacy outcomes data of maintenance bio-naïve populations

| **Study** | **Treatment arm** | **Clinical remission N** | **Clinical remission n** | **Clinical response N** | **Clinical response n** | **Endoscopic improvement N** | **Endoscopic improvement n** |
| --- | --- | --- | --- | --- | --- | --- | --- |
| ACT-1 | INF10 | 75 | 24 | 75 | 45 | NA | NA |
| ACT-1 | INF5 | 84 | 26 | 84 | 47 | NA | NA |
| ACT-1 | PBO | 45 | 11 | 45 | 17 | NA | NA |
| GEMINI 1 | VED300Q4W | 73 | 35 | 73 | 41 | 73 | 44 |
| GEMINI 1 | VED300Q8W | 72 | 33 | 72 | 47 | 72 | 43 |
| GEMINI 1 | PBO | 79 | 15 | 79 | 21 | 79 | 19 |
| M10-447 | ADA40Q2W | NA | NA | NA | NA | NA | NA |
| M10-447 | PBO | NA | NA | NA | NA | NA | NA |
| NCT02039505 | VED300Q8W | 24 | 13 | 24 | 16 | 24 | 15 |
| NCT02039505 | PBO | 28 | 10 | 28 | 10 | 28 | 10 |
| OCTAVE Sustain | TOF10 | 104 | 46 | 104 | 67 | 104 | 53 |
| OCTAVE Sustain | TOF5 | 115 | 48 | 115 | 65 | 115 | 49 |
| OCTAVE Sustain | PBO | 109 | 12 | 109 | 27 | 109 | 15 |
| PURSUIT-J | GOL100 | 32 | 16 | 32 | 18 | 32 | 20 |
| PURSUIT-J | PBO | 31 | 2 | 31 | 6 | 31 | 5 |
| PURSUIT-M | GOL100 | 151 | 51 | 151 | 75 | 151 | 64 |
| PURSUIT-M | GOL50 | 151 | 50 | 151 | 71 | 151 | 63 |
| PURSUIT-M | PBO | 154 | 34 | 154 | 48 | 154 | 41 |
| SELECTION | FIL100 | 105 | 26 | 105 | 61 | 105 | 32 |
| SELECTION | FIL200 | 107 | 49 | 107 | 80 | 107 | 57 |
| SELECTION | PBO | 108 | 16 | 108 | 50 | 108 | 23 |
| TRUE NORTH | OZA0.92 | 154 | 63 | 154 | 96 | 154 | 77 |
| TRUE NORTH | PBO | 158 | 35 | 158 | 76 | 158 | 48 |
| U-ACHIEVE Study 3 | UPA15 | 75 | 30 | 75 | 49 | 75 | 39 |
| U-ACHIEVE Study 3 | UPA30 | 77 | 37 | 77 | 62 | 77 | 52 |
| U-ACHIEVE Study 3 | PBO | 65 | 12 | 65 | 19 | 65 | 15 |
| ULTRA-2 | ADA40Q2W | 89 | 28 | 89 | 44 | NA | NA |
| ULTRA-2 | PBO | 56 | 16 | 56 | 24 | NA | NA |
| UNIFI | UST90Q12W | 95 | 45 | 95 | 73 | 95 | 52 |
| UNIFI | UST90Q8W | 79 | 40 | 79 | 61 | 79 | 46 |
| UNIFI | PBO | 84 | 27 | 84 | 44 | 84 | 30 |
| VISIBLE 1 | VED300Q8W | 32 | 17 | NA | NA | NA | NA |
| VISIBLE 1 | PBO | 37 | 7 | NA | NA | NA | NA |

**Abbreviations**: N=number assessed; NA=not available; n=number with event; PBO=placebo.

Table 20. Efficacy outcomes data of maintenance bio-exposed populations

| **Study** | **Treatment arm** | **Clinical remission N** | **Clinical remission n** | **Clinical response N** | **Clinical response n** | **Endoscopic improvement N** | **Endoscopic improvement n** |
| --- | --- | --- | --- | --- | --- | --- | --- |
| GEMINI 1 | VED300Q4W | 40 | 14 | 40 | 17 | 40 | 19 |
| GEMINI 1 | VED300Q8W | 43 | 16 | 43 | 20 | 43 | 18 |
| GEMINI 1 | PBO | 38 | 2 | 38 | 6 | 38 | 3 |
| NCT02039505 | VED300Q8W | 17 | 10 | 17 | 11 | 17 | 11 |
| NCT02039505 | PBO | 14 | 3 | 14 | 5 | 14 | 4 |
| OCTAVE Sustain | TOF10 | 93 | 34 | 93 | 55 | 93 | 37 |
| OCTAVE Sustain | TOF5 | 83 | 20 | 83 | 37 | 83 | 25 |
| OCTAVE Sustain | PBO | 89 | 10 | 89 | 13 | 89 | 11 |
| SELECTION | FIL100 | 67 | 13 | 67 | 26 | 67 | 14 |
| SELECTION | FIL200 | 92 | 20 | 92 | 53 | 92 | 24 |
| SELECTION | PBO | 79 | 5 | 79 | 17 | 79 | 9 |
| TRUE NORTH | OZA0.92 | 76 | 22 | 76 | 42 | 76 | 28 |
| TRUE NORTH | PBO | 69 | 7 | 69 | 17 | 69 | 12 |
| U-ACHIEVE Study 3 | UPA15 | 73 | 30 | 73 | 43 | 73 | 33 |
| U-ACHIEVE Study 3 | UPA30 | 77 | 36 | 77 | 54 | 77 | 43 |
| U-ACHIEVE Study 3 | PBO | 84 | 4 | 84 | 14 | 84 | 7 |
| ULTRA-2 | ADA40Q2W | 36 | 8 | 36 | 15 | NA | NA |
| ULTRA-2 | PBO | 29 | 3 | 29 | 6 | NA | NA |
| UNIFI | UST90Q12W | 77 | 21 | 70 | 39 | 70 | 18 |
| UNIFI | UST90Q8W | 97 | 37 | 91 | 59 | 91 | 41 |
| UNIFI | PBO | 91 | 15 | 88 | 34 | 88 | 20 |
| VISIBLE 1 | VED300Q8W | 22 | 6 | NA | NA | NA | NA |
| VISIBLE 1 | PBO | 19 | 1 | NA | NA | NA | NA |

**Abbreviations:** N=number assessed; NA=not available; n=number with event; PBO=placebo.

Table 21. Imputed RR data of TT trials included in the maintenance NMA

| **Study** | **Treatment arm** | **N randomised** | **N**  **Induction responders** | **Clinical response \|**  **Induction response** | | **Clinical remission \|**  **Induction response** | | **Endoscopic improvement \|**  **Induction response** | |
| --- | --- | --- | --- | --- | --- | --- | --- | --- | --- |
|  |  |  |  | **n** | **Notes** | **n** | **Notes** | **n** | **Notes** |
| ACT-1 (Naïve) | INF10 | 122 | 75 | 45 | SUB | 24 | IMP (1) | NR |  |
| ACT-1 (Naïve) | INF5 | 121 | 84 | 47 | SUB | 26 | IMP (2) | NR |  |
| ACT-1 (Naïve) | PBO | 121 | 45 | 17 | SUB | 11 | IMP (3) | NR |  |
| ULTRA-2 (Exposed) | ADA40Q2W | 98 | 36 | 15 | SUB | 8 | SUB | 11 | SUB |
| ULTRA-2 (Exposed) | PBO | 101 | 29 | 6 | SUB | 3 | IMP (4) | NR |  |
| ULTRA-2 (Naïve) | ADA40Q2W | 150 | 89 | 44 | SUB | 28 | SUB | 40 | SUB |
| ULTRA-2 (Naïve) | PBO | 145 | 56 | 24 | SUB | 16 | IMP (5) | NR |  |
| M10-447 (Naïve) | ADA160/80/40 + ADA80/40 | 177 | 82 | 50 | SUB | 38 | SUB (6) | 47 | SUB |

**Notes:**

1. Assumed same proportion of induction responders in clinical remission as ADA in ULTRA-2 (Naïve) (24=75*(28/89)).
2. Assumed same proportion of induction responders in clinical remission as ADA in ULTRA-2 (Naïve) (26=84*(28/89)).
3. Assumed to be the same proportion of responders to remitters as RR bio-naive PBO arms (11=17*(sum(15+10+27+34+12)/sum(21+10+44+48+27)). Note that only the PBO arms of RR maintenance trials that 1) enrolled only FMS responders from induction and 2) assessed outcomes (response and remission) after 40 to 54 weeks of maintenance treatment were used in the ratio calculation. As such, the following bio-naïve RR maintenance trials’ PBO arms were not used: 1) PURSUIT-J (both outcomes were required to be sustained over two maintenance timepoints, namely Week 30 and 54); 2) TRUE NORTH and U-ACHIEVE (both trials enrolled AMS responders from induction); and 3) SELECTION (enrolled FMS responders and/or AMS remitters from induction).
4. Assumed to be the same proportion of responders to remitters as RR bio-exposed PBO arms (3=6*(sum(2+3+15+10)/sum(6+5+34+13)). Like in Note 3, the following bio-exposed RR maintenance trials’ PBO arms were not used: 1) TRUE NORTH and U-ACHIEVE (both trials enrolled AMS responders from induction) and 2) SELECTION (enrolled FMS responders and/or AMS remitters from induction).
5. Assumed to be the same proportion of responders to remitters as RR bio-naive PBO arms (16=24*(sum(15+10+27+34+12)/sum(21+10+44+48+27)). Like in Note 3, the PBO arms of PURSUIT-J, TRUE NORTH, U-ACHIEVE, and SELECTION were not used in the ratio calculation.
6. For M10-447, outcomes for induction responders were only reported for ADA and not PBO, so the study cannot be included in the maintenance efficacy NMA.

**Abbreviations**: AMS=Adapted Mayo score; FMS=Full Mayo score; IMP=imputed; PBO=placebo; NMA=network meta-analysis; NR=not reported/cannot be imputed; RR=re-randomised; SUB=secondary results reported in study publications; TT=treat-through.

Table 22. Safety outcomes data of maintenance overall populations

| **Study** | **Treatment arm** | **Safety analysis N** | **All AEs n** | **Discontinuation due to AEs n** | **Serious AEs n** | **Serious infections n** |
| --- | --- | --- | --- | --- | --- | --- |
| ACT-1 | INF10 | 122 | 111 | 11 | 29 | 8 |
| ACT-1 | INF5 | 121 | 106 | 10 | 26 | 3 |
| ACT-1 | PBO | 121 | 103 | 11 | 31 | 5 |
| GEMINI 1 | VED300Q4W | 125 | 101 | 6 | 11 | 2 |
| GEMINI 1 | VED300Q8W | 122 | 100 | 7 | 10 | 3 |
| GEMINI 1 | PBO | 126 | 106 | 15 | 20 | 4 |
| M10-447 | ADA40Q2W | 177 | 54 | 11 | 18 | NA |
| M10-447 | PBO | 96 | 22 | 1 | 5 | NA |
| NCT02039505 | VED300Q8W | 41 | 36 | 2 | 4 | 1 |
| NCT02039505 | PBO | 42 | 33 | 6 | 3 | 1 |
| OCTAVE Sustain | TOF10 | 196 | 156 | 19 | 11 | 1 |
| OCTAVE Sustain | TOF5 | 198 | 143 | 18 | 10 | 2 |
| OCTAVE Sustain | PBO | 198 | 149 | 37 | 13 | 2 |
| PURSUIT-J | GOL100 | 32 | 31 | NA | 1 | NA |
| PURSUIT-J | PBO | 31 | 22 | NA | 4 | NA |
| PURSUIT-M | GOL100 | 154 | 113 | 14 | 22 | 5 |
| PURSUIT-M | GOL50 | 154 | 112 | 8 | 13 | 5 |
| PURSUIT-M | PBO | 156 | 103 | 10 | 12 | 3 |
| SELECTION | FIL100 | 179 | 108 | 10 | 8 | 3 |
| SELECTION | FIL200 | 202 | 135 | 7 | 9 | 2 |
| SELECTION | PBO | 190 | 119 | 6 | 7 | 2 |
| SERENE-UC | ADA40Q2W | 302 | NA | NA | NA | 10 |
| SERENE-UC | ADA40QW | 304 | NA | NA | NA | 9 |
| TRUE NORTH | OZA0.92 | 230 | 113 | 3 | 12 | 2 |
| TRUE NORTH | PBO | 227 | 83 | 6 | 18 | 4 |
| U-ACHIEVE Study 3 | UPA15 | 148 | 115 | 6 | 10 | 5 |
| U-ACHIEVE Study 3 | UPA30 | 154 | 121 | 10 | 9 | 4 |
| U-ACHIEVE Study 3 | PBO | 149 | 113 | 17 | 19 | 6 |
| ULTRA-2 | ADA40Q2W | 257 | 69 | 13 | 16 | 1 |
| ULTRA-2 | PBO | 260 | 55 | 16 | 11 | 2 |
| UNIFI | UST90Q12W | 172 | 119 | 9 | 13 | 6 |
| UNIFI | UST90Q8W | 176 | 136 | 5 | 15 | 3 |
| UNIFI | PBO | 175 | 138 | 20 | 17 | 4 |
| VISIBLE 1 | VED300Q8W | 54 | 41 | 2 | 7 | NA |
| VISIBLE 1 | PBO | 56 | 43 | 5 | 6 | NA |

**Abbreviations**: N=number assessed; NA=not available; n=number with event; PBO=placebo.

## Appendix 7: Model specifications and selections

Table 23: Model specifications and selections

| **Phase** | **Outcome** | **Biologic exposure** | **Selected logit model^1^** | **Baseline assumption^2^** | **Prior distribution**  **for sd^3^** | **Baseline model distribution^4^** | **PBO rate sig*** | **Baseline-risk adjustment** |
| --- | --- | --- | --- | --- | --- | --- | --- | --- |
| Induction | Clinical response | Naïve | **REA** | Independent | Uniform | Predictive | **Yes** | **Significant (REA and FEA)** |
|  |  | Exposed | RE | Independent | Half-normal | Predictive | No | Did not converge (REA and FEA; not sig) |
|  | Clinical remission | Naïve | RE | Independent | Uniform | Predictive | No | **Not significant (REA and FEA)** |
|  |  | Exposed | RE | Exchangeable | Half-normal | Posterior | No | Did not run (REA)/converge (FEA; not sig) |
|  | Endoscopic improvement | Naïve | RE | Independent | Uniform | Posterior | **Yes** | **Not significant (REA and FEA)** |
|  |  | Exposed | RE | Independent | Half-normal | Posterior | **Yes**† | Did not run (REA)/converge (FEA; not sig) |
|  | All AEs | Overall | **REA** | Independent | Half-normal | Posterior | **Yes** | **Significant (REA and FEA)** |
|  | Discontinuation due to AEs | Overall | RE | Independent | Half-normal | Posterior | **Yes**† | Did not converge (REA and FEA; not sig) |
|  | Serious AEs | Overall | RE | Independent | Half-normal | Predictive | No | **Not significant (REA)**/did not converge (FEA; not sig) |
|  | Serious infections | Overall | RE | Exchangeable | Half-normal | Predictive | No | Did not run (REA and FEA) |
| Maintenance | Clinical response | Naïve | RE | Independent | Half-normal | Predictive | **Yes**† | Did not run (REA)/converge (FEA; not sig) |
|  |  | Exposed | RE | Independent | Half-normal | Posterior | **Yes**† | Did not run (REA and FEA) |
|  | Clinical remission | Naïve | RE | Independent | Half-normal | Predictive | **Yes**† | Did not converge (REA; sig)/significant (FEA) |
|  |  | Exposed | RE | Independent | Half-normal | Posterior | No | Did not run (REA)/not significant (FEA) |
|  | Endoscopic improvement | Naïve | RE | Independent | Half-normal | Predictive | **Yes**† | Did not converge (REA and FEA; not sig) |
|  |  | Exposed | RE | Independent | Half-normal | Posterior | No | Did not run (REA and FEA) |
|  | All AEs | Overall | RE | Independent | Half-normal | Posterior | **Yes**† | Did not converge (REA and FEA; not sig) |
|  | Discontinuation due to AEs | Overall | RE | Independent | Half-normal | Posterior | **Yes** | **Not significant (REA)/**did not converge (FEA; not sig) |
|  | Serious AEs | Overall | RE | Independent | Half-normal | Posterior | **Yes** | **Not significant (REA)/**did not converge (FEA; sig) |
|  | Serious infections | Overall | RE | Independent | Half-normal | Posterior | No | Did not run (REA and FEA) |

* Statistical significance with Wald test p-value<0.05

† FE RD models were tested for networks with significant PBO rate heterogeneity, but baseline-risk adjustment was inconclusive (i.e., did not converge/run)

Notes:

1. Model fit statistics determined the selection between FE and RE models; all else equal, the RE model was selected over the FE model. The baseline risk-adjusted version was then selected if its beta coefficient (B) was significant AND sd decreased (for RE models).
2. An exchangeable baseline assumption with a half-normal (0, 0.32^2^) prior for heterogeneity was used if ≥1 reference/PBO arms(s) in the network has a zero value (i.e., no events).
3. Instead of the default uniform (0, 5) prior, a half-normal (0, 0.32^2^) prior for between-study heterogeneity sd was used if most (≥50%) of treatments in the network were informed by a single study.
4. The posterior distribution of the baseline model was used if the 95% CrI of the PBO absolute rate from the model’s predictive distribution differed from the median rate by greater than 2 factors.

**Abbreviations**: AE=adverse event; FE=fixed effects unadjusted model; FEA=fixed effects adjusted model; PBO=placebo; RD=risk difference; RE=random effects unadjusted model; REA=random effects adjusted model; sd=between-study standard deviation.

## Appendix 8: Bio-naïve efficacy OR league tables

Figure 6. OR league table for bio-naïve clinical response induction (REA model)


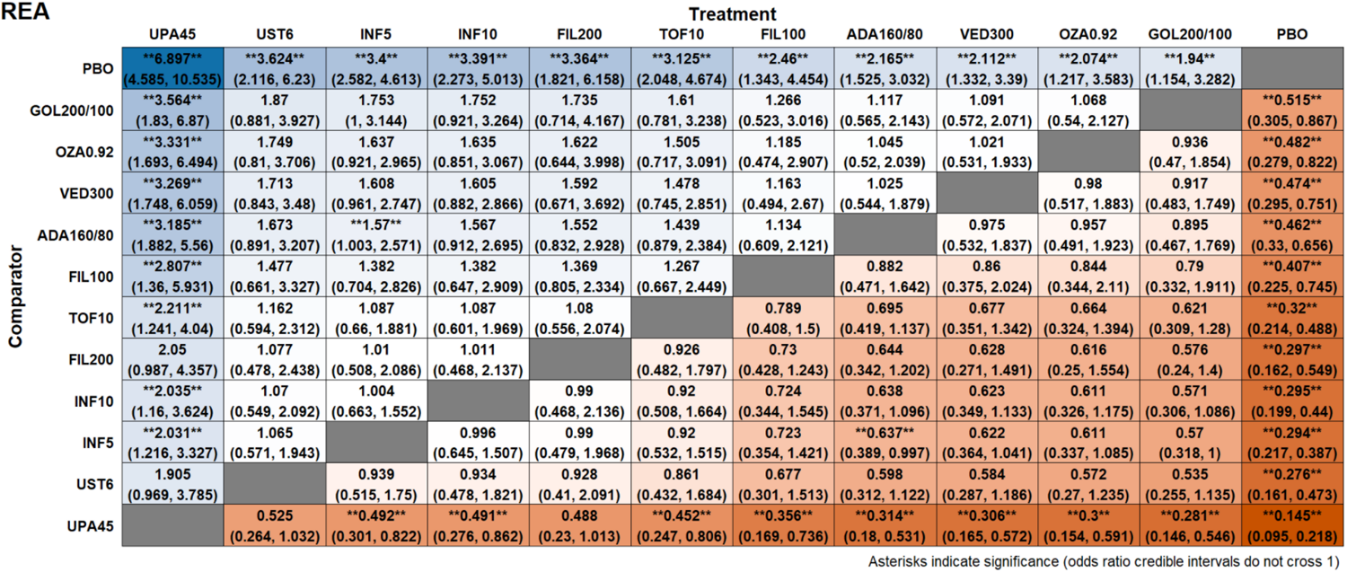


Figure 7. OR league table for bio-naïve clinical response maintenance (RE model)


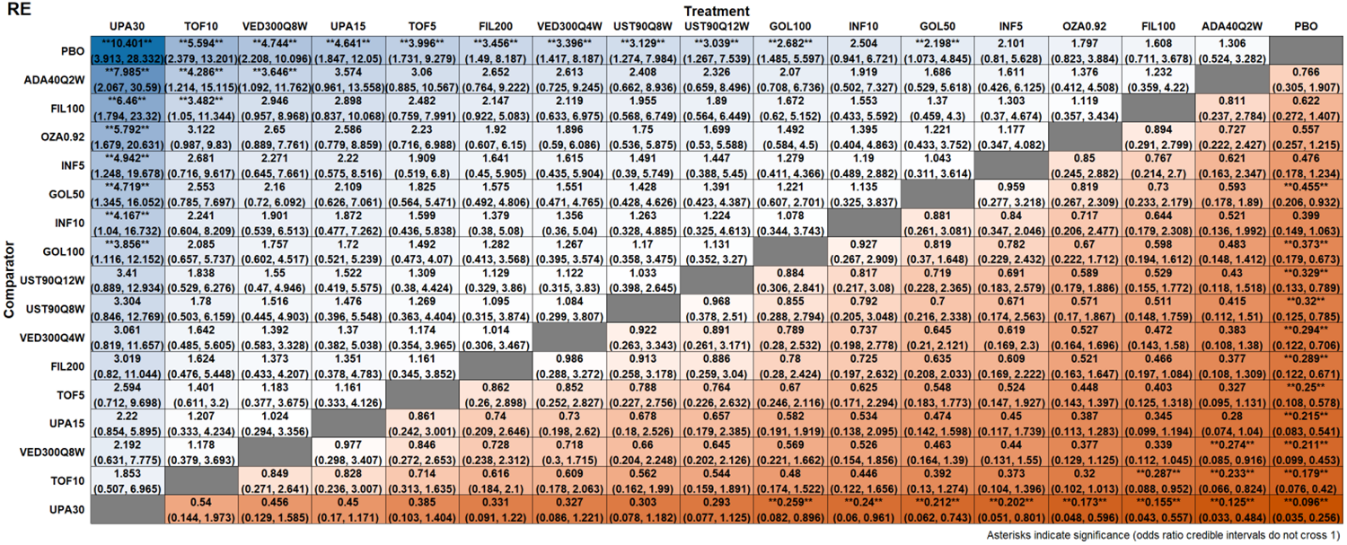


Figure 8. OR league table for bio-naïve clinical remission induction (RE model)


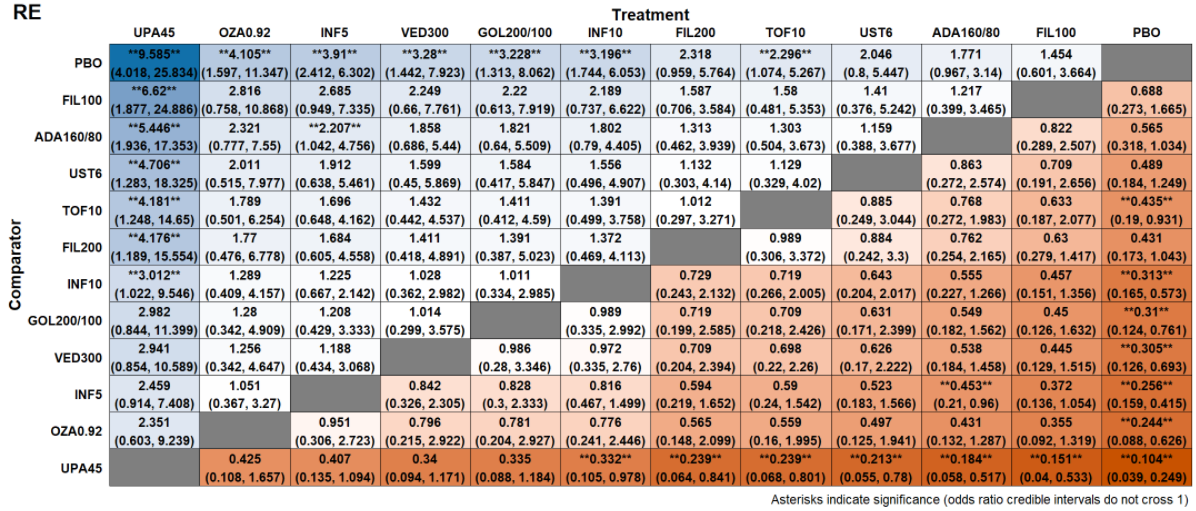


Figure 9. OR league table for bio-naïve clinical remission maintenance (RE model)


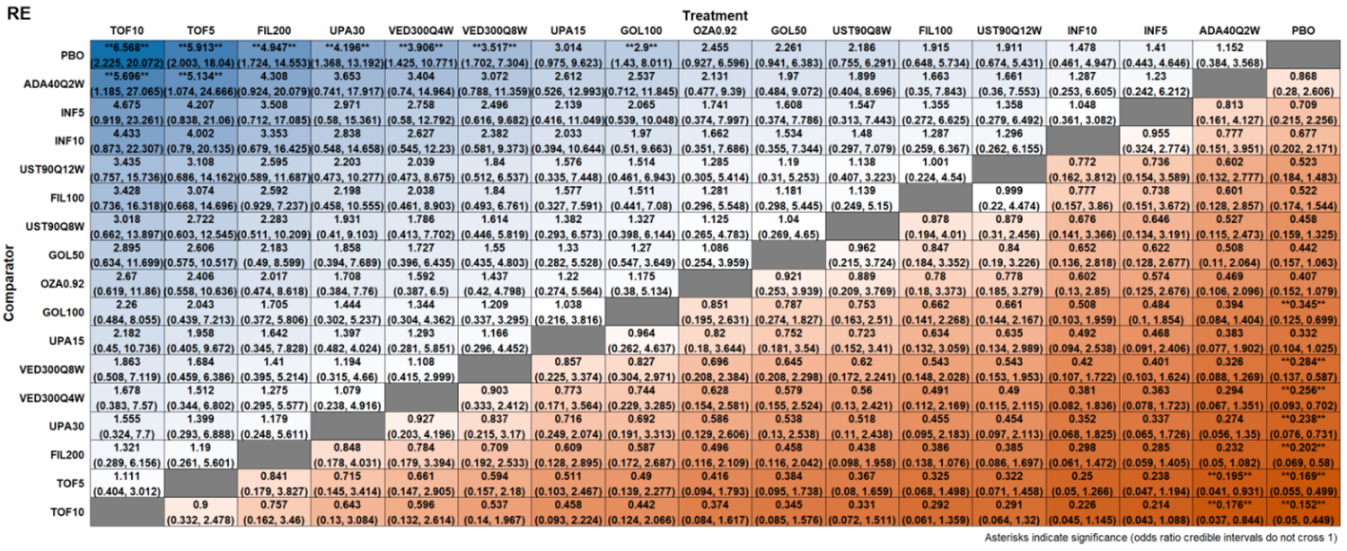


Figure 10. OR league table for bio-naïve endoscopic improvement induction (RE model)


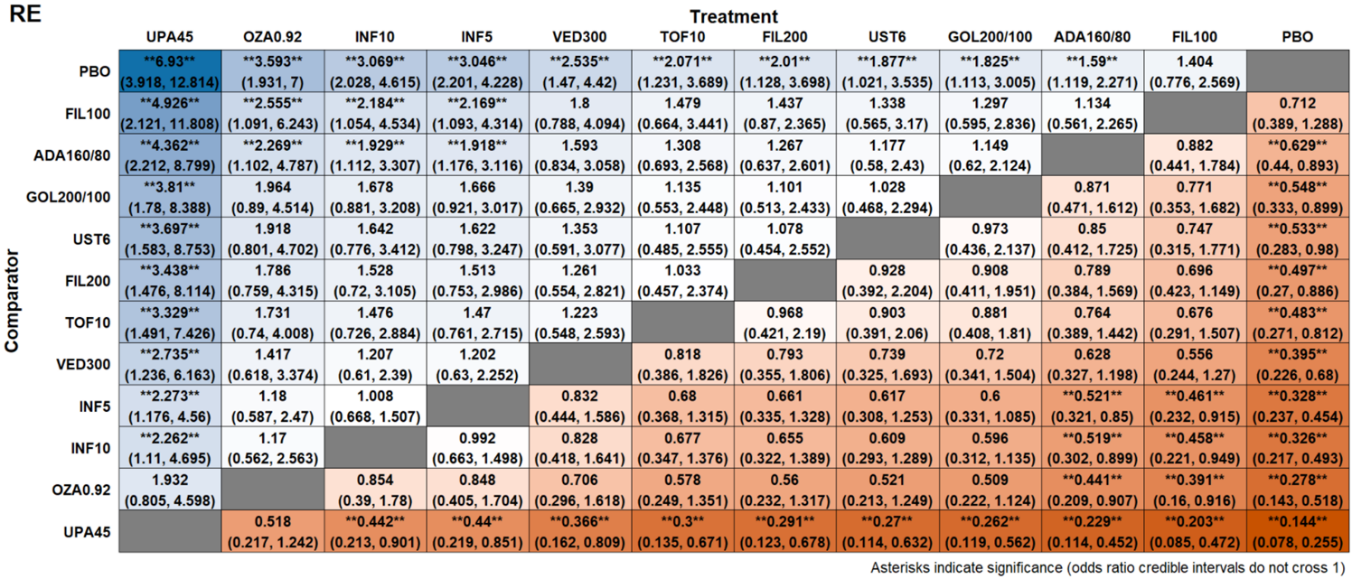


Figure 11. OR league table for bio-naïve endoscopic improvement maintenance (RE model)


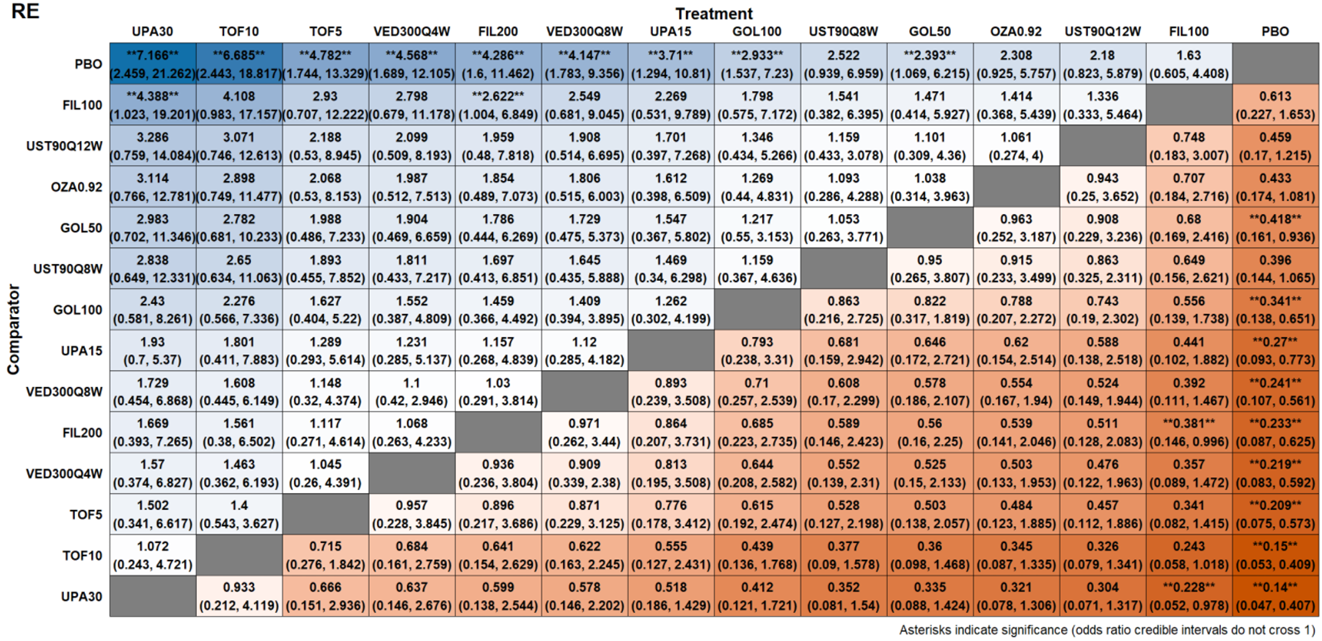


## Appendix 9: Bio-exposed efficacy OR league tables

Figure 12. OR league table for bio-exposed clinical response induction (RE model)


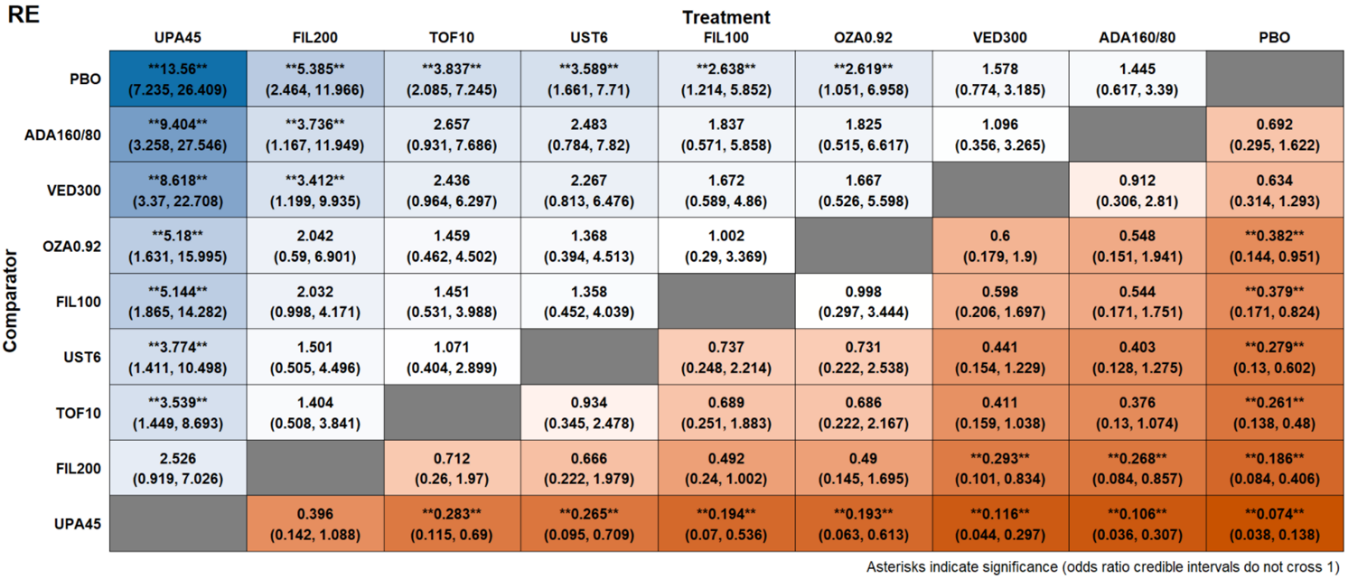


Figure 13. OR league table for bio-exposed clinical response maintenance (RE model)


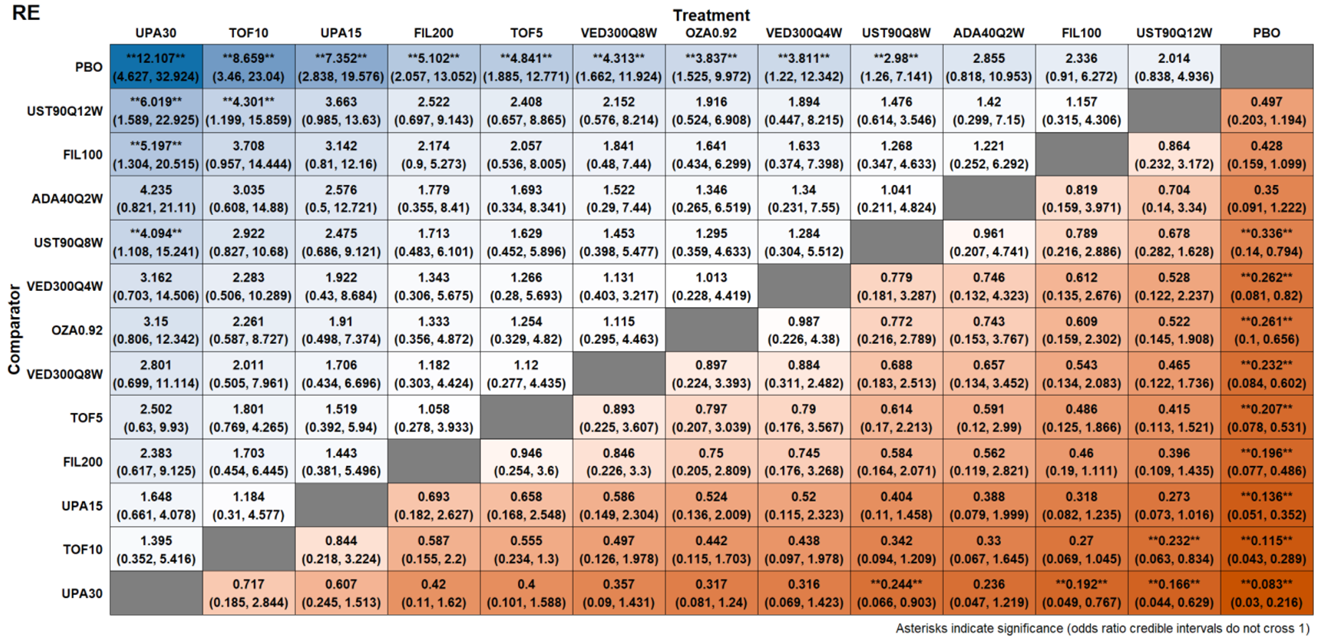


Figure 14. OR league table for bio-exposed clinical remission induction (RE model)


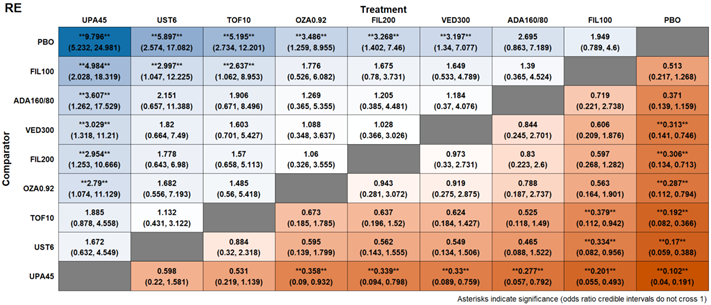


Figure 15. OR league table for bio-exposed clinical remission maintenance (RE model)


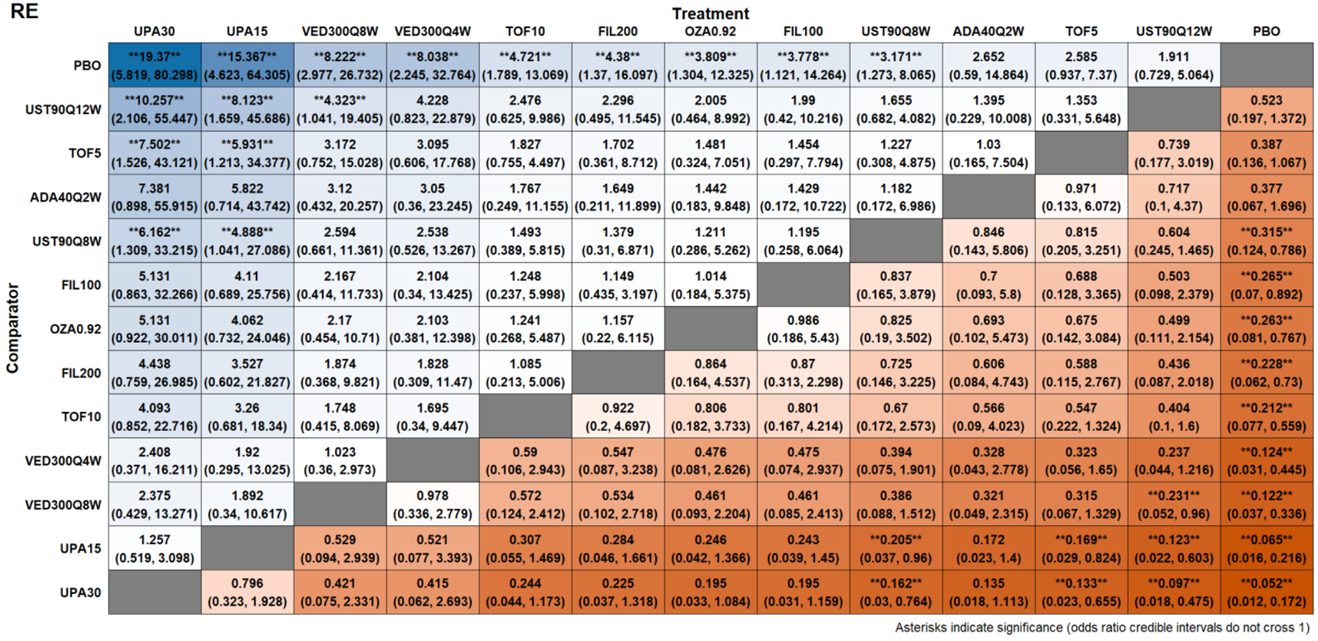


Figure 16. OR league table for bio-exposed endoscopic improvement induction (RE model)


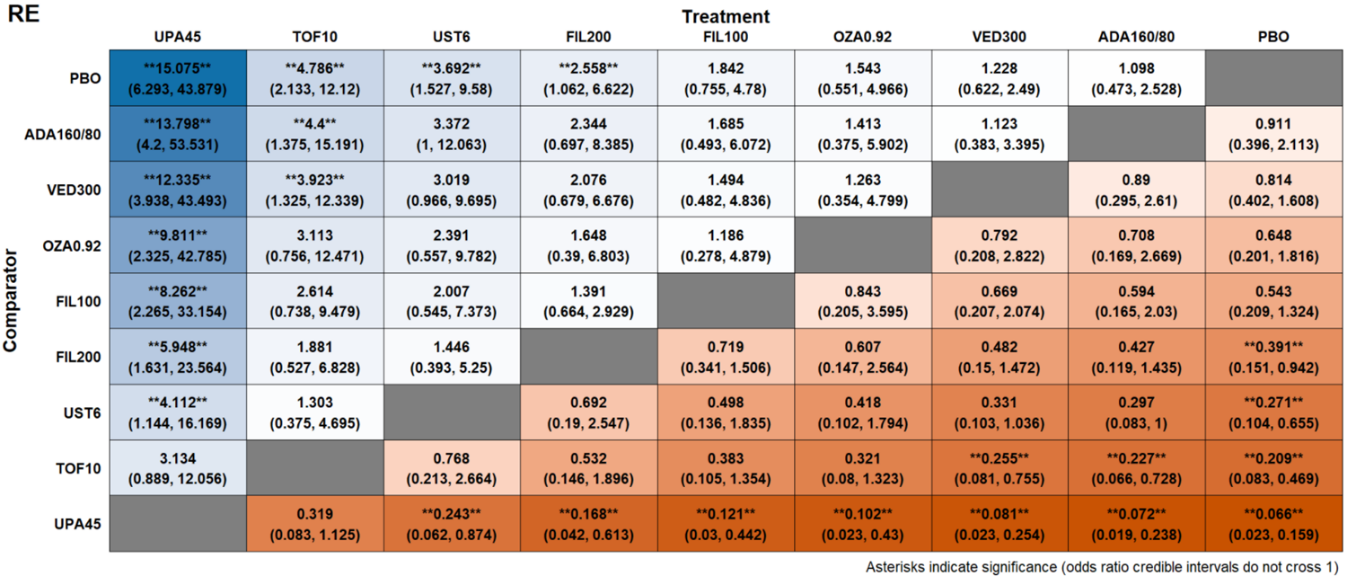


Figure 17. OR league table for bio-exposed endoscopic improvement maintenance (RE model)


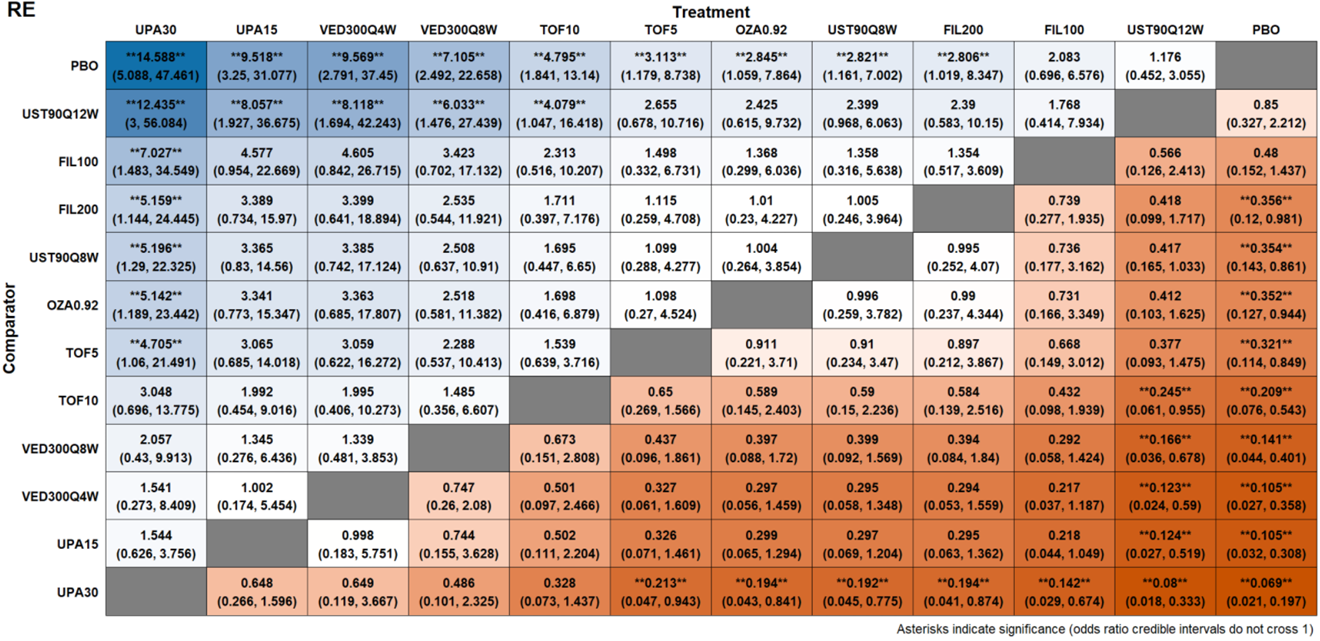


## Appendix 10: Overall safety OR league tables

Figure 18. OR league table for overall induction all AEs (REA model)


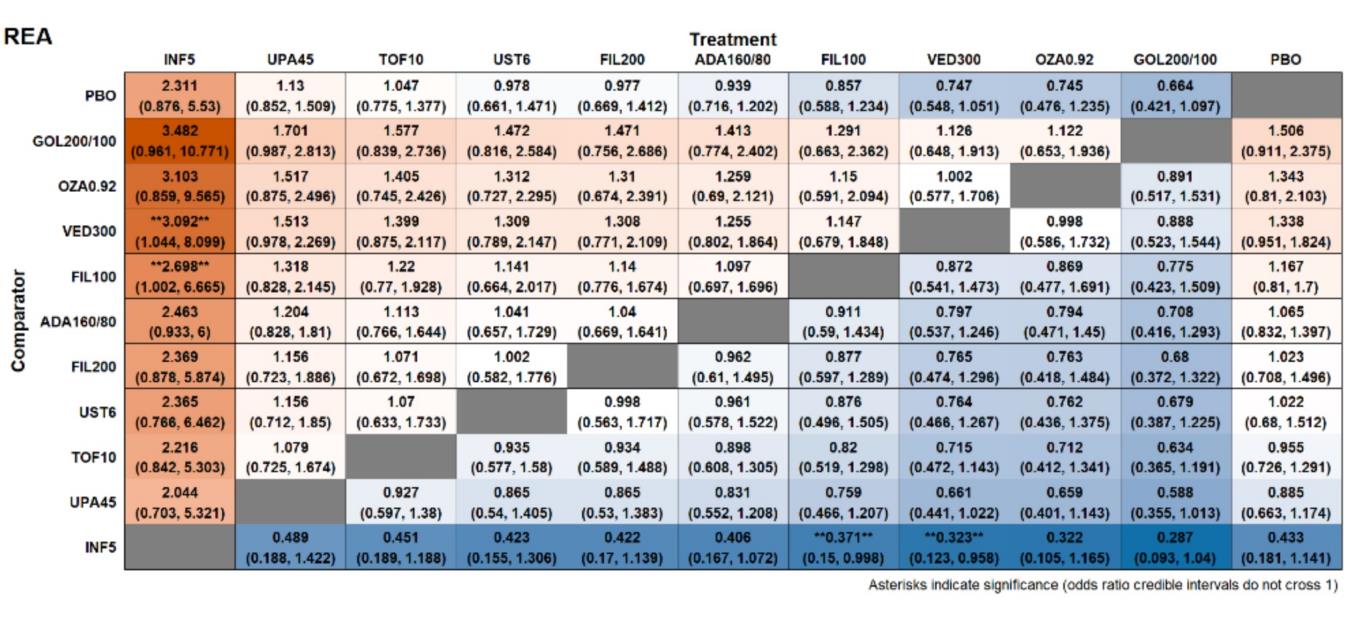


Figure 19. OR league table for overall maintenance all AEs (RE model)


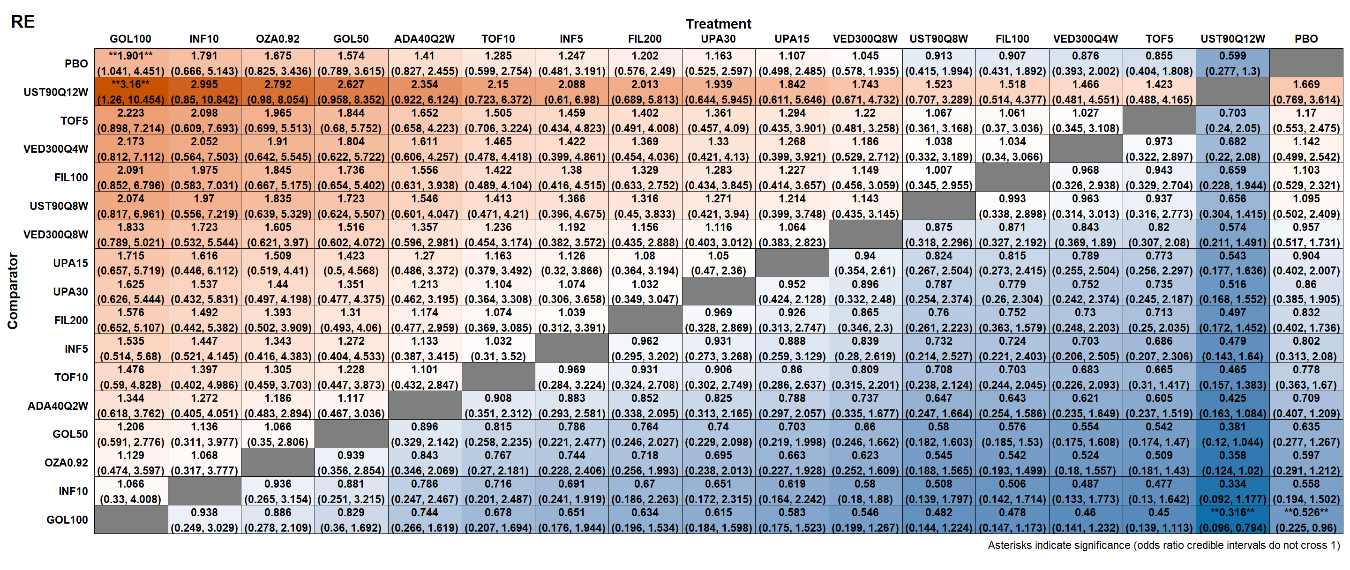


Figure 20. OR league table for overall induction discontinuation due to AEs (RE model)


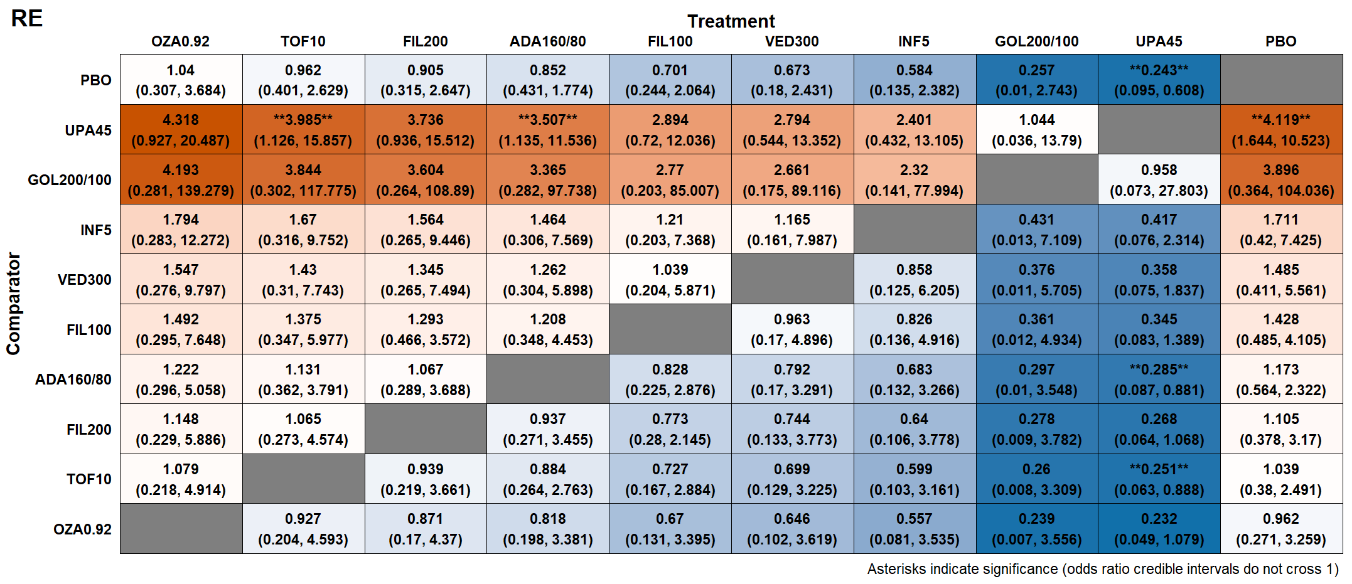


Figure 21. OR league table for overall maintenance discontinuation due to AEs (RE model)


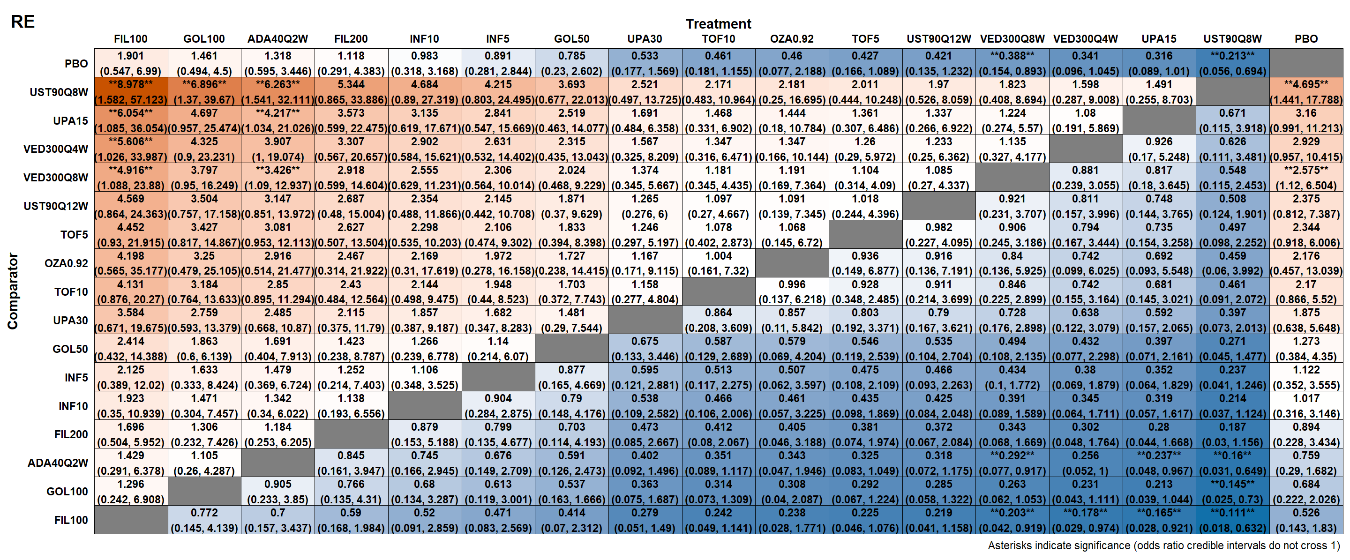


Figure 22. OR league table for overall induction serious AEs (RE model)


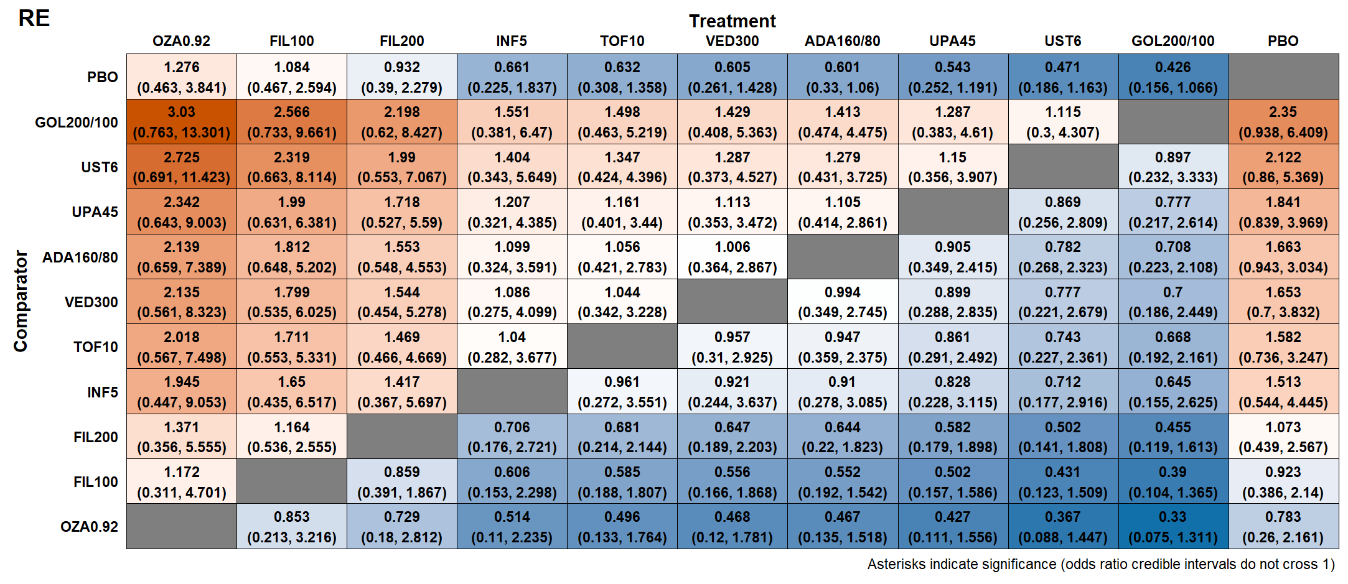


Figure 23. OR league table for overall maintenance serious AEs (RE model)


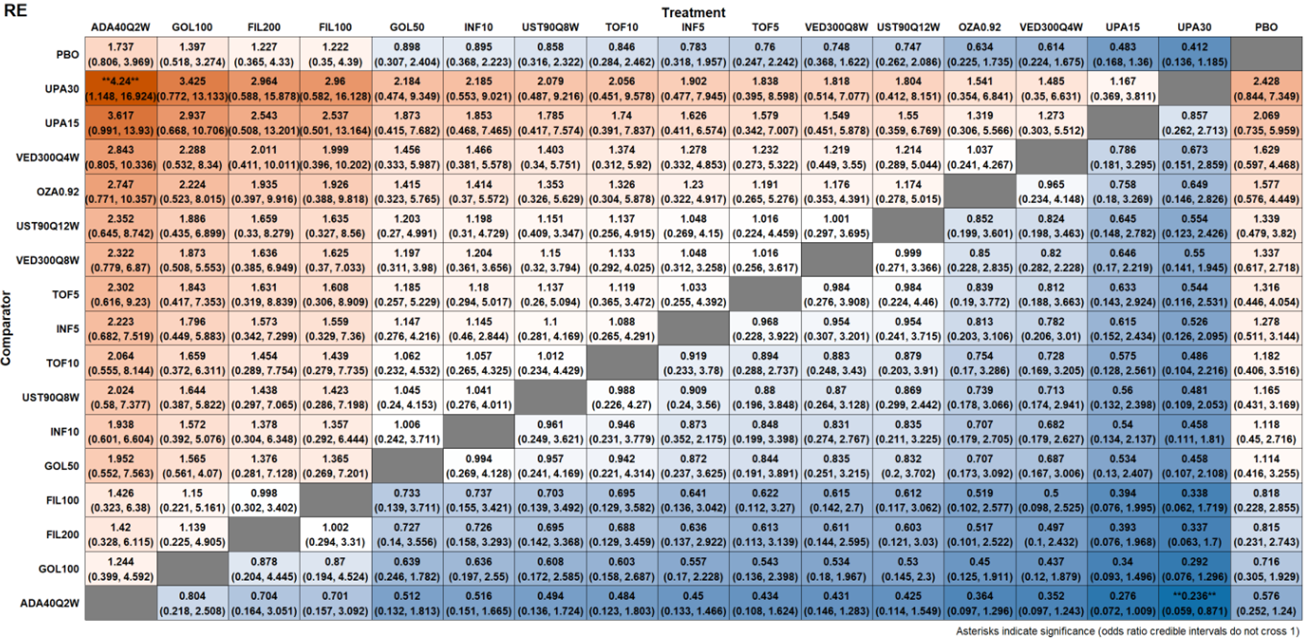


Figure 24. OR league table for overall induction serious infections (RE model)


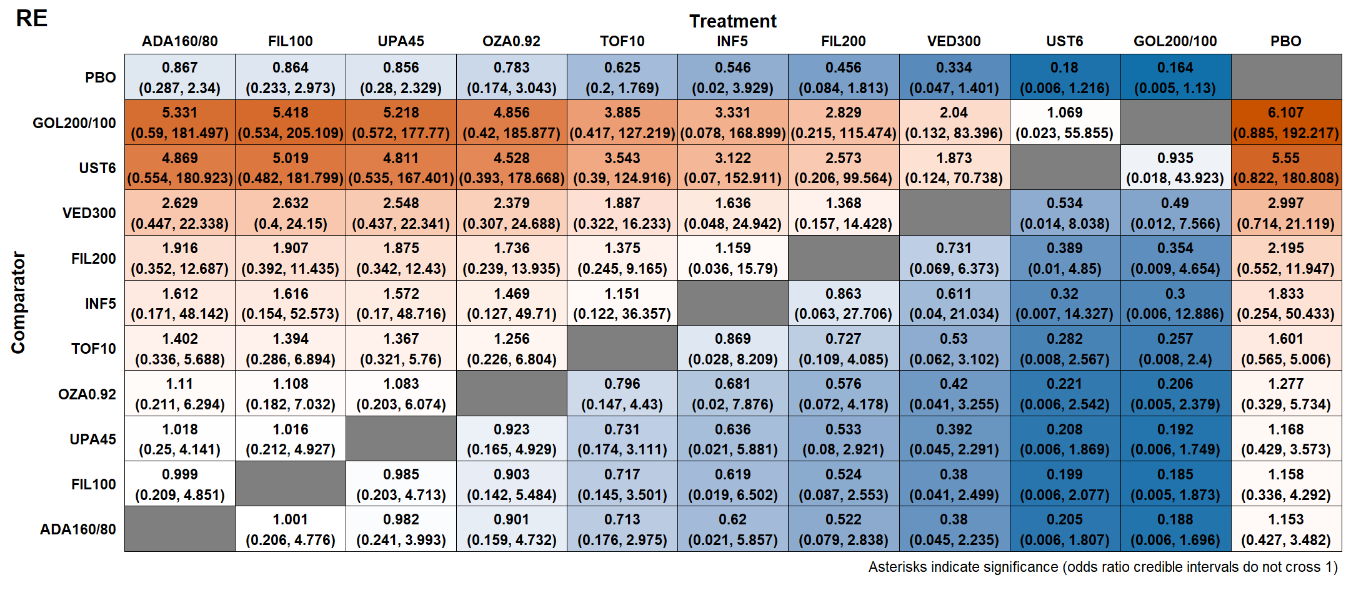


Figure 25. OR league table for overall maintenance serious infections (RE model)


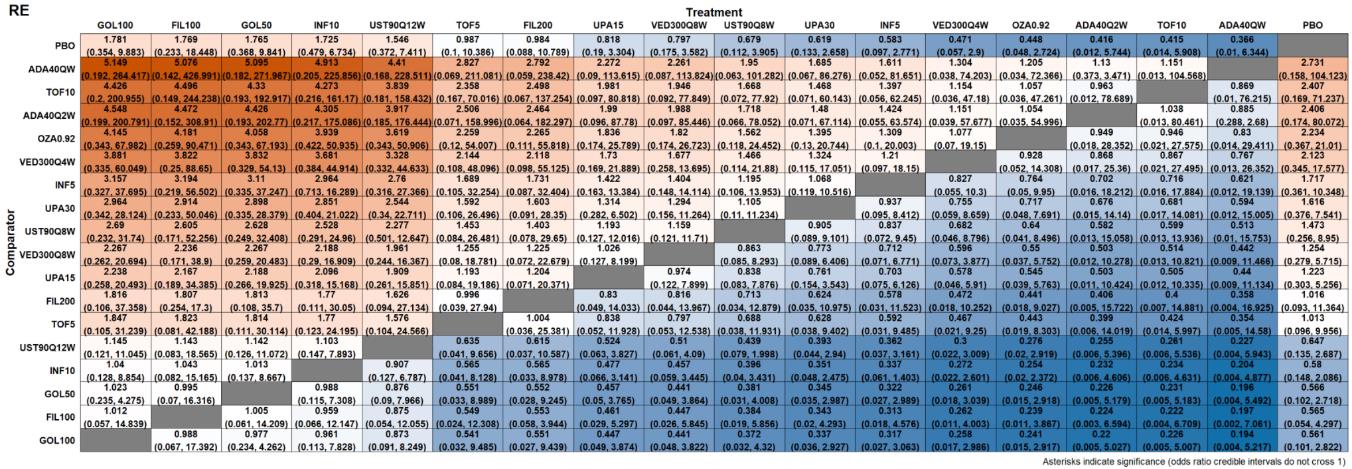


## Appendix 11: Scatter plots

Figure 26. Scatter plots of estimated absolute rates for selected outcome combinations


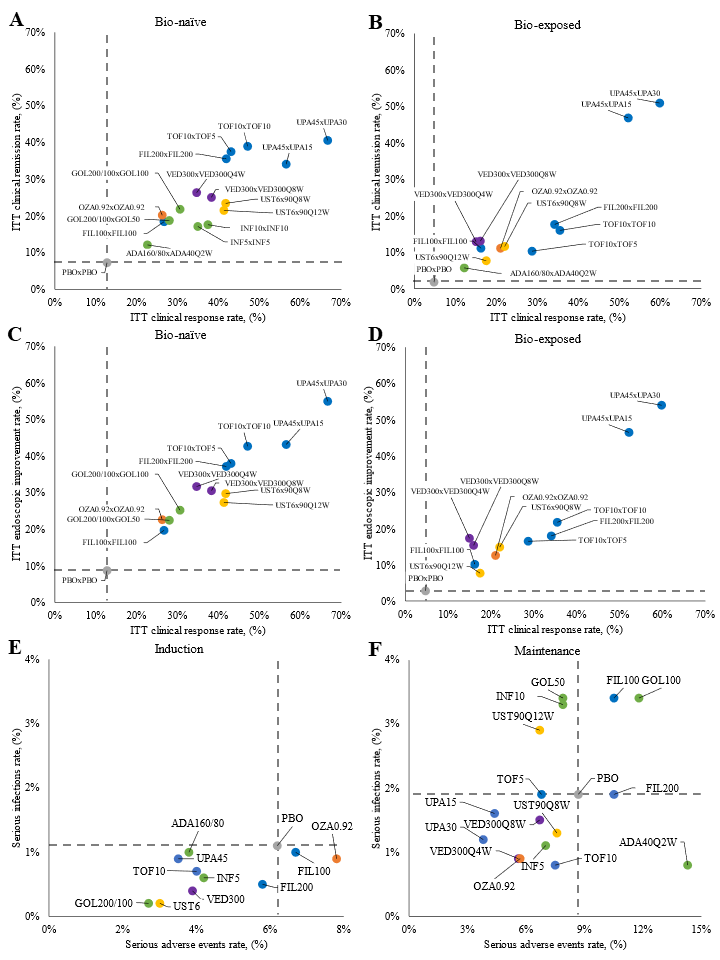


Note: Colouring indicates drug class: blue – JAKi; green – TNFi; orange – S1P receptor modulator; purple – anti-integrin; yellow – anti-IL12/23.

A: ITT clinical response vs ITT clinical remission in bio-naïve populations; B: ITT clinical response vs ITT clinical remission in bio-exposed populations; C: ITT clinical response vs ITT endoscopic improvement in bio-naïve populations; D: ITT clinical response vs ITT endoscopic improvement in bio-exposed populations; E: Induction serious adverse events vs induction serious infections in overall populations; F: Maintenance serious adverse events vs maintenance serious infections in overall populations.

Figure 27: Relative treatment rankings per SUCRA estimates for induction clinical response by biologic exposure status vs induction discontinuation due to AEs overall†


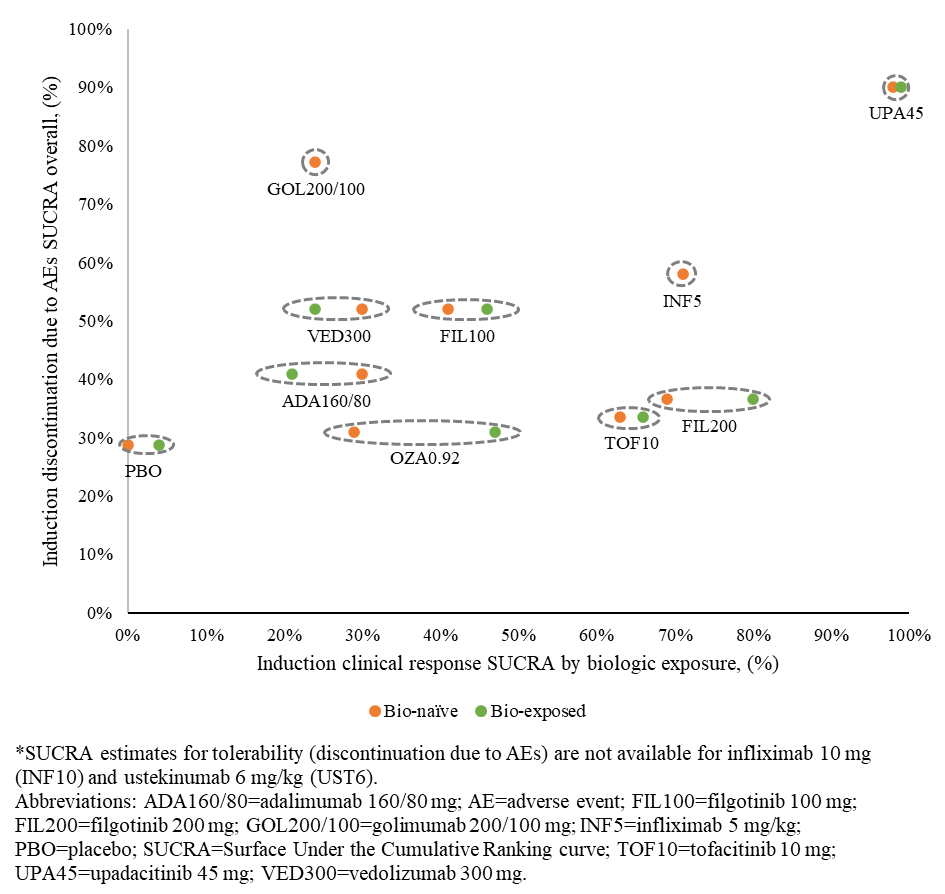


† SUCRA estimates for tolerability (discontinuation due to AEs) are available in the overall population only, and not available for infliximab 10 mg and ustekinumab 6 mg/kg, while SUCRA estimates for efficacy (induction clinical response) are available in bio-naïve and -exposed populations.

**Abbreviations**: ADA160/80=adalimumab 160/80 mg; AE=adverse event; FIL100=filgotinib 100 mg; FIL200=filgotinib 200 mg; GOL200/100=golimumab 200/100 mg; INF5=infliximab 5 mg/kg; PBO=placebo; SUCRA=surface under the cumulative ranking curve; TOF10=tofacitinib 10 mg; UPA45=upadacitinib 45 mg; VED300=vedolizumab 300 mg.

Figure 28: Relative treatment rankings per SUCRA estimates for induction clinical remission by biologic exposure status vs serious AEs overall†


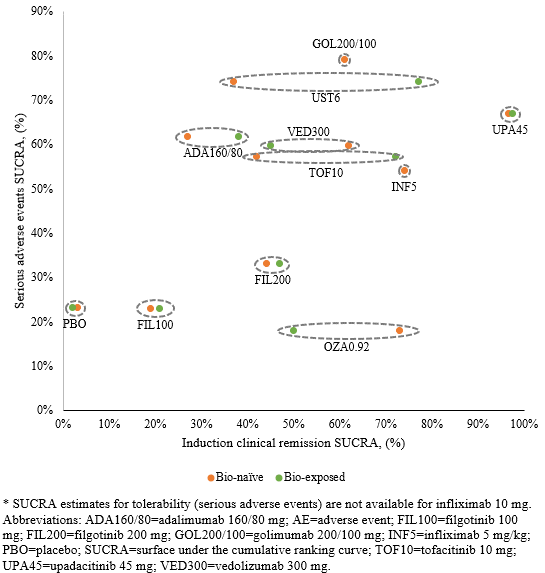


† SUCRA estimates for tolerability (serious adverse events) are available in the overall population only, and not available for infliximab 10 mg, while SUCRA estimates for efficacy (induction clinical remission) are available in bio-naïve and -exposed populations.

**Abbreviations**: ADA160/80=adalimumab 160/80 mg; AE=adverse event; FIL100=filgotinib 100 mg; FIL200=filgotinib 200 mg; GOL200/100=golimumab 200/100 mg; INF5=infliximab 5 mg/kg; PBO=placebo; SUCRA=surface under the cumulative ranking curve; TOF10=tofacitinib 10 mg; UPA45=upadacitinib 45 mg; VED300=vedolizumab 300 mg.

## Appendix 12: RD NMA results

Table 24. NMA of efficacy outcomes (clinical response, clinical remission, endoscopic improvement) in bio-naïve populations – RD sensitivity analysis†

| **Phase** | **Treatment** | **Clinical Response** | | | **Clinical Remission** | | | **Endoscopic Improvement** | | |
| --- | --- | --- | --- | --- | --- | --- | --- | --- | --- | --- |
|  |  | **RD  (vs PBO)** | **Absolute rate** | **SUCRA** | **RD  (vs PBO)** | **Absolute rate** | **SUCRA** | **RD  (vs PBO)** | **Absolute rate** | **SUCRA** |
| **Induction  (6-10 weeks post-baseline)** | Upadacitinib 45 mg QD | 0.4* | 79% (66%-92%) | 99% |  |  |  |  |  |  |
|  | Infliximab 5 mg | 0.3* | 66% (55%-78%) | 79% |  |  |  |  |  |  |
|  | Infliximab 10 mg | 0.3* | 66% (54%-78%) | 77% |  |  |  |  |  |  |
|  | Ustekinumab 6 mg/kg | 0.3* | 66% (52%-81%) | 77% |  |  |  |  |  |  |
|  | Ozanimod 0.92 mg | 0.2* | 59% (46%-72%) | 51% |  |  |  |  |  |  |
|  | Vedolizumab 300 mg | 0.2* | 59% (44%-73%) | 50% |  |  |  |  |  |  |
|  | Tofacitinib 10 mg BID | 0.2* | 58% (44%-72%) | 49% |  |  |  |  |  |  |
|  | Golimumab 200/100 mg | 0.2* | 56% (44%-69%) | 41% |  |  |  |  |  |  |
|  | Filgotinib 200 mg QD | 0.2* | 55% (41%-69%) | 39% |  |  |  |  |  |  |
|  | Adalimumab 160/80 mg | 0.2* | 51% (39%-63%) | 22% |  |  |  |  |  |  |
|  | Filgotinib 100 mg QD | 0.1* | 48% (34%-62%) | 16% |  |  |  |  |  |  |
|  | PBO | 0.0 | 36% (26%-45%) | 0% |  |  |  |  |  |  |
| **Maintenance**‡ **(40-54 weeks post-induction response)** | Upadacitinib 30 mg QD | 0.5* | 87% (60%-100%) | 98% | 0.3* | 50% (29%-71%) | 79% | 0.4* | 68% (52%-83%) | 94% |
|  | Tofacitinib 10 mg BID | 0.4* | 76% (50%-100%) | 87% | 0.3* | 54% (35%-72%) | 88% | 0.4* | 61% (48%-74%) | 83% |
|  | Vedolizumab 300 mg Q8W | 0.4* | 72% (47%-98%) | 80% | 0.3* | 48% (29%-66%) | 73% | 0.3* | 57% (43%-71%) | 72% |
|  | Upadacitinib 15 mg QD | 0.4* | 72% (45%-99%) | 78% | 0.2* | 42% (21%-63%) | 57% | 0.3* | 53% (36%-69%) | 59% |
|  | Tofacitinib 5 mg BID | 0.3* | 68% (42%-93%) | 70% | 0.3* | 51% (33%-70%) | 83% | 0.3* | 53% (40%-66%) | 60% |
|  | Filgotinib 200 mg QD | 0.3* | 65% (39%-90%) | 62% | 0.3* | 52% (33%-70%) | 83% | 0.3* | 56% (42%-70%) | 69% |
|  | Vedolizumab 300 mg Q4W | 0.3* | 65% (38%-91%) | 61% | 0.3* | 50% (29%-70%) | 78% | 0.3* | 59% (43%-74%) | 77% |
|  | Ustekinumab 90 mg Q8W | 0.2* | 61% (34%-87%) | 52% | 0.2* | 39% (18%-60%) | 50% | 0.2* | 47% (31%-62%) | 42% |
|  | Ustekinumab 90 mg Q12W | 0.2* | 61% (34%-87%) | 51% | 0.1* | 36% (16%-56%) | 40% | 0.2* | 43% (28%-59%) | 32% |
|  | Infliximab 10 mg/kg Q8W | 0.2* | 58% (29%-86%) | 45% | 0.1 | 28% (6%-50%) | 22% | NA | NA | NA |
|  | Golimumab 100 mg Q4W | 0.2* | 58% (34%-82%) | 44% | 0.2* | 38% (21%-56%) | 47% | 0.2* | 45% (34%-56%) | 37% |
|  | Infliximab 5 mg/kg Q8W | 0.2 | 54% (26%-82%) | 34% | 0.1 | 27% (5%-49%) | 19% | NA | NA | NA |
|  | Golimumab 50 mg Q4W | 0.2* | 54% (29%-78%) | 31% | 0.1* | 34% (17%-52%) | 35% | 0.2* | 42% (30%-54%) | 27% |
|  | Ozanimod 0.92 mg QD | 0.1* | 51% (26%-75%) | 24% | 0.2* | 40% (22%-58%) | 51% | 0.2* | 44% (32%-56%) | 34% |
|  | Filgotinib 100 mg QD | 0.1 | 48% (22%-74%) | 20% | 0.1 | 31% (13%-49%) | 26% | 0.1 | 34% (21%-47%) | 12% |
|  | Adalimumab 40 mg Q2W | 0.1 | 43% (15%-70%) | 12% | 0.0 | 24% (2%-45%) | 13% | NA | NA | NA |
|  | PBO | 0.0 | 37% (14%-59%) | 2% | 0.0 | 21% (6%-36%) | 5% | 0.0 | 25% (19%-31%) | 1% |

* Denotes statistical significance (RD 95% CrI excludes 0).

† Results displayed for FE and ordered in descending (best to worst rank) SUCRA values for clinical response.

‡ Outcomes of maintenance treatment among induction responders.

**Abbreviations**: BID=twice daily; CrI=credible interval; NA=not available; NMA=network meta-analysis; RD=risk difference; PBO=placebo; Q#W=every # week; QD=once daily; FE=fixed effects model; SUCRA=surface under the cumulative ranking curve.

Table 25. NMA of efficacy outcomes (clinical response, clinical remission, and endoscopic improvement) in bio-exposed populations – RD sensitivity analysis†

| **Phase** | **Treatment** | **Clinical Response** | | | **Clinical Remission** | | | **Endoscopic Improvement** | | |
| --- | --- | --- | --- | --- | --- | --- | --- | --- | --- | --- |
|  |  | **RD  (vs PBO)** | **Absolute rate** | **SUCRA** | **RD  (vs PBO)** | **Absolute rate** | **SUCRA** | **RD  (vs PBO)** | **Absolute rate** | **SUCRA** |
| **Induction  (6-10 weeks post-baseline)** | Upadacitinib 45 mg QD | 0.5* | 76% (63%-89%) | 100% |  |  |  | 0.3* | 38% (29%-47%) | 100% |
|  | Filgotinib 200 mg QD | 0.4* | 57% (43%-71%) | 83% |  |  |  | 0.1* | 19% (10%-28%) | 57% |
|  | Tofacitinib 10 mg BID | 0.3* | 51% (37%-64%) | 69% |  |  |  | 0.2* | 26% (17%-34%) | 82% |
|  | Ustekinumab 6 mg/kg | 0.3* | 51% (36%-66%) | 70% |  |  |  | 0.1* | 24% (14%-34%) | 75% |
|  | Ozanimod 0.92 mg | 0.2* | 40% (23%-56%) | 42% |  |  |  | 0.0 | 14% (2%-25%) | 33% |
|  | Filgotinib 100 mg QD | 0.2* | 40% (26%-53%) | 42% |  |  |  | 0.0 | 15% (6%-24%) | 36% |
|  | Vedolizumab 300 mg | 0.1 | 30% (15%-45%) | 21% |  |  |  | 0.0 | 14% (1%-26%) | 32% |
|  | Adalimumab 160/80 mg | 0.1 | 30% (13%-46%) | 20% |  |  |  | 0.0 | 12% (%-26%) | 24% |
|  | PBO | 0.0 | 22% (11%-33%) | 2% |  |  |  | 0.0 | 10% (3%-17%) | 11% |
| **Maintenance**‡ **(40-54 weeks post-induction response)** | Upadacitinib 30 mg QD | 0.5* | 75% (59%-89%) | 97% | 0.4* | 50% (38%-63%) | 96% | 0.5* | 61% (46%-74%) | 96% |
|  | Tofacitinib 10 mg BID | 0.4* | 66% (51%-80%) | 86% | 0.2* | 34% (22%-46%) | 66% | 0.3* | 41% (27%-55%) | 63% |
|  | Upadacitinib 15 mg QD | 0.4* | 64% (47%-79%) | 80% | 0.4* | 45% (32%-58%) | 89% | 0.4* | 50% (36%-64%) | 81% |
|  | Filgotinib 200 mg QD | 0.4* | 58% (42%-73%) | 68% | 0.1* | 24% (14%-35%) | 37% | 0.1* | 28% (15%-42%) | 33% |
|  | Tofacitinib 5 mg BID | 0.3* | 52% (37%-67%) | 52% | 0.1* | 22% (10%-34%) | 30% | 0.2* | 32% (18%-45%) | 40% |
|  | Vedolizumab 300 mg Q8W | 0.3* | 51% (33%-69%) | 51% | 0.3* | 37% (24%-50%) | 73% | 0.3* | 47% (30%-63%) | 74% |
|  | Ozanimod 0.92 mg QD | 0.3* | 52% (35%-69%) | 54% | 0.2* | 28% (15%-41%) | 47% | 0.2* | 33% (18%-48%) | 44% |
|  | Vedolizumab 300 mg Q4W | 0.3* | 48% (27%-68%) | 42% | 0.3* | 37% (20%-54%) | 72% | 0.4* | 52% (33%-70%) | 83% |
|  | Ustekinumab 90 mg Q8W | 0.3* | 48% (32%-64%) | 44% | 0.2* | 31% (18%-43%) | 56% | 0.2* | 36% (21%-51%) | 51% |
|  | Adalimumab 40 mg Q2W | 0.2 | 42% (19%-64%) | 31% | 0.1 | 20% (2%-38%) | 27% | NA | NA | NA |
|  | Filgotinib 100 mg QD | 0.2* | 39% (23%-56%) | 23% | 0.1* | 22% (11%-34%) | 31% | 0.1 | 24% (10%-38%) | 22% |
|  | Ustekinumab 90 mg Q12W | 0.2* | 39% (22%-56%) | 22% | 0.1 | 20% (7%-33%) | 25% | 0.0 | 17% (3%-32%) | 10% |
|  | PBO | 0.0 | 22% (14%-30%) | 1% | 0.0 | 9% (5%-13%) | 2% | 0.0 | 14% (8%-21%) | 4% |

* Denotes statistical significance (RD 95% CrI excludes 0).

† Results displayed for FE and ordered in descending (best to worst rank) SUCRA values.

‡ Outcomes of maintenance treatment among induction responders.

**Abbreviations**: BID=twice daily; CrI=credible interval; NA=not available; NMA=network meta-analysis; RD=risk difference; PBO=placebo; Q#W=every # week; QD=once daily; FE=fixed effects model; SUCRA=surface under the cumulative ranking curve.

Figure 29. Bio-naïve intent-to-treat (ITT) maintenance efficacy adjusted by induction response – RD sensitivity analysis†


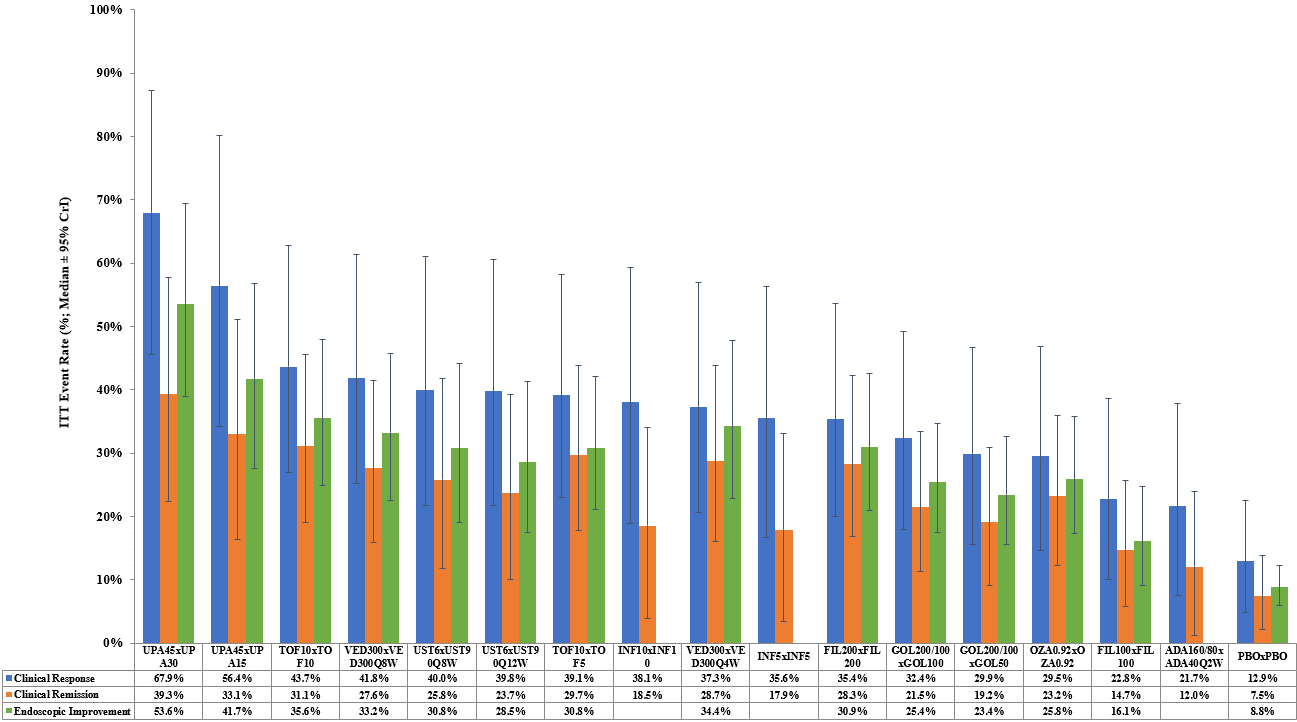


† Absolute rate samples for induction response (per FE) were multiplied by absolute rates samples for each maintenance efficacy (per FE) to obtain ITT rates; median ± 95% CrI rates are presented; treatments are ordered by descending ITT rates for clinical response.

**Abbreviations**: CrI=credible interval; ITT=intent-to-treat; PBO=placebo; FE=fixed effects model.

Figure 30. Bio-exposed intent-to-treat (ITT) maintenance efficacy adjusted by induction response – RD sensitivity analysis†


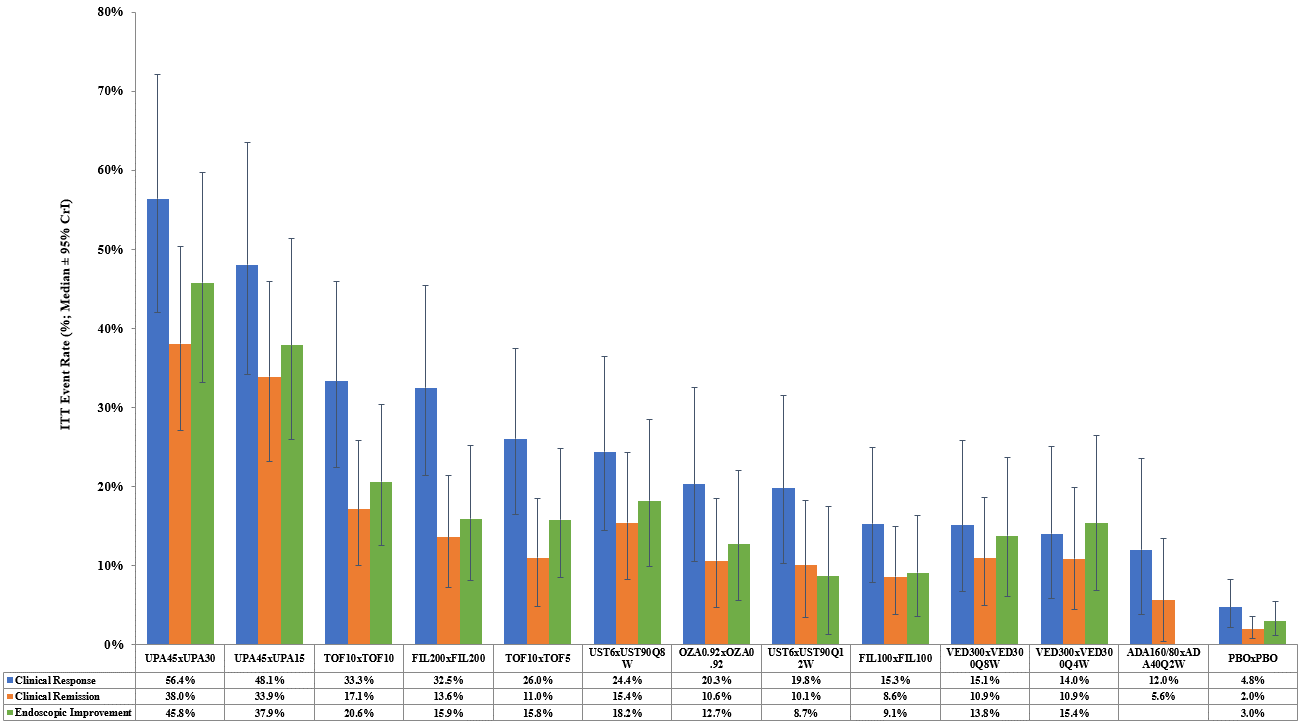


† Absolute rate samples for induction response (per FE) were multiplied by absolute rates samples for each maintenance efficacy (per FE) to obtain ITT rates; median ± 95% CrI rates are presented; treatments are ordered by descending ITT rates for clinical response.

**Abbreviations**: CrI=credible interval; ITT=intent-to-treat; PBO=placebo; FE=fixed effects model.

Table 26. NMA of safety outcomes (all AEs and discontinuation due to AEs) in overall populations – RD sensitivity analysis†

| **Phase** | **Treatment** | **All AEs** | | | **Discontinuation due to AEs** | | |
| --- | --- | --- | --- | --- | --- | --- | --- |
|  |  | **RD  (vs PBO)** | **Absolute rate** | **SUCRA** | **RD  (vs PBO)** | **Absolute rate** | **SUCRA** |
| **Induction  (6-10 weeks post-baseline)** | Upadacitinib 45 mg QD |  |  |  | 0.0* | 0.0% (0.0%-2.5%) | 92.9% |
|  | Vedolizumab 300 mg |  |  |  | 0.0* | 1.1% (0.0%-4.3%) | 78.8% |
|  | Infliximab 5 mg |  |  |  | 0.0 | 1.5% (0.0%-8.5%) | 64.2% |
|  | Filgotinib 100 mg QD |  |  |  | 0.0 | 2.7% (0.0%-6.0%) | 57.0% |
|  | Adalimumab 160/80 mg |  |  |  | 0.0 | 3.3% (0.1%-6.5%) | 45.1% |
|  | Golimumab 200/100 mg |  |  |  | 0.0 | 3.7% (1.4%-5.9%) | 39.1% |
|  | Filgotinib 200 mg QD |  |  |  | 0.0 | 3.7% (0.0%-7.2%) | 37.3% |
|  | Tofacitinib 10 mg BID |  |  |  | 0.0 | 4.1% (0.4%-7.2%) | 32.4% |
|  | Ozanimod 0.92 mg |  |  |  | 0.0 | 4.2% (0.5%-7.5%) | 30.5% |
|  | PBO |  |  |  | 0.0 | 4.3% (2.6%-6.0%) | 22.8% |
|  | Ustekinumab 6 mg/kg |  |  |  | NA | NA | NA |
| **Maintenance**‡ **(40-54 weeks post-induction response)** | Ustekinumab 90 mg Q12W | -0.1* | 56% (39%-73%) | 95.7% |  |  |  |
|  | Tofacitinib 5 mg BID | 0.0 | 62% (45%-79%) | 77.2% |  |  |  |
|  | Filgotinib 100 mg QD | 0.0 | 63% (45%-81%) | 73.2% |  |  |  |
|  | Vedolizumab 300 mg Q4W | 0.0 | 63% (46%-80%) | 72.8% |  |  |  |
|  | Ustekinumab 90 mg Q8W | 0.0 | 64% (47%-81%) | 70.0% |  |  |  |
|  | PBO | 0.0 | 65% (51%-80%) | 65.0% |  |  |  |
|  | Vedolizumab 300 mg Q8W | 0.0 | 66% (50%-82%) | 61.0% |  |  |  |
|  | Upadacitinib 15 mg QD | 0.0 | 67% (50%-85%) | 53.4% |  |  |  |
|  | Infliximab 5 mg/kg Q8W | 0.0 | 68% (51%-85%) | 50.6% |  |  |  |
|  | Upadacitinib 30 mg QD | 0.0 | 68% (51%-85%) | 48.6% |  |  |  |
|  | Filgotinib 200 mg QD | 0.0 | 70% (52%-87%) | 40.8% |  |  |  |
|  | Tofacitinib 10 mg BID | 0.0 | 70% (53%-86%) | 39.7% |  |  |  |
|  | Infliximab 10 mg/kg Q8W | 0.1 | 71% (54%-88%) | 32.4% |  |  |  |
|  | Adalimumab 40 mg Q2W | 0.1* | 72% (56%-87%) | 29.6% |  |  |  |
|  | Golimumab 50 mg Q4W | 0.1 | 74% (57%-92%) | 20.5% |  |  |  |
|  | Golimumab 100 mg Q4W | 0.1* | 77% (60%-94%) | 10.2% |  |  |  |
|  | Ozanimod 0.92 mg QD | 0.1* | 78% (61%-95%) | 9.4% |  |  |  |
|  | Adalimumab 40 mg QW | NA | NA | NA |  |  |  |

* Denotes statistical significance (RD 95% CrI excludes 0).

† Results displayed for FE and ordered in descending (best to worst rank) SUCRA values.

‡ Outcomes of maintenance treatment among induction responders.

**Abbreviations**: BID=twice daily; CrI=credible interval; NA=not available; NMA=network meta-analysis; RD=risk difference; PBO=placebo; Q#W=every # week; QD=once daily; FE=fixed effects model; SUCRA=surface under the cumulative ranking curve.

## Appendix 13: Numbers needed to treat or harm

Table 27. Number needed to treat of UPA vs comparators using ITT maintenance efficacy adjusted by induction response†

| **Setting** | **Treatment** | **Induction (Response)** | **Maintenance** | **Clinical Response** | | **Clinical Remission** | | **Endoscopic Improvement** | |
| --- | --- | --- | --- | --- | --- | --- | --- | --- | --- |
|  |  |  |  | **UPA45xUPA30** | **UPA45xUPA15** | **UPA45xUPA30** | **UPA45xUPA15** | **UPA45xUPA30** | **UPA45xUPA15** |
| **Bio-naïve population** | Tofacitinib | 10 mg BID | 10 mg BID | 5.15* | 7.43 | 4.67 | -5.51 | 6.24 | 4.04 |
|  | Tofacitinib | 10 mg BID | 5 mg BID | 4.32* | 6.49 | 5.26 | -5.06 | 5.35 | 6.06 |
|  | Filgotinib | 200 mg QD | 200 mg QD | 4.13* | 6.10 | 5.49 | -4.03 | 5.21 | 5.94 |
|  | Ustekinumab | 6 mg/kg^1^ | 90 mg Q8W | 4.11* | 6.05 | 5.21 | 6.12 | 4.02 | 5.80 |
|  | Ustekinumab | 6 mg/kg^1^ | 90 mg Q12W | 4.04* | 5.96 | 4.95 | 6.02 | 3.73* | 5.48 |
|  | Vedolizumab | 300 mg^2^ | 300 mg Q8W | 3.60* | 5.28 | 5.60 | 6.50 | 4.10* | 6.05 |
|  | Infliximab | 10 mg/kg^2^ | 10 mg/kg Q8W | 3.53* | 5.11 | 4.35 | 5.41 | NA | NA |
|  | Infliximab | 5 mg/kg^2^ | 5 mg/kg Q8W | 3.26* | 4.66 | 4.29 | 5.37 | NA | NA |
|  | Vedolizumab | 300 mg^2^ | 300 mg Q4W | 3.20* | 4.54 | 5.62 | 6.16 | 4.26 | 6.08 |
|  | Golimumab | 200/100 mg^3^ | 100 mg Q4W | 2.84* | 3.92* | 4.92 | 6.08 | 3.41* | 5.13 |
|  | Golimumab | 200/100 mg^3^ | 50 mg Q4W | 2.66* | 3.60* | 4.47 | 5.68 | 3.16* | 4.70 |
|  | Filgotinib | 100 mg QD | 100 mg QD | 2.59* | 3.48* | 4.47 | 5.58 | 2.95* | 4.30 |
|  | Ozanimod | 0.92 mg^4^ | 0.92 mg QD | 2.56* | 3.42* | 4.73 | 5.95 | 3.18* | 4.75 |
|  | Adalimumab | 160/80 mg^5^ | 40 mg Q2W | 2.35* | 3.07* | 3.63* | 4.60 | NA | NA |
|  | PBO |  |  | 1.88* | 2.31* | 3.02* | 3.75* | 2.18* | 2.94* |
| **Bio-exposed population** | Tofacitinib | 10 mg BID | 10 mg BID | 4.09* | 5.31 | 2.93* | 3.30* | 3.14* | 3.96 |
|  | Filgotinib | 200 mg QD | 200 mg QD | 3.90* | 5.03 | 3.06* | 3.44 | 2.86* | 3.54* |
|  | Tofacitinib | 10 mg BID | 5 mg BID | 3.28* | 4.22 | 2.52* | 2.81* | 2.74* | 3.38* |
|  | Ustekinumab | 6 mg/kg^1^ | 90 mg Q8W | 2.70* | 3.37* | 2.59* | 2.90* | 2.62* | 3.23* |
|  | Ozanimod | 0.92 mg^4^ | 0.92 mg QD | 2.64* | 3.28* | 2.58* | 2.88* | 2.48* | 3.02* |
|  | Ustekinumab | 6 mg/kg^1^ | 90 mg Q12W | 2.41* | 2.94* | 2.35* | 2.61* | 2.20* | 2.62* |
|  | Filgotinib | 100 mg QD | 100 mg QD | 2.35* | 2.84* | 2.59* | 2.88* | 2.34* | 2.82* |
|  | Vedolizumab | 300 mg^2^ | 300 mg Q8W | 2.34* | 2.82* | 2.70* | 3.02* | 2.65* | 3.26* |
|  | Vedolizumab | 300 mg^2^ | 300 mg Q4W | 2.29* | 2.75* | 2.69* | 3.01* | 2.79* | 3.46* |
|  | Adalimumab | 160/80 mg^5^ | 40 mg Q2W | 2.15* | 2.56* | 2.27* | 2.51* | NA | NA |
|  | PBO |  |  | 1.82* | 2.11* | 2.04* | 2.23* | 1.96* | 2.29* |

* Denotes associated NNT 95% CrI excludes absolute values below 1.

† NNTs were obtained by taking the inverse of the ITT rate of UPA minus that of the comparator for the setting; treatments are ordered by descending NNTs for clinical response by setting.

1. IV dose based on body weight (~6mg/kg) at Week 0.

2. IV doses at Week 0, 2, and 6.

3. SC 200mg at Week 0 and 100mg at Week 2.

4. Oral 0.23mg QD for 4 days, 0.46mg QD for 3 days, then 0.92mg QD starting on Day 8.

5. SC 160mg at Week 0 and 80mg at Week 2, then 40mg Q2W.

**Abbreviations**: BID=twice daily; CrI=credible interval; ITT=intent-to-treat; NNT=number needed to treat; IV=intravenous; NA=not available; PBO=placebo; Q#W=every # week; QD=once daily; SC=subcutaneous; UPA=upadacitinib; UPA45xUPA30=UPA 45 mg QD induction and UPA 30 mg QD maintenance; UPA45xUPA15=UPA 45 mg QD induction and UPA 15 mg QD maintenance.

Table 28. Number needed to harm of UPA vs comparators for safety events in overall populations†

| **Phase** | **Treatment** | **Any AEs** | | | **Discontinuation due to AEs** | | | **Serious AEs** | | | **Serious Infections** | | |
| --- | --- | --- | --- | --- | --- | --- | --- | --- | --- | --- | --- | --- | --- |
|  |  | **UPA45** | **UPA30** | **UPA15** | **UPA45** | **UPA30** | **UPA15** | **UPA45** | **UPA30** | **UPA15** | **UPA45** | **UPA30** | **UPA15** |
| **Induction  (6-10 weeks post-baseline)** | PBO | 23.45 | -- | -- | -32.50* | -- | -- | -35.09 | -- | -- | -153.66 | -- | -- |
|  | Tofacitinib 10 mg BID | 16.41 | -- | -- | -33.35* | -- | -- | -32.94 | -- | -- | 119.89 | -- | -- |
|  | Adalimumab 160/80 mg^3^ | 16.28 | -- | -- | -39.55* | -- | -- | -37.28 | -- | -- | -38.69 | -- | -- |
|  | Filgotinib 200 mg QD | 15.39 | -- | -- | -35.29 | -- | -- | -28.69 | -- | -- | 129.05 | -- | -- |
|  | Ustekinumab 6 mg/kg^5^ | 14.91 | -- | -- | NA | -- | -- | 32.63 | -- | -- | 125.40 | -- | -- |
|  | Filgotinib 100 mg QD | 12.74 | -- | -- | -47.67 | -- | -- | -25.36 | -- | -- | -24.28 | -- | -- |
|  | Vedolizumab 300 mg^4^ | 9.47 | -- | -- | -44.30 | -- | -- | -26.27 | -- | -- | 134.41 | -- | -- |
|  | Ozanimod 0.92 mg^2^ | 9.12 | -- | -- | -29.08 | -- | -- | -19.31 | -- | -- | 59.55 | -- | -- |
|  | Golimumab 200/100 mg^1^ | 7.45 | -- | -- | -7.30 | -- | -- | 38.72 | -- | -- | 126.68 | -- | -- |
|  | Infliximab 5 mg^4^ | -5.62 | -- | -- | -46.61 | -- | -- | -23.68 | -- | -- | 85.55 | -- | -- |
| **Maintenance**‡ **(40-54 weeks post-induction response)** | PBO | -- | 9.75 | 8.97 | -- | -26.87 | -20.85 | -- | -19.93 | -21.81 | -- | -83.56 | -77.95 |
|  | Tofacitinib 5 mg BID | -- | 7.24 | 7.07 | -- | 25.30 | -36.78 | -- | -20.18 | -18.84 | -- | -18.62 | -10.26 |
|  | Filgotinib 100 mg QD | -- | 7.24 | 6.99 | -- | -10.11 | -9.34* | -- | -12.90 | -12.89 | -- | -22.76 | -18.55 |
|  | Ustekinumab 90 mg Q8W | -- | 6.66 | 6.37 | -- | 33.33 | 39.16 | -- | -19.75 | -19.44 | -- | -19.68 | 29.38 |
|  | Vedolizumab 300 mg Q8W | -- | 6.55 | 5.68 | -- | 28.75 | -34.54 | -- | -24.62 | -24.12 | -- | -34.60 | 15.33 |
|  | Vedolizumab 300 mg Q4W | -- | 6.45 | 6.21 | -- | 29.16 | -20.28 | -- | -22.87 | -19.34 | -- | 44.01 | 48.13 |
|  | Ustekinumab 90 mg Q12W | -- | 5.70 | 5.88 | -- | 24.12 | -31.50 | -- | -21.40 | -20.25 | -- | -34.63 | -30.62 |
|  | Filgotinib 200 mg QD | -- | -4.39 | -5.45 | -- | -14.78 | -15.36 | -- | -12.93 | -12.92 | -- | -19.21 | -9.84 |
|  | Infliximab 5 mg/kg Q8W | -- | -4.69 | -5.31 | -- | -18.51 | -20.19 | -- | -22.48 | -22.24 | -- | 24.80 | 41.89 |
|  | Tofacitinib 10 mg BID | -- | -6.01 | -6.55 | -- | 21.39 | -37.30 | -- | -19.01 | -18.67 | -- | 44.89 | 45.02 |
|  | Infliximab 10 mg/kg Q8W | -- | -6.81 | -6.57 | -- | -18.16 | -18.66 | -- | -20.02 | -20.56 | -- | -35.80 | -33.58 |
|  | Golimumab 50 mg Q4W | -- | -7.78 | -7.71 | -- | -18.06 | -22.22 | -- | -18.41 | -18.38 | -- | -29.48 | -25.81 |
|  | Golimumab 100 mg Q4W | -- | -8.01 | -7.69 | -- | -13.70 | -12.47 | -- | -12.00 | -12.52 | -- | -29.31 | -25.16 |
|  | Adalimumab 40 mg Q2W6 | -- | -8.13 | -8.26 | -- | -16.67 | -14.31* | -- | -9.66* | -10.15 | -- | 43.80 | 44.36 |
|  | Ozanimod 0.92 mg QD | -- | -8.24 | -8.03 | -- | 16.81 | -19.00 | -- | -23.34 | -20.30 | -- | 45.45 | 50.94 |
|  | Adalimumab 40 mg QW | -- | NA | NA | -- | NA | NA | -- | NA | NA | -- | 48.82 | 46.45 |

* Denotes associated NNH 95% CrI excludes absolute values below 1.

† NNHs were obtained by taking the inverse of the absolute safety event rate of UPA minus that of the comparator for the treatment phase; treatments are ordered by descending NNHs (UPA45 or UPA30 vs. comparator) for any AEs by treatment phase.

‡ Outcomes of maintenance treatment among induction responders.

1. SC 200mg at Week 0 and 100mg at Week 2.

2. IV dose based on body weight (~6mg/kg) at Week 0.

3. SC 160mg at Week 0 and 80mg at Week 2, then 40mg Q2W.

4. IV doses at Week 0, 2, and 6.

5. Oral 0.23mg QD for 4 days, 0.46mg QD for 3 days, then 0.92mg QD starting on Day 8.

6. For treat-through trials M10-447 and ULTRA-2 for adalimumab, induction numbers were subtracted from the overall numbers to obtain maintenance numbers.

**Abbreviations**: AE=adverse event; BID=twice daily; CrI=credible interval; NA=not available; NNH=number needed to harm; PBO=placebo; Q#W=every # week; QD=once daily.

## Appendix 14: Supplementary References

36. NICE Guidance. Appendix A: Literature search strategies. Accessed September 07, 2021. https://www.nice.org.uk/guidance/ng50/documents/search-strategies

37. Rutgeerts P, Sandborn WJ, Feagan BG, et al. Infliximab for induction and maintenance therapy for ulcerative colitis. *N Engl J Med*. Dec 8 2005;353(23):2462-76. doi:10.1056/NEJMoa050516

38. Feagan BG, Rutgeerts P, Sands BE, et al. Vedolizumab as induction and maintenance therapy for ulcerative colitis. *N Engl J Med*. Aug 22 2013;369(8):699-710. doi:10.1056/NEJMoa1215734

39. Sandborn WJ, Colombel J-F, Panaccione R, et al. Deep Remission With Vedolizumab in Patients With Moderately to Severely Active Ulcerative Colitis: A GEMINI 1 post hoc Analysis. *Journal of Crohn's and Colitis*. 2018;13(2):172-181. doi:10.1093/ecco-jcc/jjy149

40. Feagan BG, Rubin DT, Danese S, et al. Efficacy of Vedolizumab Induction and Maintenance Therapy in Patients With Ulcerative Colitis, Regardless of Prior Exposure to Tumor Necrosis Factor Antagonists. *Clinical gastroenterology and hepatology : the official clinical practice journal of the American Gastroenterological Association*. 2017/02// 2017;15(2):229-239.e5. doi:10.1016/j.cgh.2016.08.044

41. Kobayashi T, Suzuki Y, Motoya S, et al. First trough level of infliximab at week 2 predicts future outcomes of induction therapy in ulcerative colitis-results from a multicenter prospective randomized controlled trial and its post hoc analysis. *J Gastroenterol*. Mar 2016;51(3):241-51. doi:10.1007/s00535-015-1102-z

42. Jiang XL, Cui HF, Gao J, Fan H. Low-dose Infliximab for Induction and Maintenance Treatment in Chinese Patients With Moderate to Severe Active Ulcerative Colitis. *J Clin Gastroenterol*. Aug 2015;49(7):582-8. doi:10.1097/mcg.0000000000000319

43. Suzuki Y, Motoya S, Hanai H, et al. Efficacy and safety of adalimumab in Japanese patients with moderately to severely active ulcerative colitis. *J Gastroenterol*. Feb 2014;49(2):283-94. doi:10.1007/s00535-013-0922-y

44. Xi'an Janssen Pharmaceutical Ltd. A Phase 3, Multicenter, Randomized, Double-Blind, Placebo-Controlled Study Evaluating the Efficacy and Safety of Infliximab in Chinese Subjects With Active Ulcerative Colitis Clinical Study Report REMICADEUCO3001. ClinicalTrials.gov identifier: NCT01551290. Updated October 21, 2014. Accessed September 08, 2021. http://filehosting.pharmacm.com/DownloadService.ashx?client=CTR_JNJ_7051&studyid=3324&filename=REMICADEUCO3001-Synopsis.pdf

45. Motoya S, Watanabe K, Ogata H, et al. Vedolizumab in Japanese patients with ulcerative colitis: A Phase 3, randomized, double-blind, placebo-controlled study. *PLoS One*. 2019;14(2):e0212989. doi:10.1371/journal.pone.0212989

46. Nagahori M, Watanabe K, Motoya S, et al. Week 2 Symptomatic Response with Vedolizumab as a Predictive Factor in Japanese Anti-TNFα-Naive Patients with Ulcerative Colitis: A post hoc Analysis of a Randomized, Placebo-Controlled Phase 3 Trial. *Digestion*. 2021;102(5):742-752. doi:10.1159/000512235

47. Sandborn WJ, Su C, Sands BE, et al. Tofacitinib as Induction and Maintenance Therapy for Ulcerative Colitis. *N Engl J Med*. May 4 2017;376(18):1723-1736. doi:10.1056/NEJMoa1606910

48. Lichtenstein GR, Moore GT, Soonasra A, et al. P479 Analysis of haematological changes in tofacitinib-treated patients with ulcerative colitis across Phase 3 induction and maintenance studies. *Journal of Crohn's and Colitis*. 2019;13(Supplement_1):S351-S352. doi:10.1093/ecco-jcc/jjy222.603

49. Dubinsky MC, Peyrin-Biroulet L, Melmed GY, et al. Efficacy of Tofacitinib in Patients With Ulcerative Colitis by Prior Tumor Necrosis Factor Inhibitor Treatment Status: Results From OCTAVE Induction and Maintenance Studies: 640. *The American Journal of Gastroenterology*. 2017;112

50. Sandborn W, Sands B, Steinwurz F, et al. Evaluation of the Efficacy of Tofacitinib in Patients with Ulcerative Colitis Utilizing the Modified Mayo Score: Data from the Octave Program. Conference Abstract. *Gastroenterology*. February 2020;158 (3 Supplement):S29. doi:http://dx.doi.org/10.1053/j.gastro.2019.11.098

51. Sandborn WJ, Peyrin-Biroulet L, Sharara AI, et al. Efficacy and Safety of Tofacitinib in Ulcerative Colitis Based on Prior Tumor Necrosis Factor Inhibitor Failure Status. *Clinical Gastroenterology & Hepatology*. 2022;20(3):591-601.e8. doi:10.1016/j.cgh.2021.02.043

52. D'Haens GR, Sands BE, Sandborn WJ, et al. Tofacitinib has induction efficacy in moderately to severely active ulcerative colitis, regardless of prior TNF inhibitor therapy. Conference Abstract. *United European Gastroenterology Journal*. October 2016;4 (5 Supplement 1):A45-A46. doi:http://dx.doi.org/10.1177/2050640616663688

53. Hanauer S, Panaccione R, Danese S, et al. Tofacitinib Induction Therapy Reduces Symptoms Within 3 Days for Patients With Ulcerative Colitis. Research Support, Non-U.S. Gov't. *Clinical Gastroenterology & Hepatology*. 01 2019;17(1):139-147. doi:https://dx.doi.org/10.1016/j.cgh.2018.07.009

54. Chiorean M, Rubin DT, Lo wenberg M, et al. Improvement in physician's global assessment within 2 weeks in patients with ulcerative colitis treated with tofacitinib. Conference Abstract. *United European Gastroenterology Journal*. October 2018;6 (8 Supplement):A448-A449. doi:http://dx.doi.org/10.1177/2050640618792819

55. Danese S, Sandborn W, Panes J, et al. OC.06.1: Onset of Efficacy of Tofacitinib for Induction Therapy in Patients with Active Ulcerative Colitis in two Multinational, Phase 3 Clinical Trials. *Digestive and Liver Disease*. 03/01 2017;49:e90-e91. doi:10.1016/S1590-8658(17)30328-6

56. Sands BE, Long MD, Reinisch W, et al. Tofacitinib for the Treatment of Ulcerative Colitis: Analysis of Nonmelanoma Skin Cancer Rates From the Ulcerative Colitis Clinical Program. *Inflammatory bowel diseases*. 2021;28(2);234-245

57. Vavricka SR, Greuter T, Cohen BL, et al. DOP85 Corticosteroid-free efficacy and safety outcomes in patients receiving tofacitinib in the OCTAVE Sustain maintenance study. *Journal of Crohn's and Colitis*. 2021;15(Supplement_1):S116-S117. doi:10.1093/ecco-jcc/jjab073.124

58. Reinisch W, Osterman M, Doherty G, et al. Tu1720 – Efficacy of Tofacitinib Maintenance Therapy for Ulcerative Colitis in Remitting Patients Vs Patients with Clinical Response After 8 Weeks of Induction Treatment. *Gastroenterology*. 05/01 2019;156:S-1098. doi:10.1016/S0016-5085(19)39706-9

59. Feagan BG, Vermeire S, Sandborn WJ, et al. Tofacitinib for Maintenance Therapy in Patients With Active Ulcerative Colitis in the Phase 3 OCTAVE Sustain Trial: Results by Local and Central Endoscopic Assessments: 607. *Official journal of the American College of Gastroenterology | ACG*. 2017;112:pS329-330.

60. Gary L, Benjamin C, Laurent P-B, et al. P036 Impact of prior immunosuppressant and tumor necrosis factor inhibitor therapies on tofacitinib efficacy and safety in patients with ulcerative colitis. *Official journal of the American College of Gastroenterology | ACG*. 2019;114

61. Hudesman D, Torres J, Salese L, et al. Patient-reported outcome improvement with tofacitinib in the ulcerative colitis octave clinical program. Journal: Conference Abstract. *American journal of gastroenterology*. 2021;116(SUPPL):S404-S405.

62. Hibi T, Imai Y, Senoo A, Ohta K, Ukyo Y. Efficacy and safety of golimumab 52-week maintenance therapy in Japanese patients with moderate to severely active ulcerative colitis: a phase 3, double-blind, randomized, placebo-controlled study-(PURSUIT-J study). *J Gastroenterol*. Oct 2017;52(10):1101-1111. doi:10.1007/s00535-017-1326-1

63. Schreiber S, Armuzzi A, Dignass A, et al. Corticosteroid-Free Remission In Patients With Moderately To Severely Active Ulcerative Colitis Treated With Ozanimod: Results From The Maintenance Phase Of True North. Conference Abstract. *Gastroenterology*. May 2021;160(6 Supplement):S-83. doi:https://dx.doi.org/10.1016/S0016-5085%2821%2900944-6

64. Sandborn WJ, Feagan BG, Marano C, et al. Subcutaneous golimumab maintains clinical response in patients with moderate-to-severe ulcerative colitis. *Gastroenterology*. Jan 2014;146(1):96-109.e1. doi:10.1053/j.gastro.2013.06.010

65. Sandborn WJ, Feagan BG, Marano C, et al. Subcutaneous golimumab induces clinical response and remission in patients with moderate-to-severe ulcerative colitis. *Gastroenterology*. Jan 2014;146(1):85-95; quiz e14-5. doi:10.1053/j.gastro.2013.05.048

66. Feagan BG, Danese S, Loftus EV, Jr., et al. Filgotinib as induction and maintenance therapy for ulcerative colitis (SELECTION): a phase 2b/3 double-blind, randomised, placebo-controlled trial. *Lancet (London, England)*. Jun 19 2021;397(10292):2372-2384. doi:10.1016/s0140-6736(21)00666-8

67. Loftus E, Vermeire S, Feagan B, et al. DOP82 Corticosteroid-free remission of Ulcerative Colitis with filgotinib maintenance therapy: Post hoc analysis of the phase 2b/3 SELECTION study. *Journal of Crohn's and Colitis*. 2021;15(Supplement_1):S112-S113. doi:10.1093/ecco-jcc/jjab073.121

68. Peyrin-Biroulet L, Dotan I, Hibi T, et al. OP25 Efficacy of filgotinib in patients with Ulcerative Colitis by line of therapy in the phase 2b/3 SELECTION trial. *Journal of Crohn's and Colitis*. 2021;15(Supplement_1):S024-S026. doi:10.1093/ecco-jcc/jjab075.024

69. Schreiber SW, Watanabe M, Yun C, et al. OP04 Safety analysis of filgotinib for Ulcerative Colitis: Results from the phase 2b/3 SELECTION study and phase 3 SELECTIONLTE long-term extension study. *Journal of Crohn's and Colitis*. 2021;15(Supplement_1):S004-S006. doi:10.1093/ecco-jcc/jjab075.003

70. Vermeire S, Oortwijn A, Feagan BG, et al. Early Achievement of Partial Mayo Score Remission and Ibdq Normalization in Patients with Ulcerative Colitis Treated with Filgotinib in the Phase 2b/3 Selection Study. Conference Abstract. *Gastroenterology*. May 2021;160(6 Supplement):S-352-S-353. doi:https://dx.doi.org/10.1016/S0016-5085%2821%2901558-4

71. Feagan BG, Loftus EV, Danese S, et al. Efficacy and Safety of Filgotinib as Induction Therapy for Patients with Moderately to Severely Active Ulcerative Colitis: Results from the Phase 2b/3 Selection Study. Conference Abstract. *Gastroenterology*. May 2021;160(6 Supplement):S-125. doi:https://dx.doi.org/10.1016/S0016-5085%2821%2901052-0

72. Peyrin-Biroulet L, Loftus EV, Danese S, et al. Efficacy and Safety of Filgotinib as Maintenance Therapy for Patients with Moderately to Severely Active Ulcerative Colitis: Results from the Phase 2b/3 Selection Study. Conference Abstract. *Gastroenterology*. May 2021;160(6 Supplement):S-350. doi:https://dx.doi.org/10.1016/S0016-5085%2821%2901552-3

73. Peyrin-Biroulet L, Loftus EV, Hibi T, et al. Relationship between histo-endoscopic mucosal healing and baseline characteristics in patients with moderately to severely active ulcerative colitis receiving filgotinib in the phase 2b/3 selection study. Conference Abstract. *United European Gastroenterology Journal*. October 2021;9(SUPPL 8):518-519. doi:http://dx.doi.org/10.1002/ueg2.12144

74. Panes J, Colombel JF, D'Haens GR, et al. High versus standard adalimumab induction dosing regime ns in patients with moderately to severely active ulcerative colitis : Results from the SERENE-UC induction study. Conference Abstract. *United European Gastroenterology Journal*. October 2019;7 (8 Supplement):118. doi:http://dx.doi.org/10.1177/205064061985467

75. Colombel JF, Panes J, D'Haens GR, et al. 945 Higher Versus Standard Adalimumab Maintenance Regimens in Patients with Moderately to Severely Active Ulcerative Colitis: Results from the Serene-Uc Maintenance Study. Conference Abstract. *Gastroenterology*. May 2020;158 (6 Supplement 1):S-192. doi:http://dx.doi.org/10.1016/S0016-5085%2820%2931162-8

76. Sandborn WJ, Feagan BG, D'Haens G, et al. Ozanimod as Induction and Maintenance Therapy for Ulcerative Colitis. Clinical Trial, Phase III Multicenter Study Randomized Controlled Trial Research Support, Non-U.S. Gov't. *New England Journal of Medicine*. 09 30 2021;385(14):1280-1291. doi:https://dx.doi.org/10.1056/NEJMoa2033617

77. William S, Geert D, Doug W, et al. P025 Ozanimod Efficacy, Safety, and Histology in Patients with Moderate-to-Severe Ulcerative Colitis During Induction in the Phase 3 True North Study. *The American journal of gastroenterology*. 01 Dec 2020;115(Supplement 1):S6-S7. doi:http://dx.doi.org/10.14309/01.ajg.0000722896.32651.d6

78. Silvio D, Brian F, Stephen H, et al. P030 Ozanimod Efficacy, Safety, and Histology in Patients with Moderate-to-Severe Ulcerative Colitis During Maintenance in the Phase 3 True North Study. *Am J Gastroenterol*. Dec 1 2020;115(Suppl 1):S8. doi:10.14309/01.ajg.0000722916.98351.89

79. Subrata G, Geert D, Vipul J, et al. P012 Ozanimod Reduced Fecal Calprotectin Levels in Patients with Ulcerative Colitis in the Phase 3 True North Study. *The American journal of gastroenterology*. Dec 1 2020;115(Suppl 1):S3. doi:10.14309/01.ajg.0000722844.18503.37

80. Sands BE, Nguyen D, Pondel M, et al. Impact of prior biologic exposure on patient response to ozanimod for moderate-to-severe ulcerative colitis in the phase 3 true north study. Conference Abstract. *American Journal of Gastroenterology*. October 2021;116(SUPPL):S313-S314. doi:http://dx.doi.org/10.14309/01.ajg.0000776304.52681.bf

81. Osterman MT, Longman R, Sninsky C, et al. Rapid Induction Effects of Ozanimod on Clinical Symptoms and Inflammatory Biomarkers in Patients with Moderately to Severely Active Ulcerative Colitis: Results from the Induction Phase of True North. Conference Abstract. *Gastroenterology*. May 2021;160(6 Supplement):S-93-S-94. doi:https://dx.doi.org/10.1016/S0016-5085%2821%2900965-3

82. AbbVie. Clinical Study Protocol M14-675. A Multicenter, Randomized, Double-Blind,

Placebo-Controlled Induction Study to Evaluate the Efficacy and Safety of Upadacitinib (ABT-494) in Subjects with Moderately to Severely Active Ulcerative Colitis. EudraCT Identifier: 2016-000642-62. July 31, 2020.

83. AbbVie. Clinical Study Protocol M14-234. A Multicenter, Randomized, Double-Blind,

Placebo-Controlled Study to Evaluate the Safety and Efficacy of Upadacitinib (ABT-494) for Induction and Maintenance Therapy in Subjects with Moderately to Severely Active Ulcerative Colitis. EudraCT Identifier: 2016-000641-31. July 31, 2020.

84. Reinisch W, Sandborn WJ, Hommes DW, et al. Adalimumab for induction of clinical remission in moderately to severely active ulcerative colitis: results of a randomised controlled trial. *Gut*. Jun 2011;60(6):780-7. doi:10.1136/gut.2010.221127

85. Sandborn WJ, van Assche G, Reinisch W, et al. Adalimumab induces and maintains clinical remission in patients with moderate-to-severe ulcerative colitis. *Gastroenterology*. Feb 2012;142(2):257-65.e1-3. doi:10.1053/j.gastro.2011.10.032

86. Ghosh S, Wolf D, Sandborn W, et al. P568 Sustained efficacy in patients with ulcerative colitis treated with adalimumab: results from ULTRA 2. *Journal of Crohn's and Colitis*. 2013;7(Supplement_1):S238-S238. doi:10.1016/s1873-9946(13)60589-9

87. Colombel JF, Plevy S, Sandborn W, et al. Time to remission and response in adalimumabtreated patients with moderately to severely active ulcerative colitis from ultra 2. Conference Abstract. *United European Gastroenterology Journal*. October 2013;1(1)(1):A219-A220. doi:http://dx.doi.org/10.1177/2050640613502900

88. Sandborn WJ, Wolf DC, Van Assche G, et al. Rapid onset of adalimumab and long-term efficacy among week-8 responders in adults with moderate to severe active Ulcerative Colitis: O-7. *Inflammatory Bowel Diseases*. 2011;17(Suppl_2):S4-S4. doi:10.1097/00054725-201112002-00009

89. D'Haens G, Van Assche G, Wolf D, et al. Mucosal healing in ulcerative colitis patients with week 8 response to adalimumab: Subanalysis of ultra 2. Conference Abstract. *American Journal of Gastroenterology*. October 2012;107(1):S610-S611. doi:http://dx.doi.org/10.1038/ajg.2012.275

90. Sandborn WJ, Colombel JF, D'Haens G, et al. One-year maintenance outcomes among patients with moderately-to-severely active ulcerative colitis who responded to induction therapy with adalimumab: subgroup analyses from ULTRA 2. *Aliment Pharmacol Ther*. Jan 2013;37(2):204-13. doi:10.1111/apt.12145

91. Panaccione R, Colombel J-F, Sandborn W, et al. Sa1229 Durable Clinical Remission and Response in Adalimumab-Treated Patients With Ulcerative Colitis. *Gastroenterology*. 04/01 2015;148(4):S-264. doi:10.1016/S0016-5085(15)30869-6

92. Sands BE, Sandborn WJ, Panaccione R, et al. Ustekinumab as Induction and Maintenance Therapy for Ulcerative Colitis. *N Engl J Med*. Sep 26 2019;381(13):1201-1214. doi:10.1056/NEJMoa1900750

93. Van Assche G, Targan S, Baker T, et al. Sustained remission in patients with moderate to severe ulcerative colitis: Results from the Phase III UNIFI maintenance study. Conference Abstract. *Journal of gastroenterology and hepatology*. September 2019;34 (Supplement 2):154-155. doi:http://dx.doi.org/10.1111/jgh.14801

94. Sands BE, Peyrin-Biroulet L, Marano C, et al. Efficacy in biologic failure and non-biologicfailure populations in a Phase 3 study of ustekinumab in moderate-severe ulcerative colitis: UNIFI. Conference Abstract. *Journal of Crohn's and Colitis*. March 2019;13 (Supplement 1):S256-S257. doi:http://dx.doi.org/10.1093/ecco-jcc/jjy222.436

95. M. Alcalá SS, T. Hernando, I. García. P0516 Efficacy of Ustekinumab At The End of Maintenance in Ulcerative Colitis Patients Receiving 6 Mg/ Kg Induction Posology. Data From Unifi Trial (Ueg Week 2020 Poster Presentations). *United European Gastroenterology Journal*. 2020;8(S8):144-887. doi:https://doi.org/10.1177/2050640620927345

96. Danese S, Sands BE, Sandborn WJ, et al. Efficacy of ustekinumab subcutaneous maintenance treatment by induction-dose subgroup in the unifi study of patients with ulcerative colitis. Conference Abstract. *United European Gastroenterology Journal*. 2019;7 (10):1415. doi:http://dx.doi.org/10.1177/2050640619888859

97. Panaccione R, Peyrin-Biroulet L, Danese S, et al. Impact of response and inflammatory burden at start of maintenance therapy on clinical efficacy of ustekinumab dosing regimen in UC: Week 44 results from UNIFI. Conference Abstract. *United European Gastroenterology Journal*. October 2019;7 (8 Supplement):93-94. doi:http://dx.doi.org/10.1177/205064061985467

98. Sandborn WJ, Baert F, Danese S, et al. Efficacy and Safety of Vedolizumab Subcutaneous Formulation in a Randomized Trial of Patients With Ulcerative Colitis. *Gastroenterology*. 2020;158(3):562-572.e12. doi:10.1053/j.gastro.2019.08.027
